# CelltypeR: A flow cytometry pipeline to characterize single cells from brain organoids

**DOI:** 10.1016/j.isci.2024.110613

**Published:** 2024-07-30

**Authors:** Rhalena A. Thomas, Julien Sirois, Shuming Li, Alexandre Gestin, Ghislaine Deyab, Valerio E.C. Piscopo, Paula Lépine, Meghna Mathur, Carol X.-Q. Chen, Vincent Soubannier, Taylor M. Goldsmith, Lama Fawaz, Thomas M. Durcan, Edward A. Fon

**Affiliations:** 1 Department of Neurology and Neurosurgery, Montreal Neurological Institute-Hospital, McGill University, Montreal, QC H3A 2B4, Canada; 2 The Neuro's Early Drug Discovery Unit (EDDU), McGill University, Montreal, QC H3A 2B4, Canada; 3 Université Paris-Saclay, 91190 Gif-sur-Yvette, France

**Keywords:** Neuroscience, Cell biology, Omics

## Abstract

Motivated by the cellular heterogeneity in complex tissues, particularly in brain and induced pluripotent stem cell (iPSC)-derived brain models, we developed a complete workflow to reproducibly characterize cell types in complex tissues. Our approach combines a flow cytometry (FC) antibody panel with our computational pipeline CelltypeR, enabling dataset aligning, unsupervised clustering optimization, cell type annotating, and statistical comparisons. Applied to human iPSC derived midbrain organoids, it successfully identified the major brain cell types. We performed fluorescence-activated cell sorting of CelltypeR-defined astrocytes, radial glia, and neurons, exploring transcriptional states by single-cell RNA sequencing. Among the sorted neurons, we identified subgroups of dopamine neurons: one reminiscent of substantia nigra cells most vulnerable in Parkinson’s disease. Finally, we used our workflow to track cell types across a time course of organoid differentiation. Overall, our adaptable analysis framework provides a generalizable method for reproducibly identifying cell types across FC datasets in complex tissues.

## Highlights

• CelltypeR is a flow cytometry and computational pipeline for cell type quantification
• Identified brain cell types in midbrain organoids and measured changes in proportions
• Enriched selected populations using FACS and characterized by single-cell RNA sequencing
• Identified substantia nigra–like dopaminergic neurons sensitive in Parkinson’s disease

## Graphical abstract

**Figure:**
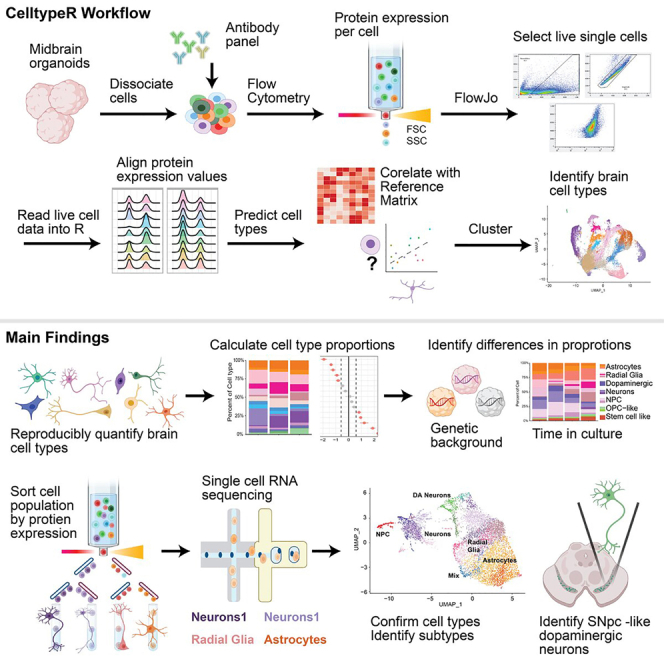

## Introduction

Investigating the molecular, cellular, and tissue properties of the human brain requires the use of cellular models, as live human brain tissue cannot be easily accessed for research. Patient-derived disease 3D tissues, such as human midbrain organoids (hMOs), derived from human induced pluripotent stem cells (iPSCs), provide a promising physiologically relevant model for human brain development and diseases, including neurodegenerative diseases such as Parkinson’s disease (PD).^1^^,^^2^^,^^3^^,^^4^ Yet, as new models emerge, the complexity and reproducibility of these systems needs to be captured to utilize these models in addressing biological questions. To determine how faithfully organoids recapitulate the human brain and how organoids derived from individuals with disease differ from those derived from healthy controls, new approaches toward characterization are required. Effective and quantitative methods are needed to determine the cell types within these complex tissues and to apply these benchmarks reproducibly across experiments. At present, individual cells within brain or organoid tissue can be identified using single-cell RNA sequencing (scRNA-seq) or labeling of protein or RNA in tissue sections. These tools are useful but limited. scRNA-seq is a powerful tool that has been used to identify known and novel cell types, cell states, and cell fate trajectories.^5^^,^^6^^,^^7^ However, using scRNA-seq to compare proportions or populations of cells between genotypes over multiple time points is not practical for hMOs and may result in sampling bias, as less than 1% of the whole tissue is sequenced. While scRNA-seq provides detailed expression values to determine sub-types of cells, only relatively few samples can be run at a given time and all the cells must be alive and prepared in parallel, which can lead to technically challenging experiments. These experiments are also costly for the number of replicates needed to ensure sufficient power for comparing multiple time points, disease states, or pharmacological treatments.^8^^,^^9^^,^^10^ Another option to quantify cell types is immunostaining or *in situ* hybridization of tissue sections. This has the advantage of capturing cell morphology and spatial resolution. However, sample preparation, image acquisition, and analysis are labor intensive and limited in quantitative accuracy. Moreover, for 3D tissues, either only a small section can be analyzed, or the entire tissue must be reconstructed and only a few cell types can be detected at once.^11^^,^^12^

Here, we use flow cytometry (FC) to measure the protein expression levels of a panel of cell surface markers enriched in specific brain cell types. FC is a fast, quantitative, and robust method, used widely in immunology and cancer research,^13^^,^^14^^,^^15^ but to date only sparsely in neuroscience. Typically, in neurobiology, only two or three antibodies are used to distinguish between pairs of cell types^16^^,^^17^ or to enrich one cell type.^18^^,^^19^ Traditional FC analysis methods using commercially available analysis software packages, such as FlowJo (Becton-Dickinson Biosciences), which are time-consuming and subject to user error. There are several excellent R software packages to process FC data including FlowStats^20^ and FlowCore^21^ and to perform unsupervised clustering based on protein expression including CytoTree, FlowSOM, and PhenoGraph.^22^^,^^23^^,^^24^ However, no methods are available to streamline cell type annotation in FC from complex tissues such as brain or 3D brain organoids using a large antibody panel. To create such an analysis framework, we produced an experimental dataset using cultured hMOs differentiated from human iPSCs.^1^^,^^4^^,^^25^ Our workflow also provides the methods to select subtypes of cells and gate these cells for further analysis, such as RNA-seq, proteomics, or enriching cultures. We select example cell populations, sort these cell types, and further characterize these with scRNA-seq. Here, we present a complete framework for annotating cell types within complex tissue and comparing proportions of cell types across conditions and experiments.

## Results

### An antibody panel can be used to identify multiple cell types in hMOs

**Figure 1:**
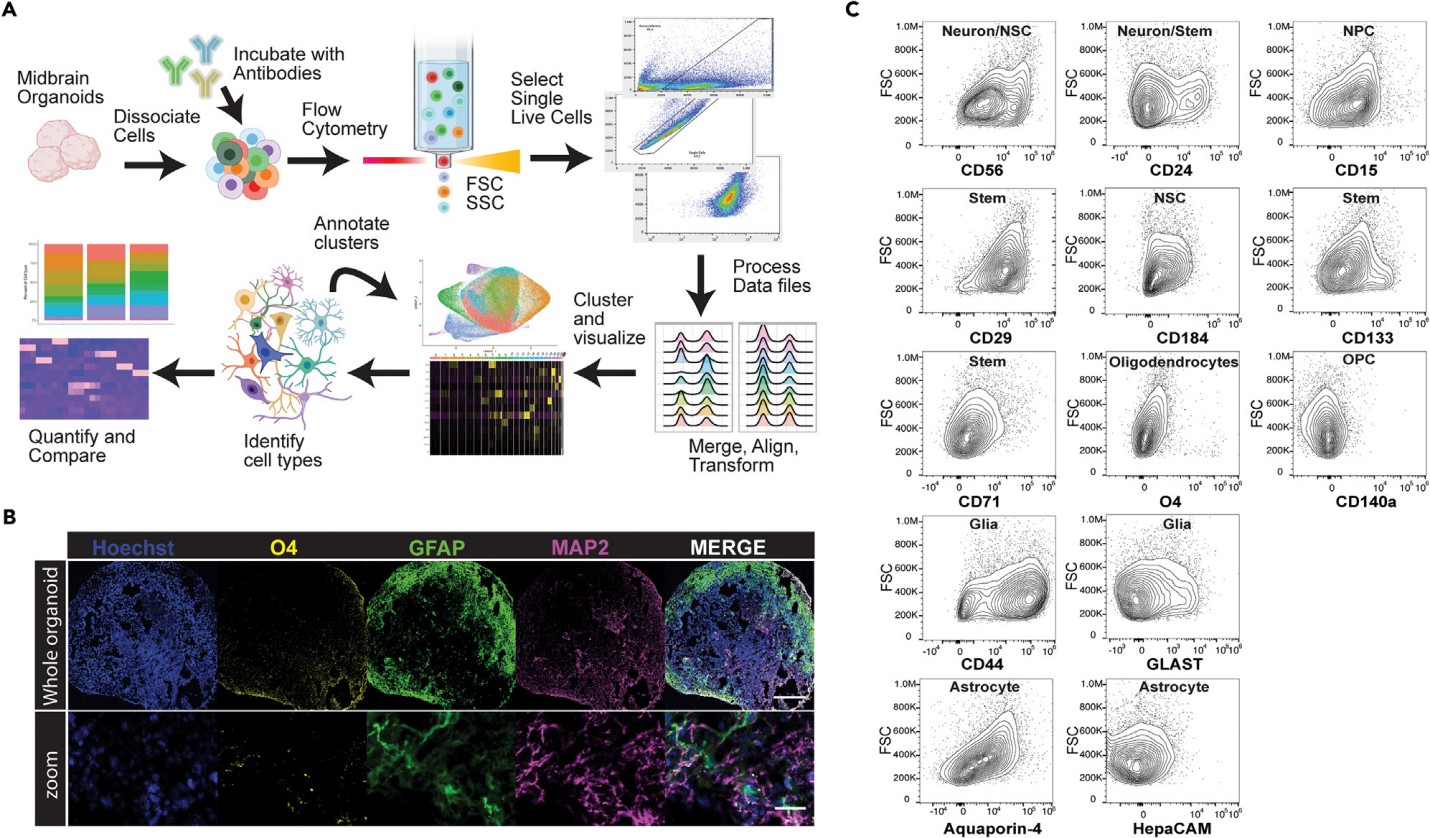
A workflow to identify and quantify cell types in midbrain hMOs using a panel of FC antibodies (A) Schematic of the CelltypeR workflow: tissue (hMO) is dissociated and labeled with an antibody panel, expression levels are measured on individual cells using FC, and live single cells are gated from the debris and doublets in FlowJo. The data are then preprocessed in R, merging files and harmonizing the data if desired. Unsupervised clustering is used to find groups of cell types, methods are provided to aid in cluster annotation, annotated cells are quantified, and statistical analyses are applied. (B) Example image of a cryosection from an AJG001-C4C hMO, 285 days in final differentiation culture, showing total nuclei (Hoechst), oligodendrocytes (O4), astrocytes (GFAP), and neurons (MAP2). Top: cross section of a whole hMO stitched together from tiled images, scale bar = 250μm. Bottom: zoomed in image cropped from the whole hMO image, scale bar = 25μm. (C) Contour plots showing the cell size on the y axis (FSC) and intensity of staining for each antibody in the panel on the x axis (log scale biexponential transformation). See also Figures S1–S3 and Tables 1 and 2.

In Figure 1A, we provide a schematic of the CelltypeR analysis workflow (see STAR Methods) used to quantify and compare cell types from tissues containing a heterogeneous population of cells with a particular focus on neuronal tissue through brain organoids. To test our CelltypeR pipeline, we used hMOs^4^ differentiated from iPSC lines derived from three unrelated healthy individuals. The hMOs were grown for 9 months in culture, a time point at which neurons are expected to be mature and astrocytes and oligodendrocytes have been shown to be present.^1^^,^^26^ Immunofluorescence staining of cryosections showed that these hMOs contain neurons, astrocytes and oligodendrocytes (Figures 1B and S1). In FC, combinations of the relative intensities of 2-3 antibodies are often used to distinguish between cell types. However, in hMOs we expect approximately nine cellular types with a continuum of stages of differentiation.^1^^,^^4^^,^^27^ We first defined a panel of 13 antibodies, which included well-characterized antibodies previously used in FC to define neural stem cells, neurons, astrocytes, and oligodendrocytes or to define other cell types in cultured immortalized human cell lines, blood, or brain tissues (Table 1).^16^^,^^19^^,^^28^^,^^29^^,^^30^^,^^31^^,^^32^^,^^33^^,^^34^^,^^35^^,^^36^ We dissociated the mature hMOs and labeled the cell suspension with these antibodies then measured the fluorescence intensity values corresponding to the protein targets using FC. The hMOs used were cultured in final differentiation media for over 9 months and were sensitive to cell loss during dissociation and antibody incubations. Live/dead staining showed the live cell recovery after dissociated ranged from 39.6 to 81.5% (see Table S14 for all event and cell counts). Live single live cells were gated using FlowJo prior to further analysis (Figure S2). The FC results show that each protein has a range of expression across different cells (Figures 1C and S3A). We conclude that the antibody panel has the potential to define cell types by identifying combinations of protein expression profiles unique to different cell groups.

**Table 1:** Antibody panel with cell types previously reported to be identified by each marker

Antibody/Marker	Protein/Gene	Reported Cell type marker	References
CD24	CD24	Neurons and neural stem cells Cancer stem cells	Uchida 2000,^30^ Pruszak 2007,^16^ Pruszak 2009,^28^ Sundberg 2009,^32^ Yuan 2011,^19^ Wang 2013^31^
CD56	NCAM1	Neurons and neural stem cells Cancer cells	Pruszak 2007,^16^ Pruszak 2009,^28^ Sundberg 2009^32^
CD29	ITGB1	Stem cell	Pruszak,^16^ Yuan 2011,^19^
CD15	FUT4	Neural precursor	Pruszak 2007,^16^ Pruszak 2009,^28^ Yuan 2011,^19^ Sandor 2017^29^
CD184	CXCR4	Neural stem cell	Yuan 2011,^19^ Sandor 2017^29^
CD133	PROM1	Stem cell	Uchida 2000,^30^ Pruszak 2007,^16^ Barraud 2007,^33^ Pruszak 2009,^28^
CD71	TFRC	Stem cell	Pruszak 2007,^16^
CD44	CD44	Glia	Liu 2004,^37^ Yuan 2011,^19^
GLAST	GLAST/SLC1A3	Glia	Liu 2004,^37^ Jurga 2021^34^
AQP4	AQP4	Astrocyte	Wang 2013,^31^ Jurga 2021^34^
HepaCAM	HEPACAM	Astrocyte	Heiland 2019^35^
CD140a	PDGFRA	OPC	Liu 2004,^37^ Wang 2013^31^
O4	Gene unknown	Oligodendrocyte	Liu 2004,^37^ McPhie 2018^36^

### Analysis of 2D cultures reveals cell type specific expression profiles and identifies subgroups of cell types within cultures

**Figure 2:**
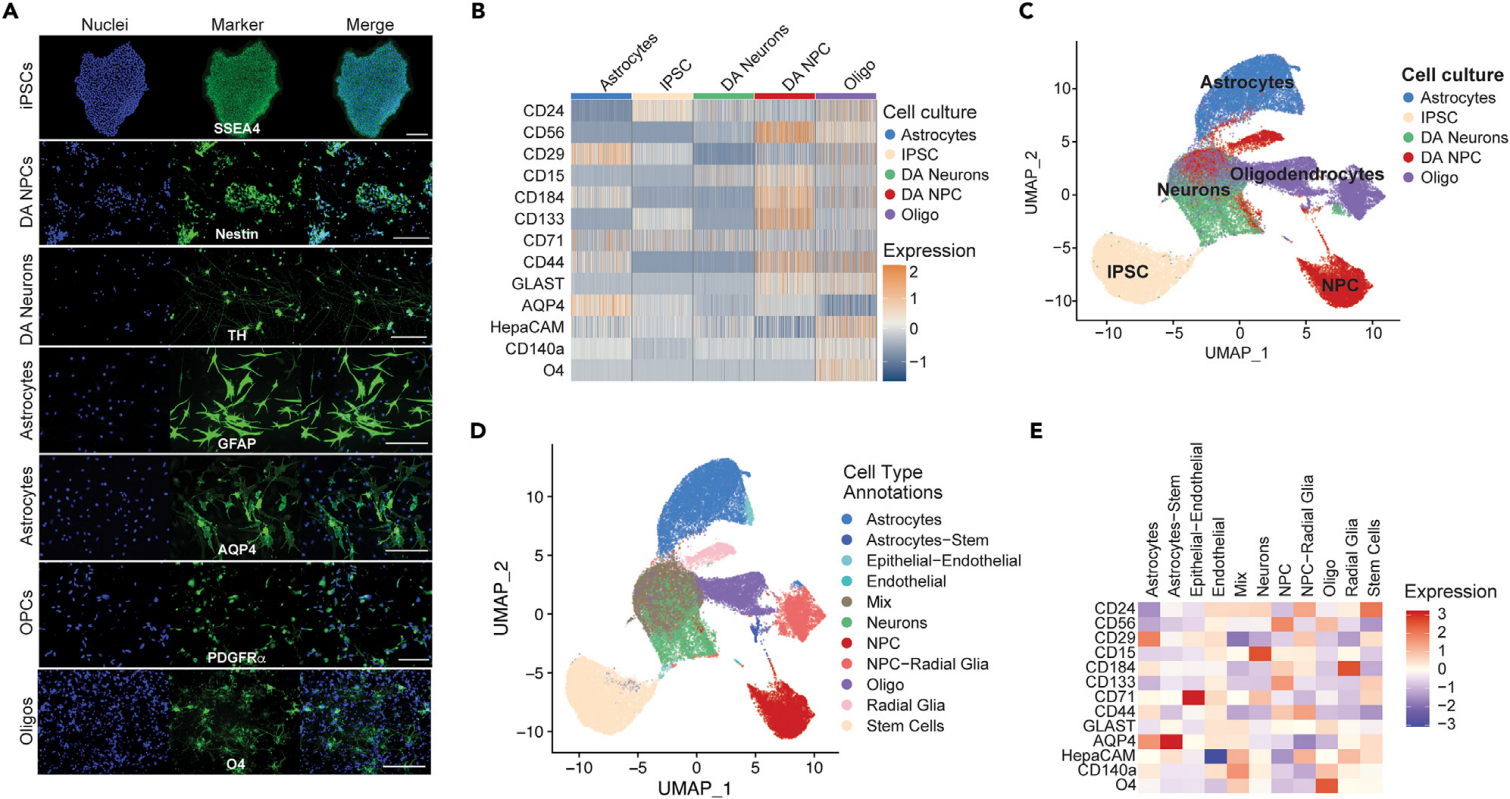
The antibody panel can be used to identify cell types expected to be present in hMOs (A) Example images of different brain cell types (indicated on the left) derived from the healthy control AIW002-02-02 human iPSC line and individually differentiated. Cell cultures were stained with a cell type specific marker (green) and Hoechst (blue) for nuclei. Scale bars 200μM. (B) Heatmap of the normalized and z-scored protein levels measured by FC (area under the curve) for a subset of cells from each cell culture (indicated above). The marker proteins are indicated on the left. Each bar represents a single cell, 200 randomly selected cells are shown. Expression values are normalized setting the mean expression to 0 and the standard deviation to 1. (C) Visualization of expression profiles of single cells using Uniform Manifold Approximation and Projection (UMAP) for dimensionality reduction of marker expression values, where each dot represents a cell. The data shown are the different cell cultures merged together. The original cell cultures are indicated by color, showing the separation and overlap of cell types from within different 2D cultures. (D) The same UMAP with annotated clusters identified by Louvain network detection. The annotated cell types are labeled by color indicated in the legend. (E) Heatmap of the mean expression of each protein within the cell subgroups identified by clustering. Expression values are normalized setting the mean expression to 0 and the standard deviation to 1. FC measurements were acquired on two experimental days, astrocytes, DA NPCs and oligodendrocyte cultures used on both experiment days (1 = 06/03/2020, 2 = 17/03/2020). DA neurons, were measured on experiment day 1 and iPSC were measured on day 2. The data from both days were pooled and then cells were randomly down sampled to 10000 cells per culture type, *n* = 50000 cells. See also Figures S4 and S5.

To validate the expression of the proteins targeted by the selected antibodies on known cell types, we separately differentiated iPSCs into dopaminergic neuronal precursor cells (DA NPCs),^25^ dopaminergic neurons (DA neurons),^38^ astrocytes,^39^ and oligodendrocytes^36^ (Figure 2A). The cultures were dissociated and the 13 antibodies in the FC panel were applied. We examined the staining for each antibody across the cultured 2D cells (Figure 2B). As expected, we see high expression CD24 in the iPSC cultures.^40^ We also observe high CD24 and other marker levels in oligodendrocytes. The relative expression of CD56 and CD24 in the neuronal culture was lower than expected (Figures 2B and S3B). Within each cell type there was a variation in protein expression levels that could be used to define subgroups of cells. To identify subgroups of cells and visualize the markers, we applied unsupervised clustering developed as part of the CelltypeR workflow. Some tools exist for automated processing and formatting of FC^20^^,^^21^ and numerous tools exist for cluster analysis of single cell data that can be applied to FC data.^22^^,^^23^^,^^41^ Thus, we took advantage of some of these existing tools and created new functions in an R package to process FC data (see STAR Methods). We combined the FC acquired protein expression levels from the five separate iPSC derived cultures, normalized the data, and performed dimensional reductions. The UMAP visualization shows separate groups for each of the five cell types with some overlap (Figure 2C). The iPSCs are separate from all other cell cultures. Whereas the NPC culture splits into separate groups and overlaps with different cell types, the same is observed for the oligodendrocyte culture (Figure S4A). Clustering analysis identifies subgroups of cell types and some clusters with cells from multiple 2D cell cultures (Figures 2D and S4B). The DA NPC culture is an intermediate stage between iPSC and the three other cell cultures; therefore, it is not surprising that the cells from the NPC culture cluster together with other cell cultures. In the oligodendrocyte culture there is one cluster with the highest O4 expression that represents the oligodendrocytes within the culture (Figures 2E and S5). We conclude from these findings using iPSC-derived 2D cultures that our antibody panel can distinguish different cell types and subgroups of cells that we expect to find in 3D hMOs and other complex neuronal tissues.

### CelltypeR can be used to identify different brain cell types in hMOs

To identify cell types within hMOs using the antibody panel, we ran our R preprocessing pipeline to align and normalize the data. To compare samples from different iPSC lines, different batches of hMOs, and measurements run on different experiment days, we developed methods to combine and harmonize samples. This is the first step in the computational pipeline. We combined nine hMO samples and selected a subset of the total cells, 9000 cells or the max number of cells available from each hMO sample (Table 2). The samples were first merged, then transformed and aligned to reduce batch effects, and finally retro-transformed for better cluster visualization (Figure S6). If removing batch effects is not desired (as in the separate cell cultures above), the preprocessing is stopped after merging. The hMOs contain a combination of neurons, NPCs, astrocytes, oligodendrocyte precursors (OPCs), oligodendrocytes, radial glia, stem cells, pericytes, endothelial cells, and epithelial cells, all differentiated from the starting iPSCs.^4^^,^^26^^,^^42^ The standard method of manually defining cell groups using FlowJo or multiple scatterplots in R is time consuming and only permits a visual comparison of two marker combinations at once. Manually defining thresholds for each antibody and recording those thresholds is also prone to error and doesn’t account for a change in distribution of staining across samples. To overcome this barrier, we developed tools to identify cell types described below: (1) A correlation cell type assignment model (CAM) using a custom reference matrix and (2) clustering parameter exploration functions with tools to visualize and summarize protein expression levels.

**Table 2:** Description of hMO datasets

iPSC line	Batch date	Batch	Days in culture	FC acquisition date	hMO per tube	Tech rep	#live cells	Experiment
AIW002-02	30/05/2019	A	273	06/03/2020	3	1	43941	Quantify cells
AIW002-02	20/06/2019	B	263	17/03/2020	3	1	35833	Quantify cells
AIW002-02	30/05/2019	A	284	17/03/2020	3	1	9071	Quantify cells
AJG001-C4	30/05/2019	A	273	06/03/2020	3	1	34031	Quantify cells
AJG001-C4	20/06/2019	B	263	17/03/2020	3	1	15049	Quantify cells
AJG001-C4	30/05/2019	A	284	17/03/2020	3	1	1578	Quantify cells
3450	30/05/2019	A	273	06/03/2020	3	1	30404	Quantify cells
3450	20/06/2019	B	263	17/03/2020	3	1	9205	Quantify cells
3450	30/05/2019	A	284	17/03/2020	3	1	18048	Quantify cells
AIW002-02	27/08/2021	C	273	10/05/2022	40	2	60017	FACS sort
AIW002-02	02/08/2021	D	246	10/05/2022	40	2	60458	FACS sort
AIW002-02	27/08/2021	C	304	10/06/2022	20	3	81923	FACS sort & scRNAseq
AIW002-02	06/12/2021	E	38	21/01/2022	8	4	226210	Time course
AIW002-02	06/12/2021	E	63	15/02/2022	8	4	327811	Time course
AIW002-02	06/12/2021	E	98	22/03/2022	8	4	221598	Time course
AIW002-02	06/12/2021	E	155	18/05/2022	8	4	474805	Time course

The date of seeding iPSCs, 8 days before final differentiation is indicated. Days in culture is the time between transfer to final differentiation and FC. The date of dissociation, labeling and acquisition (FC acquisition date) is indicated.

**Figure 3:**
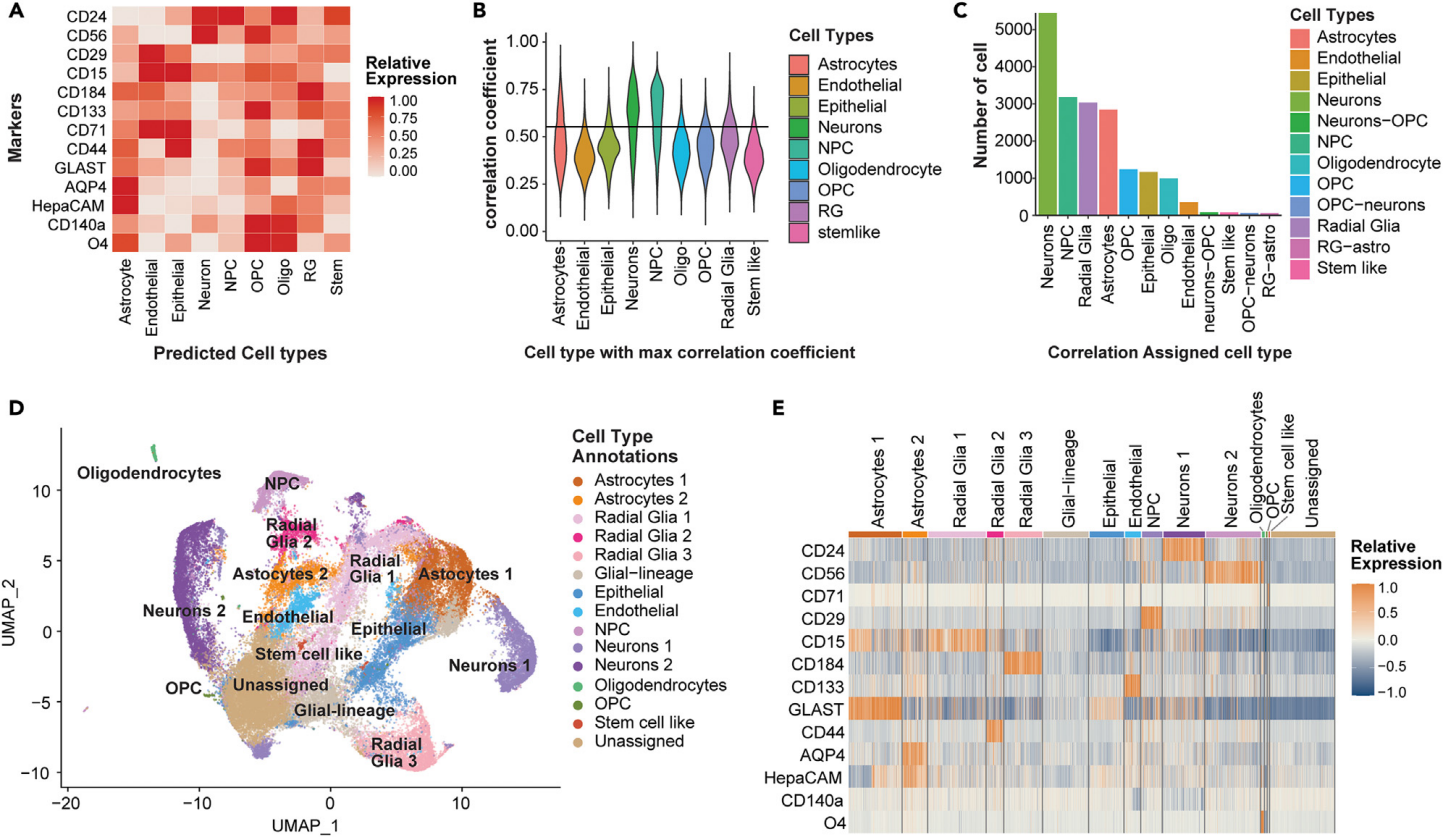
Identification of cell types in hMO using the FC antibody panel (A) Heatmap of predicted relative expression of each antibody in the FC panel for each potential cell type in hMOs. Values are calculated from 2D FC intensities, scRNA-seq from hMOs and human brain, and RNA-seq from human brain. The values are z-scored and scaled between 0 and 1. (B) Violin plot showing the distribution of R values for hMO cells (y axis) with the indicated potential brain cell type (x axis). The R values are plotted for the cell type with the max R value. The black line indicates the threshold of R = 0.553 which was set as the cut-off for assigning a cell type prediction. (C) Bar chart showing the number of hMO cells categorized as each cell type by the max correlation. Each cell type is indicated on the x axis. hMO cells were assigned as a double cell type if the first and second max R values were within 0.05. Only cell assignments with over 100 cells are included in the bar chart. (D) UMAP showing unsupervised clustering by Louvain network detection using principal component analysis of the FC expression levels as input. Cell types were annotated using a combination of CAM and expert analysis of expression within clusters. (E) Heatmap of relative expression of each antibody grouped by the cell types identified by unsupervised clustering of hMO cells. A subset of cells from each cluster relative to cluster size are shown (up to 200 cells), where each bar on the y axis is one cell. Expression values are normalized setting the mean expression to 0 and the standard deviation to 1. Three hMO from each genotype (AIW002-02, 3450, and AJG001C) from 2 batches (A and B) on two different experiment days were used. A total of 9 hMO samples, with 9000 cells per hMO except for one AJG001C sample. All plots show results from the 9 merged samples. See also Figures S6–S10 and Tables 2 and S1.

We created a reference matrix with the predicted relative expression of each cell surface marker in different cell types expected to be present in hMOs based on previous hMO and human brain data. Using scRNA-seq data from human brain^43^^,^^44^^,^^45^^,^^46^^,^^47^^,^^48^ and organoids,^1^^,^^49^ total mRNA on brain cell types,^43^ and FC (Table S1), we calculated the relative expression levels for each protein marker in our antibody panel (Figure 3A). The CAM function calculates the correlation of protein expression levels of the 13 markers in each hMO-derived cell to the expression levels of the same markers in the reference matrix we created, calculating the Pearson correlation coefficient (R). The R value is calculated for each cell type in the reference matrix. The cell type with the highest R value, above an adjustable threshold, out of the nine possible cell types is assigned for a given hMO derived cell (Figure 3B). Cells with R values below the selected cut-off are left unassigned. The FC panel contains 13 markers used as comparison points, thus an R value of 0.553 is required for a statistically significant correlation (*p* < 0.05). Applying this significance threshold, neurons are the most assigned cell type (Figure 3C). With an R cut-off of 0.553 the majority of cells are left assigned. The number of assigned cells depends on the R threshold and using a cut-off of 0.1 all cells are assigned a cell type prediction; however, these predictions could be less accurate (Figure S7). Some hMO-derived cells correlated close to equally (within 0.05) with two cell types, indicating that the FC expression pattern on these cells is almost equally matched to two cell types. When this was the case, these cells were assigned a predicted label with both cell types in the format of the max predicted cell type followed by the second predicted cell type (for example neurons-NPC). These double labeled cells may represent an intermediated cell type, for example the merged lable of neurons and NPCs, which are the same cell type on a continuum of differentiation are likely to be early neurons (Figures S8 and S9). The CAM is a useful tool to provide biologists with a predicted cell type and guide annotation, however, it does not deliver the accuracy needed to quantify cell types across experiments. We therefore created tools to use CAM in combination with other methods. Clustering algorithms group together cells with similar expression profiles, thus cells that are not clearly identified as a given cell type in isolation can be identified based on their neighbors. We created functions to identify the topmost predicted cell types per cluster.

Using the functions in our CelltypeR library we performed unsupervised clustering using dimensional reduction by principal component analysis, generation of a neighborhood graph followed by Louvain network detection and visualized the protein expression levels in each cluster (Figure S10). Clusters were annotated with cell types using a combination of marker expression by cluster and the output from the correlation predicted cell types (Figure 3D). We identified astrocytes, radial glia, epithelial cells, endothelial cells, NPCs, neurons, a small proportion of oligodendrocytes and OPCs, and stem cell-like cells in the hMOs (Figure 3E). Clustering the hMO cells identified distinct subpopulations of radial glia, astrocytes, and neurons. All these cell types have a wide diversity in the brain and as well as in hMOs.^1^^,^^44^ For example, *neurons 1* and *2* could represent two major subtypes of mature neurons. We conclude that our workflow can be used to annotate cell types in hMOs and capture some diversity within cell types.

### Proportions of cell type composition differs between the different healthy iPSC line derived hMOs

**Figure 4:**
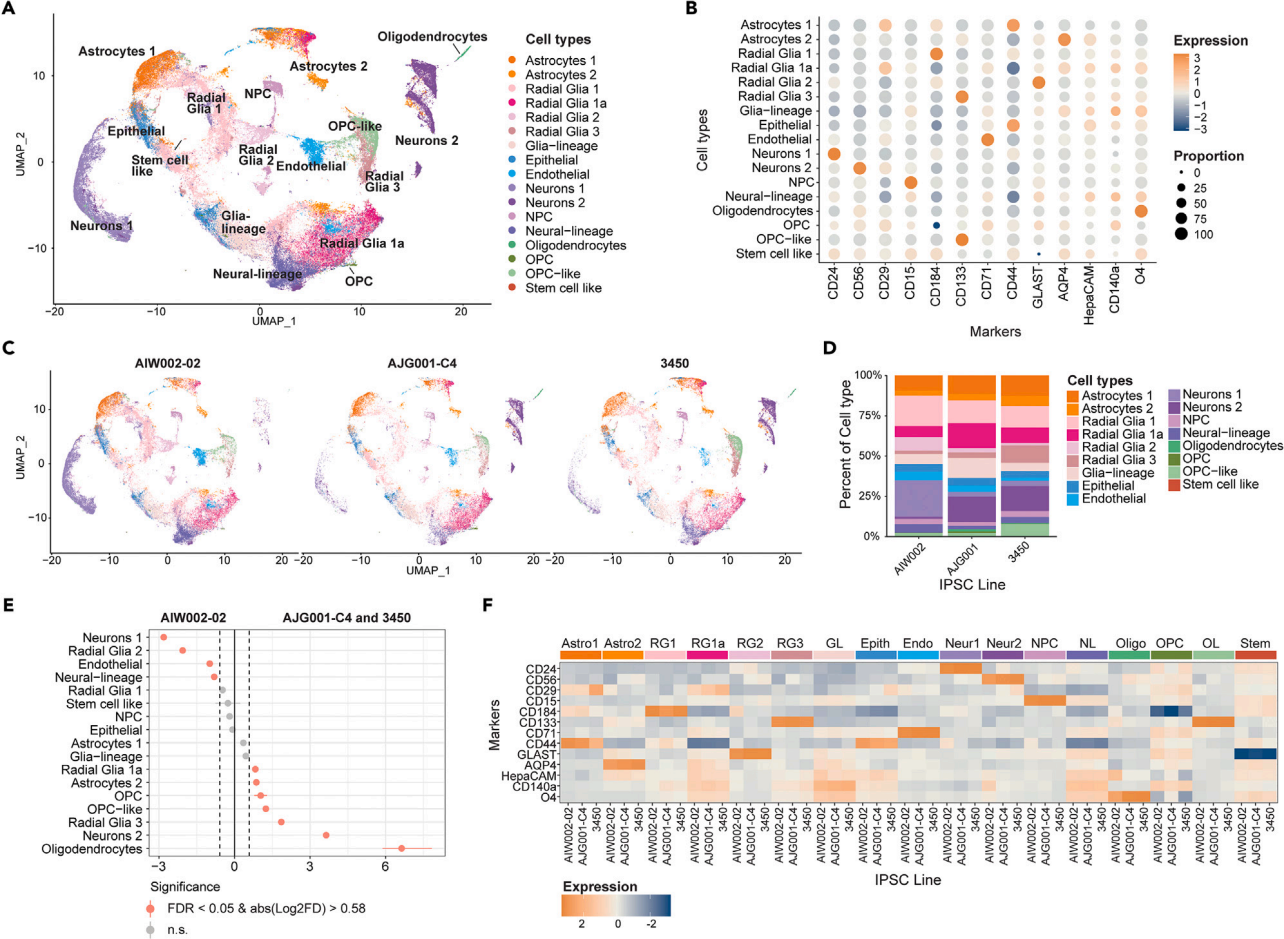
Differences in cell types and protein expression between three healthy control donor iPSCs (A) UMAP of the full dataset from 9 hMO samples, three genotypes (AIW002-02, 3450, and AJG001C) from two batches (A and B) and 2 experimental time points annotated using CelltypeR. (B) Dot plot of the expression level (color intensity) and the proportion of cells (dot size) for each protein marker detected with the panel in each cell type group. Scaled *Z* score values are shown. (C) UMAP split by iPSC line (3 samples pooled per iPSC line) showing the proportion of cells in each iPSC line. Cell annotations and colors are the same as the UMAP in A. (D) Bar chart of the proportion of hMO cells in each cell type (indicated by color) for each iPSC line (x axis). Colors corresponding to cell types are shown in the legend on the right (*n* = 3 replicates per line, combined). (E) Dot plot with confidence interval for the proportionality test comparing the AIW002-02 iPSC line to the AJG001-C4 and 3450 iPSC lines, for each cell type (y axis). Pink dots indicate a significant difference in cell type proportion (FDR <0.05 and absolute value of Log2FD > 0.58). Negative log2FD values indicate cell proportions increased in AIW002-02 and positive values indicate cell proportions decreased in AIW002-02 compared to the other two iPSC lines. (F) Heatmap of mean protein expression values grouped by cell type and split into the three iPSC lines. Line names are indicated on the bottom x axis and cell types are indicated on the top x axis. Scaled *Z* score values are shown. Total cells analyzed = 197160. Individual hMO counts can be seen in Table 1. See also Figures S11–S14 and Tables 2, S2, and S3.

After annotating a subset of 9000 cells from each of the nine hMO samples, we next analyzed the total available cells. We again followed the CelltypeR workflow and can now use the labeled subset of cells to annotate the full dataset. We first clustered the full dataset and then annotated the cells from the nine hMO samples (Figure 4A). Using CelltypeR functions, we trained a random forest classifier model (RFM) to predict cell types (Figure S11).^50^ In addition to analyzing protein expression profiles by cluster, we created functions for and used three prediction methods (CAM, RFM,^50^ Seurat label transfer^41^) to annotate cell types (Figures S12 and S13). We observe the same cell types in the full dataset as in the subset of data; however, we now identify one cell group predicted to be both OPCs and Radial Glia 1, which we termed *OPC-like* (Figure 4A). We examined the protein expression levels within our cell type annotations and distinctive expression profiles (Figure 4B).

Visualizing the distribution of cell types in hMOs derived from each cell line, we can see there are some differences in the proportion of cell types (Figures 4C and 4D). We observe more *neurons* *1* and fewer *neurons* *2* in the AIW002 hMOs compared to the other two lines. The AIW002 hMOs also have less *oligodendrocytes* than AJG001-C4 and 3450 hMOs. We used permutation tests to determine if the differences in proportions of cell types between the cell lines are significant. Permutation tests compare the observed proportions to the distribution of multiple iterations of randomly shuffling the samples without assuming cell types are independent and data is normally distributed.^51^ We created a permutation test for single cell data using the ANOVA permutation test from the *Permuco* R library to compare across all 3 cell lines.^52^ We find that only *oligodendrocytes* and *neurons 1* differ significantly across all lines (Figure S14). To compare between two conditions, we used the *scProportionTest* R library designed for single cell data.^53^ AIW002-02 compared to 3450 and AJG001C together shows significantly different proportion of cell types for *neurons*, *oligodendrocytes*, and subtypes of glia cells (Figure 4E). Comparing pairwise combinations between the three iPSC lines we also see differences across all lines. Notably AJG001-C4 hMOs have the most *oligodendrocytes* and *OPCs*, and fewer *NPCs* than the other two lines (Figure S14). To visualize if expression patterns differ within one cell type between iPSC lines, we plotted a heatmap of mean protein expression and observe most proteins have consistent expression across iPSC lines in most cell types (Figure 4F). To further explore expression differences between groups, we created functions in our R package to run ANOVAs, post-hoc tests, and identify significant differences. We performed two-way ANOVAs on each cell type, followed by Tukey’s post-hoc tests to compare expression levels for each marker protein across iPSC lines. There are significant differences in overall marker expression levels between the three different iPSC lines in *neurons*, *NPCs, oligodendrocytes,* and *OPC-like cells* (Table S2), *n* = 3 separate hMO samples for each iPSC line. Tukey’s post hoc tests show that only a few individual markers have significantly different expression between pairs of iPSC lines (Table S3). Using our framework, we can reliably quantify cell types and compare proportions of cells and levels of antibody expression across different conditions. We find significant differences in the proportion of cell types and in marker expression levels within cell types between different healthy control iPSC lines.

### The CelltypeR workflow can reliably assign cell types across different datasets

**Figure 5:**
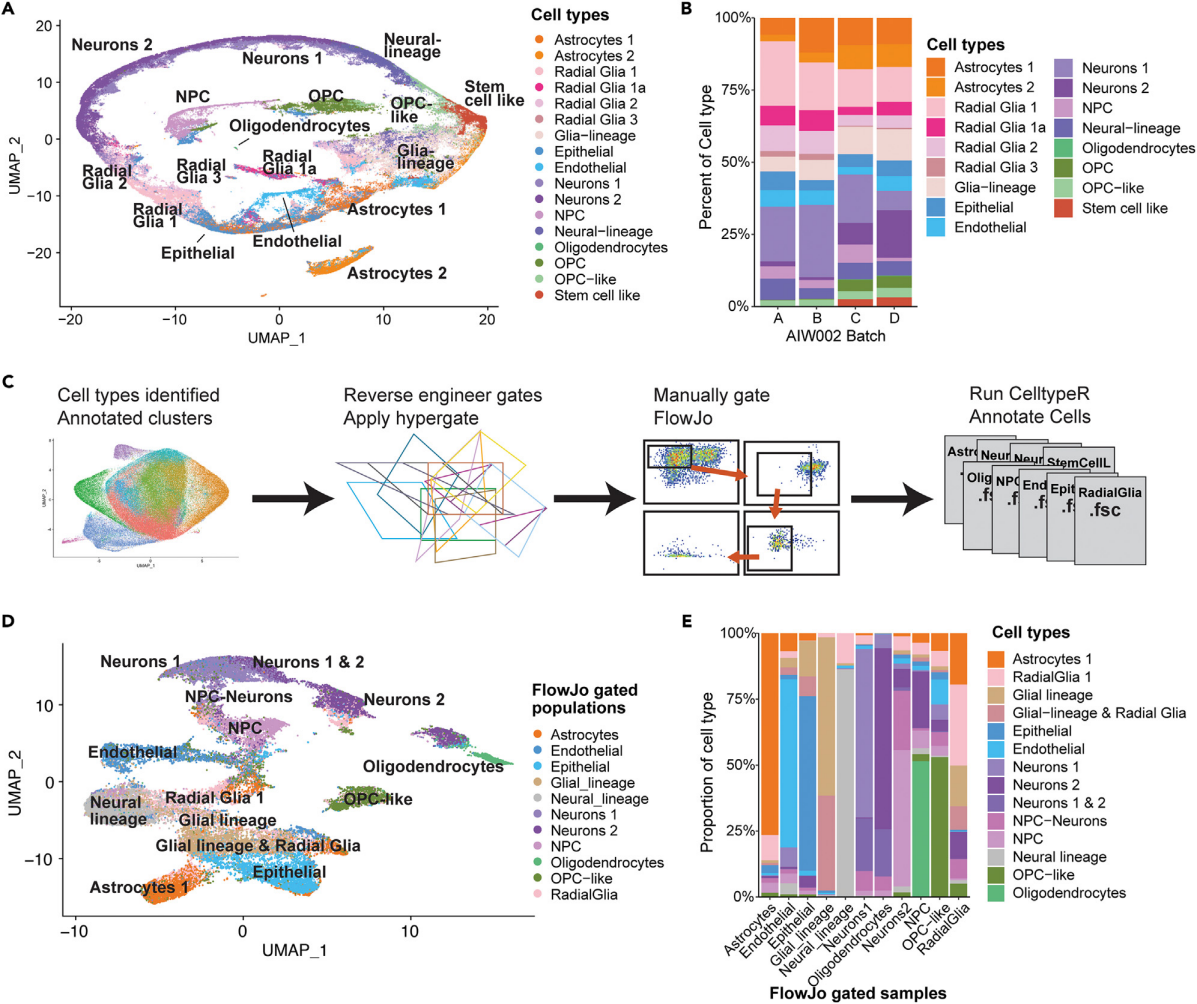
CelltypeR can be used to identify cell types in a new population, gate populations of interest and annotate the gated cells (A) Two new batches (C and D) of AIW002-02 hMOs were processed with the CelltypeR workflow and cell types were annotated. UMAP shows cells from seven samples dissociated from the two AIW002-02 batch acquired on two different days, total cells = 202389. (B) Bar chart showing the proportions of cell types across four different batches of AIW002-02 hMOs. Batches A and B are the samples from the 9 hMO comparisons, batches C and D are the new samples shown in panel A. (C) Schematic showing the method used to gate cell type populations defined with CelltypeR. Cell types were annotated and selected in the full 9 hMO dataset. Then the package *hypergate* was applied to reverse engineer the threshold expression levels to define each cell population. Gates were applied to the 9 hMO samples with two batches (A and B) and three iPSC lines (AIW002-02, 3450, and AJG001C). (D) UMAP colored by the populations (see legend) gated in FlowJo using the thresholds and markers selected by *hypergate.* Astrocytes, radial glia, oligodendrocytes, epithelia cells, endothelial cells, NPCs, Neurons 1, and Neurons 2 cell populations were exported as fsc files and input into the CelltypeR workflow. Gated cells were down sampled to 5000 cells, except for oligodendrocytes where all 1170 cells were included. The labels on the UMAP are the cell types annotated using the CelltypeR workflow. (E) Bar chart with the proportion of cell types identified with CelltypeR (indicated by color in the legend) within each FlowJo gated population (x axis). See also Figure S15; Tables S4 and S5.

We next generated new batches of hMOs using the control cell line AIW002-02 to validate the CelltypeR workflow on a new dataset. The antibody panel was applied, and intensity levels were measured by FC from two different batches on one experiment day, and on one of the batches on a second experiment day. The cell types in these new batches were processed as previously described above and in the STAR Methods. The cell types were annotated using CelltypeR functions. For RFM and Seurat label transfers, the cell type labels from Figure 3D were used as the reference data. The cell types in the new hMO samples were found to be consistent with the original AIW002-02 samples (Figure 5A). To determine if the proportion of cells was similar across the two new and two original AIW002-02 batches, we plotted the percentage of each cell type grouped by hMO batch and observe similar but varying proportions of cell types across batches (Figure 5B). Batches A and B are from the original dataset and were grown with a different protocol than the two new batches C and D (see STAR Methods). We next performed a permutation test across all batches and find no significant differences (Figure S15A). Using pairwise permutation tests, there were more differences in cell type proportions between batches A,B and C,D than between the batches grown with the same protocol (Figure S15B). The relative proportion of several radial glia populations are increased in batches A and B compared to batches C and D. Whereas *Neurons 2*, *OPCs*, *oligodendrocytes* and *stem cell like* populations are all relatively decreased in batches A and B compared to batches C and D (Figures 5B and S15). We concluded that CelltypeR can identify cell types across datasets separately processed and compare between batches.

### Populations of interest, identified by CelltypeR clustering analysis, can be enriched and purified by FACS

After annotating a dataset, we could plot the proportion and mean expression of every antibody marker in each group to try to define the relative marker expression of a given cell group and then isolate that population by FACS. However, manually reverse engineering a gating strategy is difficult with more than a few cell type markers. Thus, we defined cell types using CelltypeR, applied the package *hypergate*^54^ to identify which combinations of antibody markers clearly define a given cell population, and then manually gated these cells in FlowJo (Figure 5C). To reduce the number of potential populations to gate, subgroups of the radial glia cells and astrocytes were merged. *Hypergate* was applied to define the threshold for each antibody relevant for gating. The resulting gating accuracy for all cell types is above 95% (Table S4). We next used the defined gates in FlowJo to gate the cell type populations (Figure 5D). Although the *Hypergate* defined gates are accurate when tested in R, when applied in FlowJo, OPCs and stem cells were not accurately selected, with far more than the expected cell numbers passing the gates (Table S5). The gates are defined by upper and lower threshold for markers important for each cell type (Table S4). All the cell type populations accept the *OPCs* and *stem cells* are accurately selected in FlowJo and have at least one marker with higher expression than the other cell types, creating a positive threshold for gating. The lack of a positive marker threshold to define the *OPC* and *stem cell like* populations is likely leading to the poor FlowJo gating of these populations. Therefore, we excluded the *OPC* and *stem cell* populations analysis in the next steps. The remaining gated populations were entered into the CelltypeR workflow and clusters of cells were annotated (Figure 5D). Some clusters clearly contained two cell types, and these were labeled as such to reflect the mixtures. The proportion of CelltypeR cell types within each FlowJo gated population was calculated (Figure 5E). The most frequent annotated cell type within each gated population is the intended cell type. Merging the two astrocyte populations, resulted in gating the *astrocytes 1* population and not enough of *astrocytes 2* to be detected. The merged radial glial populations resulted in a less effective gate that only selected *radial glia 1* and *radial glia 3* populations. We concluded that our workflow can be used to isolate selected populations with relatively higher expression of at least one marker.

### ScRNA-seq transcriptomes validate CelltypeR-based cell type assignments

**Figure 6:**
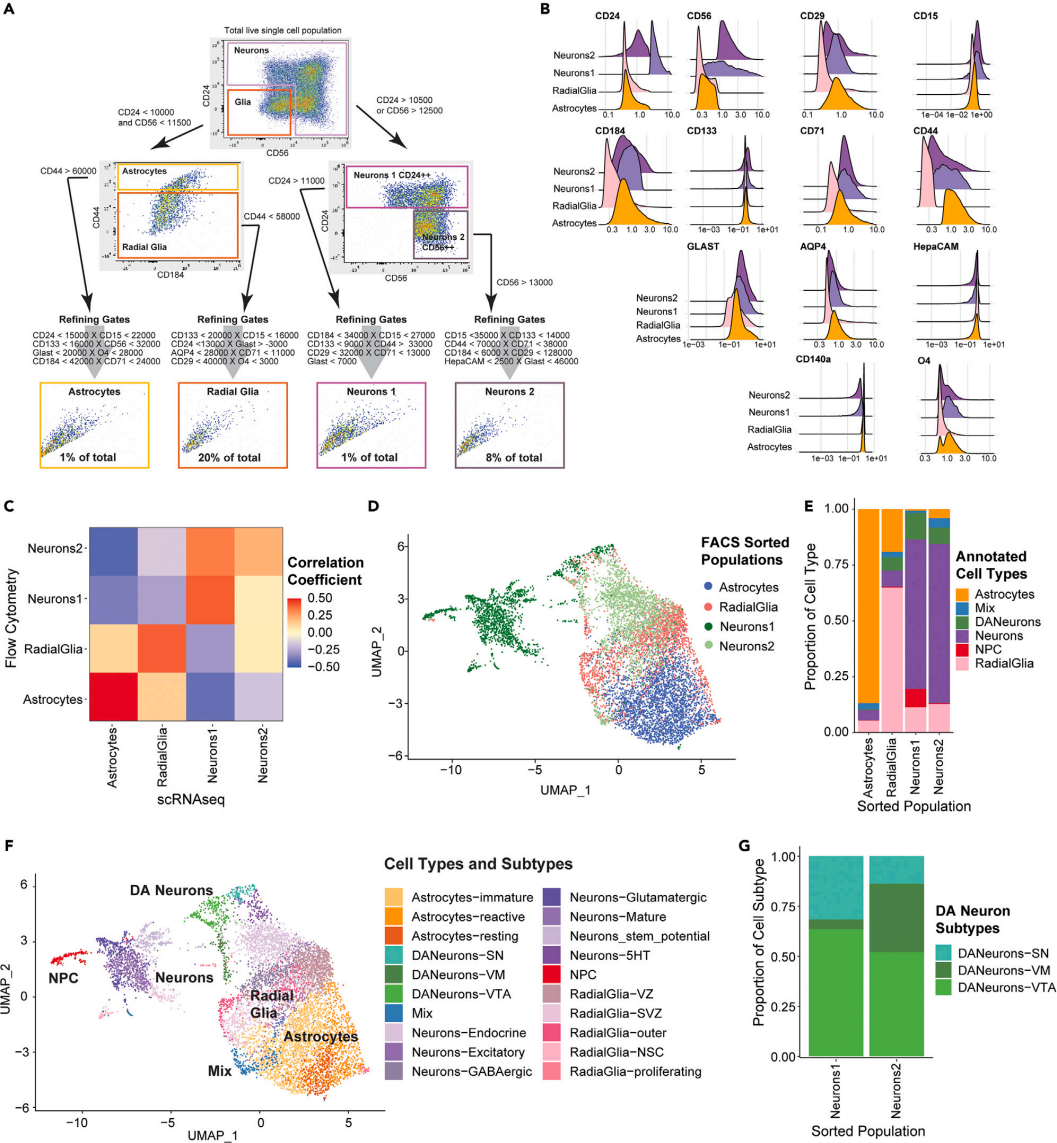
scRNA-seq analysis of four FC sorted populations defined using CelltypeR confirms cell types and provides transcriptional profiles for these cell populations (A) FlowJo gating strategy applied to new hMO derived cells to isolate four cell populations by FACS: Neurons 1, Neurons 2, astrocytes, and radial glia. The approximate proportion of cells gated in each final sorted population is indicated in the gating box. (B) Ridge plot of protein expression levels measured by FC antibody intensity for each FACS gated cell population. (C) Correlation of RNA transcript expression of genes corresponding to the 13 protein markers used for FACS. There is a statistically significant correlation between RNA expression and protein expression in the astrocytes. The Neurons2 protein expression correlates more strongly with the Neurons1 RNA expression. (D) UMAP of scRNA-seq transcriptomes of the four sorted populations merged and clustered with Louvain network detection. Neurons1 has only 1723 cells, Neurons 2 was down sampled to 2000, astrocytes were down sampled to 3000, and radial glia were down sampled to 2000 to improve visualization. The original FACS population is indicated by color in the legend. (E) Stacked bar chart of the proportion of each main cell type identified by the cluster transcriptomes in each FACS sorted population. (F) UMAP of the four merged populations with cell types and cell subtypes annotated from the scRNA-seq data. The UMAP is colored by cell subtypes and the main cell types are labeled on top of the UMAP. (G) Stacked bar chart showing the proportion of each DA neuron subtype within each sorted neuron population. See also Figures S16–S22; Tables S6–S10.

Our workflow can be used to enrich populations of interest by FACS, separating selected populations for further analysis. We selected four cell types that were gated well in FlowJo and were of interest for further study: *Neurons 1*, *Neurons 2*, *Astrocytes*, and *Radial* *Glia*. We next designed a gating strategy to simultaneously sort the four populations (Figure 6A). We sorted the hMO-derived cells using the defined gates, split the samples, and then acquired FC measurements and scRNA-seq on the sorted populations. Analysis of the protein expression levels in the sorted populations confirmed the populations were gated effectively (Figure 6B). The post sorting cell viability was greater than 85% (Table S15). We also obtained a single cell transcriptomic library for each of the FACS populations (see STAR Methods). We first compared the RNA expression levels of the genes corresponding with the protein expression levels measured by FC and found they positively correlate (Figures 6C and S16; Table S6). The scRNA-seq libraries from the four populations were merged, clustered, and plotted on a UMAP to visualize the overlap between the different sorted cell types (Figure 6D). The *Neurons 1* population is mostly separate from the other populations with some overlap with *Neurons 2*. Clusters were first annotated for main groups of cell types: DA neurons, neurons, NPCs, radial glia, and astrocytes and the proportion of these cell types in each sorted population was calculated (Figure 6E). Cell types were annotated with a combination of comparing markers identified by differential gene expression (DGE) between clusters with cell type reference libraries,^55^ label transfer from multiple brain^44^^,^^45^^,^^47^^,^^48^ and organoid^1^^,^^49^ datasets (see STAR Methods) and expression scores for known sets of markers.^56^ These main cell types were then isolated and annotated for subtypes of cells using DGE between clusters (Figures 6F and S17–S20; Table S7). To annotate the three subgroups of DA neurons we used cluster markers and compared expression with published markers (Figure S18; Tables S8 and S9). Next, we examined the proportion of cellular subtypes in the sorted populations (Figure S21; Table S10). We found that non-DA neurons in *Neurons1* are excitatory neurons, neurons with potential to be reactivated as neural stem cells, NPCs, and ventral zone (VZ) radial glia undergoing neurogenesis (Figure S17). The non-DA neurons in the *Neurons2* population are GABAergic, serotonergic (5HT), and endocrine neurons (Figure S17). As quantification of DA neurons is of particular interest in hMOs for PD, we calculated the proportion of all the DA neurons and the three subtypes of DA neurons in the sorted populations. We find that *Neurons1*, *Neurons2*, and *Radial Glia* all contain DA neurons (Figure S22; Table S10). The *Neurons1* FACS population has slightly more DA neurons overall, and specifically more of the substantia nigra (SN) subtype, whereas the Neurons2 FACS population has more cells from the cluster identified as general ventral midbrain (VM) DA neurons. The subgroup containing ventral tegmental area (VTA) DA neurons didn’t show a significant differential distribution between the *Neurons1* and *Neurons2* FACS groups (Figures 6G and S22). Thus, the two sorted neuron populations contain distinctive subtypes of DA and non-DA neurons. Moreover, the astrocyte population also segregated into three subgroups, immature, resting, and reactive (Figures S19 and S21). The *radial glia* population contains five different subtypes (Figures S18 and S21). Taken together, our findings indicate that each FACS population is enriched in the expected cell type and there are identifiable subtypes within these groups, confirming the effectiveness of the CelltypeR framework.

### CelltypeR workflow identifies changes in hMO cell type proportions over time

**Figure 7:**
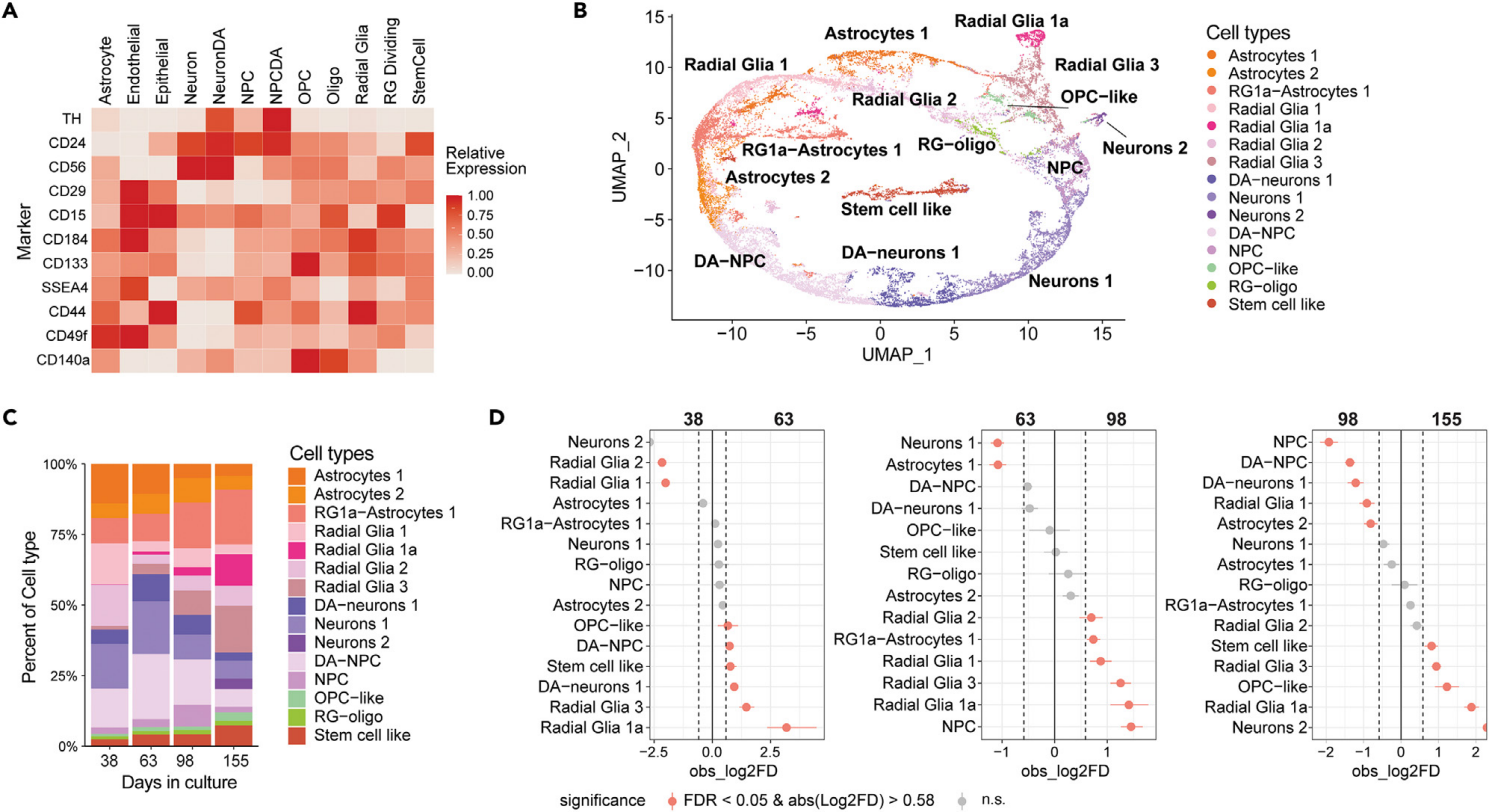
Cell type proportions in hMOs change over time in culture (A) Heatmap of predicted relative expression of each protein targeted in the new FC panel for each potential cell type in hMOs. Values are calculated from 2D FC intensities, scRNA-seq from hMOs and human brain, and RNA-seq from human brain. (B) UMAP of cells from the line AIW002-02 (batch E) with four experimental replicates per time point annotated using the CelltypeR workflow. Cells were down sampled to 2000 cells per sample, *n* = 32000. (C) Bar chart of the proportions of cell types for each time point, 4 replicates were combined. (D) Proportionality tests comparing time points in pairs, from left to right: 30 days vs. 60 days, 60 days vs. 100 days and 100 days vs. 150 days. Differences that have a change in proportion >0.58 logFold change and an adjusted *p* value <0.05 are shown in pink. See also Figure S23; Table S11.

To test the ability to adjust and refine the antibody panel we decided to change our antibody panel and apply the pipeline to a time course of hMOs in one genetic background. To directly track DA neurons we added TH, the only protein with a cytoplasmic epitope to the panel. Adding a cytoplasmic marker does slightly increase the experiment time, but it is still feasible for multiple samples (see STAR Methods). We made other alterations to the panel to display the flexibility of our CelltypeR pipeline. We added “SSEA4”, a pluripotency marker^57^ and “CD49f”,^58^ reported to be a marker of activated astrocytes^58^ and removed CD71, AQP4 and GLAST, HepaCam and O4 (Table S11). A new reference matrix was created for the adapted marker panel, now including DA neurons and DA NPCs (Figure 7A). We acquired FC measure of protein expression across four time points in one batch of AIW002-02 hMOs and followed the CelltypeR workflow. We noted that the percentage of live cells after dissociation declines with time in culture (Table S14). We used the combination of CAM prediction with the new reference matrix, RFM and Seurat label transfer predictions and expression analysis to annotate clusters (Figure 7B). Next, we plotted the proportion of cell types at each time point and found that cell types are changing over time in the hMOs and show less variation between replicates (Figures 7C and S23). We observe that the proportions of both *DA neurons*, *neurons* and *DA-NPCs* increase from 38 to 63 days, then decrease from 98 to 155 days. *Stem cell like* and glial populations show an increase over time. To test if the changes in the proportions of cell types are significant, permutation tests were performed. There are significantly more *DA-neurons* at 63 than 38 days, and at 98 than 155 days, but no difference is seen between 63 and 98 days (Figure 7D). An ANOVA permutation test across all time points showed all the cell types change significantly with time (Figure S23). The changes in cell type proportions match with the expected time course of differentiation, as stem cell and early glial cells will continue to divide where-as neurons and mature astrocytes do not divide. We conclude that the CelltypeR workflow is versatile as it can be applied with different antibody panels, and it can be used for detecting differences in cell type populations over time.

## Discussion

Taken together, we present the first comprehensive antibody-based workflow to identify, quantify, compare, and sort cell types in complex 3D tissue, specifically hMOs. We define a 13-antibody panel that can be used to distinguish between eight different brain cell types and identify subtypes of astrocytes, radial glia, and neurons. The antibody panel is modular, such that it can be altered or expanded for any organoid or tissue type and will function with the computational workflow. We show an example of this by generating an altered antibody panel using the cytosolic TH antibody to detect DA NPCs and neurons. To apply the method to a completely different tissue, for example gut organoids, an antibody based on and reference matrix from published data would need to be designed. In our CelltypeR library, we provide a method to preprocess and merge FC samples, acquired from multiple samples at different dates. We created tools to optimize and visualize clustering and to assist in consistent cell type annotation. We also created functions to quantify cell types and compare different conditions. Our computational workflow can be applied to analyze single cell data from any tissue.

The same workflow with sorting can be used to isolate a more homogeneous subpopulation of a given cell type for use in other assays that includes but is not limited to proteomics, lipidomics, testing small molecules, or even replating of cells in culture to grow as a purified population. Here we selected four populations, FACS sorted the cells, and then performed scRNA-seq analysis. We confirmed that each of the populations, *Neurons1*, *Neurons2*, *Radial Glia* and *Astrocytes*, are all highly enriched in the expected cell types. Further analysis of the scRNA-seq data identified subtypes within each cell type group. We identified DA neurons within both neuronal populations but find different DA neuron subtypes enriched in each of the two FACS sorted neuron populations. As an example, we identified TPGB as a DA subtype marker (ventral), in agreement with a recent publication proposing TPGB as a marker of ventral DA neurons in mice.^59^ The DA neurons of the SN are selectively lost in PD, whereas the VTA neurons are important for studying addiction and the reward system. We see an enrichment of SN-like neurons in one of our sorted neuron populations. Isolating specific subtypes of DA neurons is fundamental for research targeting specific disease pathways, like those leading to SN DA neuron cell death in PD. We also identified VTA DA neurons which were not specifically enriched in either sorted neuron population. The third group of DA neurons were identified as ventral midbrain DA neurons but were not specific for SN or VTA.

Using an adjusted antibody panel, we applied the CelltypeR workflow to track cell type proportions over time in AIW002-02 hMOs. We found that the proportion of DA-NPCs, neurons 1, and DA neurons all follow a similar pattern, increasing from 38 days to 63 days and then decrease at later time points. Neurons 2 only appear at the 155-day time point, indicating this group could be more mature. The neural populations within the hMOs exhibited intriguing temporal dynamics. It’s important to note that this decrease in relative neuronal proportion does not necessarily signify a reduction in the total number of neurons but rather suggests changes in their proportions within the overall cell population. Additionally, we observed relative increases in radial glia 1a, radial glia 3, stem cell like cells, and OPC-like cell populations. These cell populations are likely proliferating and represent partially differentiated cells, which possess the potential to differentiate into astrocytes, neurons, or other brain cells over time.

In our analysis of the differences between three healthy control iPSC lines, we find a clear difference in the proportion of cells for the two subtypes of neurons between AIW002-02 compared to the other two lines, AJG001 and 3450. AIW002-02 has more *Neurons1* with higher CD24 expression and fewer of the *Neurons2* population, with lower CD24 expression than AJG001 and 3450. scRNA-seq reveals the *Neurons1* population has more NPCs and DA neurons. We also find that AIW002-02 has more radial glia, fewer astrocytes, and fewer oligodendrocytes than the other two lines, indicating that this cell line may be less mature. AIW002-02 might mature at a slower rate or given the late age of the hMOs, maintain cell populations with potential to become precursors perpetually. These findings also indicate that to study the role of myelination, the AJG001 or 3450 lines could be a better choice than AIW002-02.

In conclusion, we established an adaptable method for reproducibly identifying and quantifying cell types in complex 3D tissues, such as hMOs, using an FC panel. Our scalable single-cell biology workflow enables rapid and efficient cell type quantification across multiple replicates and experimental conditions. Our method costs about 35X less per sample than scRNA-seq. Quantifying nine cell type from tissue sections is not feasible; parallel cryosections with labeled with multiple antibodies would need to be imaged with confocal microscopy and counted, taking hours of time in imaging and analysis for each marker set and sample to only quantify a small proportion of the tissue sample. Here, using CelltypeR, we have highlighted the potential of our workflow in the context of tracking cell type dynamics over time, comparative analysis of iPSC lines, and its adaptability for diverse tissue types. Furthermore, we emphasize the importance of creating reference matrices for changes in the antibody panel, thereby enhancing the utility and applicability of the CelltypeR workflow in various research domains.

### Limitations of the study

The FC method we have presented requires fresh tissue and the number of samples is limited by the amount that can be prepared at once. While fresh tissue is readily available from cultured cells and tissues that can be processed immediately after collection, it is not feasible for many complex tissues, such as tumors or postmortem samples. Other methods, such as histology do not require fresh tissue. An adapted protocol to prepare samples from frozen or fixed tissue would be beneficial. However, after antibody labeling the cells can be fixed and kept at 4°C and protein levels can be acquired for up to 2 weeks. This allows samples prepared on different days to be measured on the same day. The throughput of the samples is limited by the preparation. The number of cells, samples, and markers is completely scalable within the computational framework. However, more computational power is required as the number of cells is increased.

The workflow is limited by the number of antibodies possible to combine in the flow cytometer. The technology for spectral analyzers is always advancing, currently some devices can utilize 40 channels. Conveniently, the CelltypeR computational workflow can scale to any number of antibodies and will work for FC data acquired from any type of sample providing fsc files are generated. The larger the number of antibodies in the panel the more cell types that could be identified with higher confidence, although the cost will increase with additional antibodies. In our work we were not able to define effective gates for OPCs and stem cell–like cells to potentially isolate these cell types because none of our markers were distinctly upregulated in these cell types. While we can identity these cell types, to sort cells a positive marker is required.

## STAR★Methods

### Key resources table

**Table:** 

REAGENT or RESOURCE	SOURCE	IDENTIFIER
**Antibodies**		

MAP2	EnCor Biotech	Cat# CPCA-MAP2;RRID:AB_2138173
Nestin	Abcam	Cat#ab92391;RRID:AB_10561437
SSEA-4	Santa Cruz Biotechnology	Cat#sc-2170
GFAP	Dako	Cat#Z0334
AQP4	Sigma	Cat#HPA014784;RRID:AB_18449676
O4	R&D	Cat#MAB1326;RRID:AB_357617
PDGFRa	Cell Signaling	Cat#3174;RRID:AB_2162345
Tyrosine-Hydroxylase (TH)	Pel-Freez	Cat#P40101;RRID:AB_2313713
Neurofilament (NF)	Sigma	Cat#N5264
GIRK2	NovusBio	Cat#NB100-74575
FOXA2	abcam	Cat#ab117542
S100b	Sigma	Cat#S2532
CD44	Biolegend	Cat#338810
Aquaporin-4 (AQP4)	Bioss	Cat# bs-0634R-A488
GLAST	Miltenyi Biotec	Cat#130-095-814
HepaCAM	Bioss	Cat# bs-5840R-A594
CD71	Biolegend	Cat#334116
CD184	Optibuild	Cat#740926
CD133	Biolegend	Cat#372810;RRID:AB_2686968
CD15	Biolegend	Cat#323044;RRID:AB_2632921
CD29	Biolegend	Cat#303014;RRID:AB_493580
CD56	Biolegend	Cat#392420;RRID:AB2734444
CD24	Biolegend	Cat#311136
O4	Miltenyi Biotec	Cat#130-117-357;RRID:AB_2733887
SSEA-4	Biolegend	Cat#330418;RRID:AB_2616819
CD49f	Biolegend	Cat#313626;RRID:AB_2616782
Tyrosine Hydroxylase (TH) – Coupled to PE	Invitrogen	Cat#MA5-38641

**Chemicals, peptides, and recombinant proteins**		

Matrigel	Corning Millipore	Cat#354277
Poly-L-ornithine (PLO)	Sigma-Aldrich	Cat#P3655
Laminin	Sigma-Aldrich	Cat#L2020
mTeSR1	StemCell Technologies	Cat#85850
E8	ThermoFisher Scientific	Cat#A1517001
DMEM/F12	Gibco	Cat#10565–018
Neurobasal medium	Life Technologies	Cat#21103–049
N2	Life Technologies	Cat#17502–048
B27	Life Technologies	Cat#17504–044
GlutaMAX	Gibco	Cat#35050-061
Antibiotic-antimycotic	Gibco	Cat#15240–062
Ascorbic acid	Sigma-Aldrich	Cat#A5960
SB431542	Selleckchem	Cat#S1067
CHIR99021	Selleckchem	Cat#S2924
Noggin	Peprotech	Cat#120-10C
MEM-NEAA	Thermo Fisher	Cat#11140050
2-mercaptoethanol	Merck	Cat#8057400005
Heparin	Sigma-Alderich	Cat#H3149
SHH	Peprotech	Cat#100-45
FGF8	Peprotech	Cat#100-25
Pen/Strep	Wisent	Cat#450-200-EL
ROCK inhibitor	Selleckchem	Cat#S1049
Hoechst 33342 DNA dye	Life Technologies	Cat#H3570
Brain-derived neurotrophic factor (BDNF)	Peprotech	Cat#450–02
PFA	Thermo Fisher	Cat#28908
PBS	Wisent	Cat# 311-013 CL
Dulbecco’s PBS	Wisent	Cat#311-425 LL
Bovine serum albumin (BSA)	Multicell	Cat#800–095-CG
Triton	BioShop Canada	TRX777.500
Normal donkey serum (NDS)	Millipore	Cat#S30-100
Fluoromount-G	Invitrogen	00-4958-02
Optimal Cutting Temperature Compound (OTC)	Fisher Healthcare	Cat#23-730-571
Normal Goat Serum	Jackson Immunoresearch Laboratories	Cat# NC9660079
Aqua-Poly/Mount mounting medium	Polysciences	Cat#18606
TrypLE express (without phenol red)	ThemoFisher	Cat#12604013
TruStain FcX	Biolegend	Cat#422302
NaN_3_	Sigma	S2002-500G
Tween-20	Bioshop	TWN510.500

**Critical commercial assays**		

UltraComp eBeads™ Plus Compensation Beads and ArC™ Amine Reactive Compensation Bead Kit	ThemoFisher	Cat# A10346
Lightning-Link PE	Abcam	Cat# ab102918
Chromium Next GEM Chip G Single Cell Kit	10X Genomics	Cat#1000120
Chromium Next GEM Single Cell 3’ reagent kit v3.1	10X Genomics	Cat#1000268
Dual Index Kit TT Set A	10X Genomics	Cat#1000215
Dual Index Kit NT Set A	10X Genomics	Cat#1000242
Dynabeads MyOne SILANE	10X Genomics	Cat#2000048

**Deposited data**		

scRNAseq of 4 sorted cell population from hMOs	This study	GSE226890
Live gated flow cytometry data	This study	https://github.com/RhalenaThomas/CelltypeR/tree/main/FlowCytometry_Data
Raw Flow Cytometry data	This study	https://data.mendeley.com/preview/sxj55468hm
scRNAseq from human developing cortex	Nowakowski et al., 2021^60^	https://cortex-dev.cells.ucsc.edu
scRNAseq from human fetal midbrain	La Manno et al., 2016^46^	GSE76381
scRNAseq from human developing forebrain	Van Bruggen et al. 2022^45^	forebraindev.cells.ucsc.edu
scRNAseq from human developing midbrain and striatum	Bhaduri et al. 2021^60^	https://dev-brain-regions.cells.ucsc.edu
snRNAseq from human adult midbrain	Kamath et al. 2022^48^	https://singlecell.broadinstitute.org/single_cell/study/SCP1768/
scRNAseq from hMOs	Mohamed et al. 2021^1^	GSE186780
scRNAseq from cerebral organoids	Tanaka et al. 2020^49^	https://cells.ucsc.edu/?ds=organoidatlas&meta=Cluster

**Experimental models: Cell lines**		

AJG001-C4	Chen et al. 2021^1^	https://www.neuro-edduportal.com/ipsc-catalogue
AIW002-02	Chen et al. 2021^1^	https://www.neuro-edduportal.com/ipsc-catalogue
3450	Chen et al. 2021^1^	https://www.neuro-edduportal.com/ipsc-catalogue

**Software and algorithms**		

FlowJo	BD Bioscience	https://www.flowjo.com/solutions/flowjo
R programing	Open Source	https://www.r-project.org/
FC data input and formatting in R *fsc_to_fs, flowset_to_csv, make_seu*	This study	https://github.com/RhalenaThomas/CelltypeR/
Alignment of FC expression data *harmonize*	This study	https://github.com/RhalenaThomas/CelltypeR/
Optimize FC data clustering *explore_param, clust_stability, get_clusters*	This study	https://github.com/RhalenaThomas/CelltypeR/
CAM to predict cell types using correlation to a reference matrix *find_correlations*	This study	https://github.com/RhalenaThomas/CelltypeR/
RFM to predict cell type assignments *RFM_train, RFM_predict*	This study	https://github.com/RhalenaThomas/CelltypeR/
Summarize predictions and annotate cells *get_annotate, annotate_df, annotate*	This study	https://github.com/RhalenaThomas/CelltypeR/
Statistics to compare expression *Prep_for_stats, run_stats*	This study	https://github.com/RhalenaThomas/CelltypeR/
ANOVA permutation test *permutation_test_multi*	This study	https://github.com/RhalenaThomas/CelltypeR/
Two conditions permutation test *permutation_test*	Miller et al. 2021^53^	scProportionTest
Seurat R library for single cell analysis	Stuart et al. 2019^41^	Seurat

**Other**		

Evos FL-Auto2 imaging system	ThermoFisher Scientific	Cat#AMF7000
Cryostat Cryostar NX70	Thermo Scientific	Cat#957000
gentleMACS M-Tube	Miltenyi Biotec	Cat#130-093-236
FACSAria Fusion	BD Bioscience	Cat# 656700G5
Bio-Rad C1000 Touch thermal cycler	Bio-Rad	Cat#1851196
NovaSeq6000	Illumina	Cat# 20012850
10X Chromium Controller	10X Genetics	Cat#PN-1000204

### Resource availability

#### Lead contact

Requests for additional information should be directed to Rhalena A. Thomas, PhD rhalena.thomas@mcgill.ca.

#### Materials availability

This study did not generate new unique reagents. Cell lines utilized in this study will be made available on request, under the open science framework of the Neuro, and through a cost recovery model. All data generated in this study are publicly available.

#### Data and code availability

• Raw Flow Cytometry data files are available in Mendeley Data. The cleaned live cell and gated data is available on Github https://github.com/RhalenaThomas/CelltypeR/FlowCytometry_Data.
• scRNAseq data from FC sorted populations available on GEO. The accession number is listed in the key resources table.
• All code for the CelltypeR library, workbooks to generate figures and package usage can be found on Github https://github.com/RhalenaThomas/CelltypeR/.

### Experimental model and study participant details

#### iPSC lines and hMO cultures

Three iPSC cell lines were used: AJG001-C4, AIW002-02-02, and 3450. All were previously reprogrammed from peripheral blood mononuclear cells and subjected to quality control measures.^25^ The AIW002-02 and AJG001-C4 iPSCs were expanded and maintained in mTeSR1 media and the 3450 lines was grown in E8 all lines were maintained on Matrigel coated plates. All work with human iPSCs was approved by McGill University Faculty of Medicine and Health Sciences Institutional Review Board (IRB Internal Study Number: A03-M19-22A).^25^ hMOs were derived from iPSC lines using two different protocols for method of embryonic body (EB) formation and long term culturing, the chemical agents and timing is identical.^4^^,^^61^ For both protocols iPSC are seeded for EB formation in neural induction media (DMEM/F-12 + GlutaMAX + Antibioic-Antimycotic, Neurobasal, MEM-NEAA, N2, B27 without vitamin A, 2-mercaptoethanl, Heparin, SB431542, Noggin, CHIR99021, ROCK inhibitor) and changed to midbrain patterning media (DMEM/F-12 + GlutaMAX + Antibioic-Antimycotic, Neurobasal, MEM-NEAA, N2, B27 without vitamin A, 2-mercaptoethanl, Heparin, SB431542, Noggin, CHIR99021, SHH, FGF8) after 4 days and then incubated in Matrigel for one day before being changed to tissue induction medium (Neurobasal, N2, B27 without vitamin A, GlutaMAX, MEM-NEAA, 2-mercaptoethanol, insulin, laminin, SHH, FGF8, Pen/Strep). After 24 hours the hMOs are transferred to long term cultures in final hMO growth medium (Neurobasal, N2, B27 without vitamin A, GlutaMAX, MEM-NEAA, 2-mercaptoethanol, BDNF, GDNF, ascorbic acid, db-cAMP, Pen/Strep). For batches A and B (Table 2) iPSCs were seeded in separate ultra-low attachment plates (EBs) to form and transferred to 6-well plates with 4-6 hMOs per cell line in organoid growth media and placed in shaking cultures.^4^ hMOs were maintained in shaking cultures with media change every 2-3 days. For batches C, D, and E, iPSCs were seeded in eNuvio disks for EB formation and Matrigel embedding, then transferred to bioreactors for culture maintenance.^61^ Media changes were performed weekly.

#### Cell culturing conditions for 2D cultures

##### DA-NPC and neurons DA neurons

DA-NPC cultures were generated by dissociating iPSCs into single cell suspensions and then culturing these cells in low attachment plates to generate EBs. EBs were re-plated onto polyornithine and laminin-coated plates and differentiated into neural rosettes, which were then differentiated into DA-NPCs. DA neurons were differentiated from DA-NPC cultures on laminin coated culture flasks in neural basal media with supplements and inhibitors as described.^38^

##### OPCs and oligodendrocytes

To derive OPCs and oligodendrocytes we used a three-phase protocol as previously described.^36^ In phase one, iPSCs were induced towards neural progenitors while being patterned with Retinoic Acid in order to resemble spinal cord progenitors. The Sonic Hedgehog pathway was activated for ventral patterning to recapitulate the conditions of the oligodendrocyte fate. The progenitors were subsequently expanded as EBs with the addition of the bFGF. In phase two, OPCs were expanded in suspension and subsequently plated onto polyornithine/laminin-coated vessels for adhesion. Growth factors and mitogens were added in the medium for differentiation and maintenance of the OPCs, respectively. Images of PDGFRα positive cells were acquired at this phase. In phase three, mitogens are withdrawn to allow the progenitors to exit the cell cycle and to complete differentiation into oligodendrocytes. Imaging and FC were performed in this phase when oligodendrocytes would generate O4 positive cells.

##### Astrocytes

Astrocytes were derived from NPCs cultures seeded at low cell density and grown in NPC expansion medium.^39^ The next day, medium was replaced with ‘Astrocyte Differentiation Medium 1’. Cells were split 1:4 every week and were maintained under these culture conditions for 30 days. At DIV50, cultures were switched to ‘Astrocyte Differentiation Medium 2’ and maintained with half medium changes every 3-4 days.

#### Immunofluorescence

For iPSC, NPCs and DA Neurons cells were fixed in 4% PFA/PBS at RT for 20 minutes, permeabilized with 0.2% Triton X-100/PBS for 10 min at room temperature (RT), and then blocked in 5% donkey serum, 1% BSA and 0.05% Triton X-100/ PBS for 2h. Cells were incubated with primary antibodies: MAP2 (1:1000); Nestin (1:500); SSEA-4 (1:200); in blocking buffer overnight at 4°C. Secondary antibodies were applied for 2h at RT, followed by Hoechst 33342 (1/5,000) nucleic acid counterstain for 5 minutes. Immunocytochemistry images were acquired using Evos FL-Auto2 imaging system.

For astrocytes cells were fixed for 15 minutes at room temperature with 4% formaldehyde in PBS, followed by 3 washes of 5 minutes in PBS. Cells were permeabilized for 10 min at RT in blocking solution: 5% normal donkey serum, 0.1% Triton-X-100, and 0.5 mg/ml bovine serum albumin in PBS. Cells were incubated for 1h at RT before overnight incubation at 4°C with primary antibodies: Glial Fibrillary Acidic Protein (GFAP) (1/50); AQP4 (1/500). Secondary antibodies were incubated 2h at, followed by Hoechst 33258 (1/5,000) for 5 min, mounted with Fluoromount-G, and examined by fluorescence microscopy.

For oligodendrocytes and OPCs cells were fixed in 2% PFA for 10 min and blocked in 5% BSA, 0.05% Triton for 1h. Mouse anti-O4 (1/1000) was added in live cells before fixation for 1hour. Rabbit anti-PDGFRa (1/200) was added post-fixation at and incubated overnight at 4°C. Secondary antibodies were added at a dilution of 1:500 and incubated for 2h at RT. Nuclei were identified with incubation with Hoechst 33342 (1/5,000) for 5 min.

HMOs were washed in PBS, and then fixed for 2h in 4% PFA diluted in PBS at RT, then placed in a sucrose gradient overnight at 4°C. hMOs were then embedded in Optimal Cutting Temperature Compound (OTC) and frozen. Cryosections of 20μM were cut using Cryostat Cryostar NX70. The slides with the sections were rehydrated in PBS for 15 minutes and surrounded by a hydrophobic barrier using a hydrophobic pap pen. Sections were then blocked for 1 hour in blocking solution (5% Normal Goat Serum, 0.05% BSA, 0.2% Triton X-100 in PBS), and incubated with primary antibodies diluted in blocking buffer: anti-O4 (1:200); GFAP (1/500); MAP2 (1:1000), Tyrosine-Hydroxylase (TH) (1/500), Neurofilament (NF) (1/500), GIRK2 (1/250), FOXA2 (1/500), AQP4 (1/500), S100b (1/500) at RT for 1h. Fluorescent-labeled secondary antibodies (Invitrogen) were added at a dilution of 1:500 and incubated for 1 hour. Nuclei were identified with Hoechst 33258 (1:5000) diluted in PBS and incubated with the cryosections for 10 minutes at RT. Cover slides were mounted using Aqua-Poly/Mount mounting medium and imaged using confocal microscopy (Leica TCS SP8 confocal).

#### Tissue dissociation and processing preparation for FC

Alll hMOs were dissociated with a combination of enzymatic digestion and mechanical dissociation. For the main 9 hMO samples the hMOs three individual hMOs from each of the data set of nine samples were removed from shaking cultures and combined into one 15mL tube. Pooled hMOs were washed three times with Dulbecco’s PBS (D-PBS) to completely remove remaining culture media. Then, after completely removing D-PBS, 2mL of TrypLE express without phenol red was added to each sample. The hMOs were incubated at 37°C for ten minutes then removed to be subjected to mechanical dissociation by pipette trituration (slowly pipetting up and down ten times). The incubation and the pipette trituration are repeated twice more. Afterwards, 8mL of D-PBS was added to the samples to stop the enzymatic reaction. The samples were filtered through a 30μm filter (Miltenyi Biotec) to remove any clumps remaining after digestion and dissociation. Samples were washed twice more with D-PBS.

For the AIW002 hMOs used for sorting and in the time course experiments 7-20 individual hMOs were removed from a bioreactor and combined into one 50mL tube. Pooled hMOs were washed three times with Dulbecco’s PBS (D-PBS) (Wisent) to completely remove remaining culture media. Pooled hMOs were transferred to a gentleMACS M-Tube. Then, after completely removing D-PBS, 2mL of TrypLE express without phenol red was added to each sample. The hMOs inside the M-Tube were next placed on an automated the GentleMACS Octo Heated dissociation device. The settings for the dissociation were as follows: 37°C is ON. Spin -20rpm for 24 minutes. Spin 197rpm for 1 minute. After GentleMACS dissociation, 8mL D-PBS was added to the samples to stop the enzymatic reaction. The samples were filtered through a 30μm filter (Miltenyi Biotec) to remove any clumps remaining after digestion and dissociation. The samples were then washed twice more with D-PBS.

For 2D cell cultures T-flasks containing cells were washed in PBS then incubated at 37°C in 2mL of TrypLE express without phenol red for 5-20 minutes depending on cell type. Cells were washed off the growth surface with a pipette, then manual dissociated by trituration until no clumps were seen and transferred to a 15ml tube. Cells were washed twice in D-PBS.

#### Cell surface antibody staining for FC

After counting and isolating one million cells, single cell suspensions were incubated for 30 minutes at room temperature in the dark with Live/Dead Fixable dye to assess viability. Single cell suspensions were washed twice with D-PBS to remove any excess dye. After, single cell suspensions were incubated for 15 minutes at room temperature in the dark with Human TruStain FcX at a concentration of 5μL per million cells to block unspecific Fc Receptor binding. Single cell suspensions were washed once with FACS buffer (5% FBS, 0.1% NaN_3_ in D-PBS) and then incubated for 30 minutes at room temperature in the dark with a fluorescence-conjugated antibody cocktail in 500 μL of FACS buffer. The following dilutions for the antibody panel were used: CD44 (1/192), AQP4 (1/28), GLAST (1/20), HepaCAM (1/333), CD71 (1/333), CD184 (1/48), CD133 (1/333), CD15 (1/48), CD29 (1/48), CD56 (1/96), CD24 (1/192), O4 (1/31), and CD140a (1/40). The same dilutions were used for the time course assays and the additional cell surface antibody concentrations were used: SSEA-4 (1/80) and CD49f (1/1280).The optimal working dilutions were determined by titrations with similar hMOs and experimental conditions. After incubation, single cell suspensions were washed twice with FACS Buffer and resuspended in FACS Buffer. Samples were placed at 4°C until ready to be analyzed by FC.

In parallel, compensation control staining was performed with the same conditions as the single cell suspensions. The compensation controls used are UltraComp eBeads™ Plus Compensation Beads and ArC™ Amine Reactive Compensation Bead Kit Samples were placed at 4°C until ready to be acquired by FC.

#### Fixation and permeabilization for TH internal antibody labelling for FC

Following cell surface antibody staining of the single cell suspension, all cells were washed twice and incubated with 2% PFA diluted in PBS for 15 minutes at room temperature in the dark. After fixation the cells were washed 3 times with FACS buffer and centrifuged at 350g for 5 minutes. Following washes, the cells were permeabilized (0.7% Tween-20 in FACS buffer) for 15 minutes at room temperature in the dark. Cells were washed once (centrifuge 350g 5 minutes) and incubated in the dark for 30 minutes with the fluorescent-conjugated TH antibody (1/1700). Untagged TH antibody was coupled to the PE flurochrome by the manufacturer’s protocol using Lighting-Link PE Cells were washed twice in FACS buffer (centrifuged 350g for 5 minutes) and resuspended in FACS buffer for further analysis by FC.

#### FC acquisition and cell sorting

Separate devices were used for acquisition only or sorting. For only acquisition the single cell suspensions were acquired on an Attune NxT (ThermoFisher). The information for the configuration of this Flow Cytometer is in Table S12. Daily CS&T performance tracking was done prior to cell acquisition by recommendation of manufacturer. PMT voltages were determined by Daily CS&T performance tracking. Compensation controls were also acquired, creating an acquired compensation matrix. Between 48 000 to 338 000 cells were acquired per sample.

For sorting experiments single cell suspensions were sorted on a FACSAria Fusion (Becton-Dickinson Biosciences). The information for the configuration of this Flow Cytometer is in Table S13. The gating criteria defined by CelltypeR using *hyergate* was input into the Becton-Dickinson Bioscience FACSAria built in software as shown in Figure 5A. Daily CS&T performance tracking was done prior to cell acquisition by recommendation of manufacturer. PMT voltages were determined by Daily CS&T performance tracking. Compensation controls were also acquired, creating an acquired compensation matrix. Cells were sorted using the largest nozzle size (100 μm) and the lowest pressure (20 psi) possible. Three separate tubes of AIW002-02 hMOs were sorted into FACS buffer and combined into one sample for each population. These four populations were sorted into four gates and were sorted until the sample with fewest cells (Neurons1) contained 100,000 events.

#### Single cell sequencing of FC sorted populations

The sorted samples were centrifuged for 5 minutes at 400g and resuspended in 250 ml of D-PBS + 0.1% BSA. The cell concentrations were calculated with FACSAria Fusion. The single cell suspensions were diluted to 1000 cells/ml targeting ∼15,000 cells captured for sequencing. For each sample cells were added to the reaction mix in the Chromium Next GEM Single Cell 3’ reagent kit v3.1 into the Chromium NextGEM Chip G as per manufacturer protocol and run on the 10X Chromium Controller for GEM creation. All proceeding thermocycler steps were carried out on a Bio-Rad C1000 Touch thermal cycler. Following GEM-RT incubations, samples were stored at 4°C overnight. Post GEM-RT cleanup and cDNA amplification were carried out per manufacturer protocol. Samples were stored at -20°C until they were processed for library generation. 3’ gene expression and cell surface protein libraries were constructed per manufacturer protocol and stored at -20°C until sequencing submission. 25 μL of each sample library were sent for next generation sequencing at the McGill Genome Centre and using the NovaSeq6000 sequencer.

### Method details

#### Analysis overview

For each experiment the cells are live gated in FlowJo (see Figure S2) then exported as fsc files to be analyzed in R the CelltypeR library. Detailed steps of the CelltypeR workflow with all code can be found in the R notebook “CelltypeRWorkFlow.Rmd on the github repository: https://github.com/RhalenaThomas/CelltypeR. All live cell samples to analzye are placed in one folder and read into R. A data frame with intensity measurements for each marker for all samples within the experiment to be analyzed is created. The expression data can then be transformed and aligned to remove batch effects. A Seurat single cell object is then created for further analysis. A function for clustering optimization to compare clustering methods and parameters and visualize results is run generating summarize statistics to compare clustering methods and parameters. The optimal method is to generate clusters to annotate. If the dataset is the first FC dataset with the marker panel, then CelltypeR CAM function and marker visualizations are used to assign cell types to clusters. Using the CelltypeR RFM functions a predictive classifier is made for subsequent dataset. For the RFM and Seurat label transfers are used in addition to CAM and marker visualization for repeat experiments. After cell types are annotated the number of cells per sample and expression levels of markers within cell types are measured. The data can then be used for statistical analysis between different groups of interest using CelltypeR functions. Marker threshold for gating to isolate cells can be defined using *Hypergate*.

#### FC data cleanup for analysis

The data generated was cleaned up using FlowJo (version 10.6) (Becton-Dickinson Biosciences). Briefly, a starting gate was used to select appropriate cell size (X: FSC-A, Y: SSC-A). A second gate was used to discriminate doublets from the analysis (X: FSC-W, Y: FSC-H). Finally, the last gate was used to remove dead cells from the analysis (X: LiveDead Fixable Aqua, Y: FCS-A) (Figure S2). After data cleanup, a new .fcs file was generated with FlowJo and exported.

#### Sample processing, batch correction and creation of a seurat object

The .fcs files without dead cells, debris, and doublets created in FlowJo are read into R and processed. The .fsc files contain area, width, and height of the fluorescence signal for each marker as well as the forward and side scatter of the light. The CelltypeR function *fsc_to_fs* selects data from the fsc files and creates a flowset^21^ object. The area values for each channel to represent the expression intensity for each antibody are selected. Each file is treated as a sample and all files within a folder are merged into one object with the sample identities maintained. The function *harmonize* is issued to biexponentially transform, align peaks and reverse transforms the data, all processes are performed by default, but the processing level can be selected. Arguments for which channels have one or two peaks must be entered. The distinction between positive and negative antibody staining is enhanced using raw data is the biexponential transform with default parameters (*a=0.5, b=1, c=0.5, d=1, f=0, w=0)*.^21^ The transformed data is aligned using gaussian normalization in which local maxima are detected above the bandwidth we set to be above 0.05, to avoid picking up noise, each peak is given a confidence score reflecting the height and sharpness of the peak, the threshold for two peaks to be considered too close together was set too 0.05.^20^ Landmarks are then detected and aligned, such that each landmark is shifted to a benchmark, which corresponds to the position of the closest peaks across all samples. After alignment, the data is reverse transformed to improve visualization by UMAP in downstream analysis.

For the hMO samples, the data were aligned to remove batch effects and technical variability. The 2D cultures where not aligned. Using the function *flowset_to_csv* the flowset objects are then converted into a data frame and used as the input to create a Seurat object. The raw expression data and the sample identities are used to create a Seurat^41^ object with the function *make_seu*.

#### Clusters optimization and generation

The function *explore_param* is used to determine the best clustering conditions. The function enables clustering using FlowSom,^22^ Seurat^41^ Louvain network detection and Phenograph,^62^ which also uses Louvain network detection with the addition of an internal Jaccard index. For each method the parameter space can be explored: k neighbours (the number of cells to be considered in generating a nearest neighbour network graph), resolution (a value from 0 to 2 that adjust the minimal distance in similarity between network nodes in the Seurat method, the default is 0.8), k clusters (used by FlowSom to define the max number of clusters). For the 9 hMOs, 9000 cells were randomly selected, and one sample, all the cells (1578) cells were selected before transformation and alignment. Then we compared all cluster methods and explored the parameter space. The *explore_param* function outputs intrinsic statistics and produced UMAPs and heatmaps for visualization. The intrinsic statistics calculated are the Silhouette score (values of -1 to 1, where higher values indicated better quality clusters),^63^ the Calinski-Harabasz index (where higher values indicated better quality clusters)^64^ and the Davies-Bouldin index (where lower values indicate better quality clusters, and the minimum value is 0).^65^ We selected the Seurat Louvain network detection and used the function *clust_stability* to calculate the RAND Index and standard deviation of the number of clusters across 100 iterations of clustering with different random start points for a range of cluster resolutions (0.25, 0.5, 0.8, 1.2). A higher RAND index and lower standard deviation in repeated clustering of the same data indicates a higher stability of clustering. After the desired clustering conditions are selected the function *get_clusters* was used to perform the clustering and add the cluster indexes into the Seurat object. The process is different depending on the clustering methods. For the Seurat method *get_clusters,* scales the data setting the mean expression value to 0 and the standard deviation to 1 for each marker, performs a principal component analysis (PCA) which is the input to generate the shared nearest neighbor network (SNN) which is input into the Louvain network detection algorithm that optimizes modularity. For the Seurat clustering method, the number of neighbours for the SNN (k), the number of components from the PCA (pcdims) and the resolution need to be entered as arguments. Seurat clustering was used in all the analysis with the following parameter settings: For the 9 hMO samples where 9000 cells were selected: k = 60, pcdim = 1:12, resolutions = 0.8. For the 9 hMO samples using all cells: k = 60, pcdim = 1:12, resolutions = 1.5. For 2D cultures: k = 25, pcdim = 1:12, resolutions = 0.25. For the AIW002-02 organoids used for sorting: k = 60, pcdim = 1:12, resolutions = 1.2. For the AIW002-02 time course data: k = 80, pcdim = 1:12, resolutions = 1.0.

#### Cluster annotation

For the 2D cultures cell types were assigned by the visualization of expression values, the known original cell type, and the overlap in space on the UMAP. Heatmaps grouped by cluster numbers utilizing the function *plotmean* were produced to visualize the expression per cluster. Cell type annotation was performed on the subset of 9000 cells using visualization and the CAM method we created. To predict cell types from a reference matrix using the CAM method the function *find_correlation* was utilized and run separately three times with different threshold for the minimal Pearsons correlation coefficient, R value (min_corr = 0.1, 0.3, 0.553). The threshold for cell type predictions names being with a merged names was kept at the default setting of 0.05). The CAM prediction results were visualized with the function *plot_corr.* The CAM function calculates the correlation between each cell in the FC data with the cell types defined in the reference matrix using a data frame for both the reference and test data. The CAM cell type predictions are then added into the Seurat object. The functions *plot_lab_clust* to visualize the frequency of CAM prediction per cell type and the *get_annotate* is used to get the top cell type prediction for each cluster. UMAPs with the expression levels of each marker targeted in the antibody panel as well.

The annotated subset cells from the 9 hMO was used to train a Random Forest Classifier (RFM) that utilizes *randomForest*^50^ package and the *caret* package to explore parameter space. The function *RFM_train* runs a user defined set of number of starting features (markers) to include (mytry), the number of decision trees, and the number of the maximum nodes possible (max nodes). To avoid overfitting, cross validation is used with parameter space exploration, and parallel processing is possible to decrease computation time. However, the function can run in series needed. The features (markers) to be included in training the model must be defined. The data is split 80/20 training/test by default. The cross-validation function creates training/validation sets within the function. The function requires a labelled Seurat object as the input. For the RFM trained on the subset data to annotate the full 9 hMO dataset all markers in the antibody panel were used with a 3-fold cross validation. The best conditions from the explored parameter space mytry (5-7), max nodes (15-18) and number of trees (1500, 1800, 2000) were mytry = 6, max nodes = 15 and number of trees = 1500. A separate RFM was trained to annotate the time course data including only the overlapping markers and using the full 9 hMO annotated data object, the best conditions were mytry = 5, max nodes = 17 and number of trees = 1800.

To annotate all cells in the 9 hMO dataset and the time course data a combination of CAM prediction, RFM predictions, Seurat label transfer and marker visualization were utilized. The cell types were predicted using the trained RFM with the function *rfm_predict.* For the Seurat label transfer we made the function *seurat_predict* that follows the Seurat workflow for label transfer combined into one function.^41^ The function requires an annotated Seurat object and the test Seurat object. Both objects must have a PCA, which is used to find anchor points between the annotated clusters and the cells to be predicted. For each prediction method the most frequently predicted cell types within each cluster are calculated with the function *get_annotation* and summarized into one data frame using the function *annotate_df*. For all dataset the function *annotate* was used to add the final annotations defined from the combination of the input.

#### Creation of the predicted expression matrix for CAM algorithm

Expression values for expected cell type were combined from several sources shown in Table S1 and key resources table. For each transcriptional dataset the gene transcripts corresponding to the marker proteins were selected for each brain cell type of interest. The expression values for total RNA from sorted cells were taken from the extended data from Zhang et al. 2016.^43^ For the scRNAseq data from human adult brain,^48^ developing brain,^44^^,^^45^^,^^47^ and organoids,^1^^,^^49^ read count matrixes and metadata were collected from UCSC Single Cell Browser,^60^ The Broad Institute or GEO and used to generate Seurat objects. The authors cell type annotations were used and the mean transcriptional values for each marker and cell type were selected. Microglia are found in brain tissue but are not expected to be present in hMOs due to their mesodermal lineage and thus were not included in the reference matrix. In the first (13 antibody panel) there is not a specific DA marker and so we only define NPCs and neurons. The DA subtype is specified in the second (time course) panel reference matrix. For the antibody O4, the epitope is a glycoprotein, and the specific corresponding gene is unknown, however the gene NKX6.2 is a marker of mature oligodendrocytes, with expression highly correlated to O4 protein detection.^66^ For SSEA-4, another glycoprotein epitope we used gene encoding the SSEA4 synthase enzyme.

After cell type by marker expression matrixes were generated from each reference dataset, a z-score normalization transforming expression values to between 0 and 1 was applied to each dataset. The mean expression values from all brain samples and all organoids were calculated, then the mean expression between these two matrixes was calculated and z-scored. The overall mean RNA expression values were combined with the z-score normalized FC values from the 2D cell cultures for the 13-antibody panel. For the TH time course panel, we could only use the overlapping markers for the FC expression. For RNA seq data we used the gene expression equivalents to each marker in the two panels. Not all gene equivalents for the protein markers were available from all cell types or databases. We only acquired FC expression values from iPSCs, NPCs, neurons, astrocytes, OPCs and oligodendrocytes, these cell types have 1:1 weighting between protein and RNA expression levels. The endothelia, epithelia and radial glia value are from RNA expression only.

#### Reverse engineered using *hypergate* and gate application in FlowJo

Cell types were selected in the full annotated hMO dataset and input into the *hypergate* function.^54^ A table of predictions was output. For each cell type the threshold levels for each antibody required to define the cell type were output. These thresholds are ordered from most to least important. For testing the gates, manual gating was applied in FlowJo with the top gate for each cell type in each sample being set as live single cells. The gates were applied to one AIW002-02 sample and then applied across the other samples. For gating, the two antibodies were visualized by scatter plot and a box was drawn selecting the thresholded cells from the antibody pair. The gated cells were then selected and gated with the next pair of antibodies until all thresholds were applied. The final gated cell types from all samples were exported as fsc files and read into R following the CelltypeR workflow. To apply gates to FACS four selected samples examined each cell type gate and selected gates which mostly exclusive for different cell types. The neurons can be separated from glia and then split into two populations and the glia can be split into two populations.

#### Single cell sequencing analysis

The FASTQ files processed using 10X CellRanger 5.0.1 software are installed on the Digital Research Alliance of Canada: Beluga computing cluster. For each of the four sorted populations, the CellRanger output files raw expression matrix, barcode, and feature files were used to create a Seurat data object with minimum filtering of RNA features > 100. After this point data was run locally and all details can be found in the R notebook, ‘scRNAseq_processing’. RNA features, RNA counts, and percent mitochondria were checked for quality control for each sample: Neurons1, Neurons2, Glia1(astrocytes), and Glia2 (radial glia). Further filters were applied.

For the glia samples, there were a large number of cells after filtering. The Seurat function *HTODemux* was used to assign Hashtag (replicate labels). For neuron samples and radial glia all cells were selected, for glia1/astrocyte sample the original count was very high. To increase selection of true cells, cells with assigned hashtags were used for further processing. For all samples, doublets were removed using Doublet Finder.^67^ The expected percent of doublets estimation was based on the number of cells present after filtering and the 10X version 3 user guide. For each sample data was normalized, variable features selected, PCA and UMAP dimensional reductions were performed, and clusters detected with Louvain network detection (25 dimensions and 43 neighbours selected, and a range of resolutions was run).

Clusters were annotated using a consensus between expression of known cell type markers from gene lists, analysis of cluster markers, and cell type predictions of reference using Seurat find anchors and label transfer. Subtypes of major cell type groups were observed and at this point these clusters were all merged into major cell types. The individually processed samples were then merged, samples were down sampled to balance the data and decrease processing time.

After the four samples were merged the standard processing and clustering was run again using the same settings. Clusters were annotated again, retaining subtypes of each cell type, and identifying the DA neurons. Each subtype was analyzed to find subtype markers and analyze using GO biological processes. Reference datasets using Seurat anchors and label transfer predictions were used to define subtypes of cells. All the scRNAseq data sets in Table S1 were used. A threshold for cell type assignment was set to 0.5 for brain reference data and 0.8 for hMO scRNAseq data. Developing cortex, forebrain and whole brain datasets were all reconstructed into Seurat objects from the UCSC cell browser following the website instructions.^60^ Each reference was down sampled in Seurat to reduce the total cell number to less than 50000. For single nuclear RNAseq data from human adult post-mortem brains three separate reference sets were created. The expression matrix, barcodes, and feature files were used to create a Seurat object. The metadata for cell type and cell subtype annotations data was added from the UMAP_tsv files provided by Kamath et al.^48^ The brain region data was added from the provided meta data file. The adult midbrain was subset by brain region selecting only the midbrain cells. The DA subtypes and astrocyte subtypes were separately subset by using the main cell type annotation.

1. All cell types (astrocytes, oligodendrocytes, microglia, endothelial cells, DA neurons and other neurons). This was used in the initial cell type annotations.
2. DA neuron subtypes, used to try to identify DA subtypes. All the hMO subtypes matched only one subtype from adult brain.
3. Astrocyte subtypes, used to identify astrocyte subtypes. All astrocyte subtypes in hMO matched one subtype.

After annotating the main groups of cell types (DA neurons, neurons, astrocytes, radial glia, NPCs, mixed) subtype annotations were applied. To annotated subtypes, the main cell type was subset. The *Seurat* find all markers function was used allowing both up and down regulated gene markers of the clusters within each main cell type. The top 5-10 marker genes sorted by highest Log2 Fold change with significant adjusted p-values were further investigated by literature search to determine the cell subtypes.

### Quantification and statistical analysis

#### Cell type proportion tests

Permutations tests were selected over the commonly used Chi square or Fisher’s exact test because the later tests assume the observation are independent and normally distributed. However, the proportion of the cell types within a sample is directly dependent on the other cell types confounding these tests. In permutation tests sample labels are iteratively scrambled and used as the input into a statistical test generating a null distribution.^51^ The correct contrast distribution(s) are then compared to the null distribution. For comparisons between two groups (two iPSC lines or time points), the function *permutation_test* from the R library scProportionTest was used to calculate log2-fold changes in fractions between cell types. Next, p-values are calculated for each cluster (cell type) by comparing how many times the permuted log2-fold changes are as extreme as the observed log2-fold change. These p-values are adjusted for multiple comparisons using the false discovery rate (FDR) method, providing a measure of the statistical significance of the observed differences in proportions. For a comparison across samples, we created a custom function that runs permutations in a one-way ANOVA and calculates p-values comparing the distributions between samples to the null distributions. The CelltypeR function *permutation_test_multi* utilizes the ANOVA permutation test from the *Permuco* R library^52^ and applies the test to the single cell FC data in a Seurat object.

#### Differential expression tests

Statistical analysis of marker expression between iPSC lines for each cell type were performed using the CelltypeR *Prep_for_stats* and *run_stats*. The *Prep_for_stats* function takes in a Seurat object with metadata containing the cell type annotation and variable that are to be compared and creates a data frame with the expression values selecting the user define markers, cell type annotations and variables to compare. The data frame is the input for *run_stats*, which runs either a one-way or two-way ANOVA with Tukey’s post hoc tests for main effects and interactions. The statistic function uses the base R functions *aov* and *TukeyHSD*. Users can select to use samples or cells as replicates, however using cells as replicates will not be revealing in most cases as there will be so much power that all comparisons will be significant. The function permits a second or third variable by running a loop to analyze each level of this variable separately. To compare marker expression across the three healthy iPSC lines for each cell type a two-way ANOVA with n=3 samples for each iPSC line was run with iPSC line and marker as the dependent variables running a loop over cell types. Main effects, interaction effects and Tukey’s post-hoc test with correct p-values were all output.

## Supplemental information

Document S1. Figures S1–S23 and Tables S1–S13

Table S14. Quantification of live cell counts out of total events, related to Figures 1, 2, 3, 4, 5, 6, and 7 Flow Jo gating of FC events analyzed to generate cell counts for all samples included in the manuscript. Cell counts after dissociation and prior to antibody labeling as well as after antibody labeling and gating for analysis are provided.

Table S15. Cell counts for FACS population used for scRNA-seq analysis, related to Figure 6 Total event counts passing through each gate for the neurons1, neurons2, astrocytes and radial glia populations sorted from midbrain organoids. Replicates were then pooled, and the percent live cells were calculated.
