# Supplementary material for: CelltypeR: A flow cytometry pipeline to characterize single cells from brain organoids
Source: iScience. 2024 Jul 30;27(9):110613. doi: 10.1016/j.isci.2024.110613 (PMC11367488; doi:10.1016/j.isci.2024.110613)
Supplement: Document S1. Figures S1–S23 and Tables S1–S13 [file mmc1.pdf]

iScience, Volume *27*

Supplemental information

CelltypeR: A flow cytometry pipeline to characterize single cells from brain organoids

Rhalena A. Thomas, Julien Sirois, Shuming Li, Alexandre Gestin, Ghislaine Deyab, Valerio E.C. Piscopo, Paula Lépine, Meghna Mathur, Carol X.-Q. Chen, Vincent Soubannier, Taylor M. Goldsmith, Lama Fawaz, Thomas M. Durcan, and Edward A. Fon

Supplemental Figures

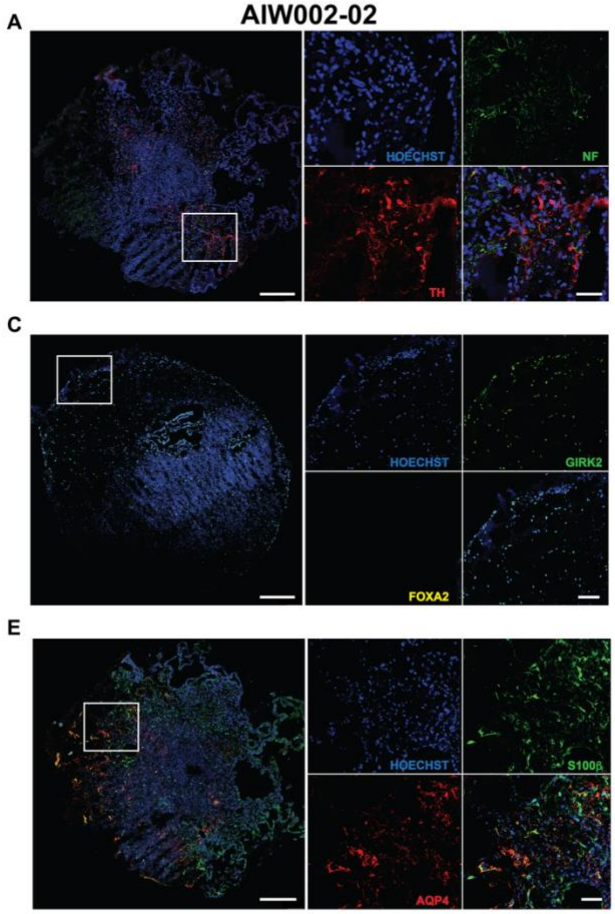

Figure S1: Example images with IF characterization of cell types in hMO 9 months in culture. Related to Figure 1. Cryosection with immunostaining of iPSC lines AIW002-02 (left) and AJG001C (right). Whole hMO sections with merged signals are shown in the left panel, scale bars are all 250 µm. The area in the white rectangles is enlarged on the right with each staining shown separately, the scales bars shown in the merged images are all 50 µm. Nuclei are labelled with Hoechst and shown in blue. **A, B)** Tyrosine hydroxylase (TH) marker of DA neurons in red and neurofilament (NF) a general neuronal marker in green, indicate DA neurons are present in hMOs from both iPSC lines. **C,D)** FOXA2 a neural progenitor marker of DA neuron lineage is absent at this late stage (would be yellow). GIRK2 (green) is expressed in DA neurons of the SN and is present in the hMOs. **E,F)** Astrocyte markers S100b (green) and AQP4 (red) indicate astrocytes are present in the hMOs. Scales bars are 250 µm in the full hMO images and 25 µm in the zoomed images.

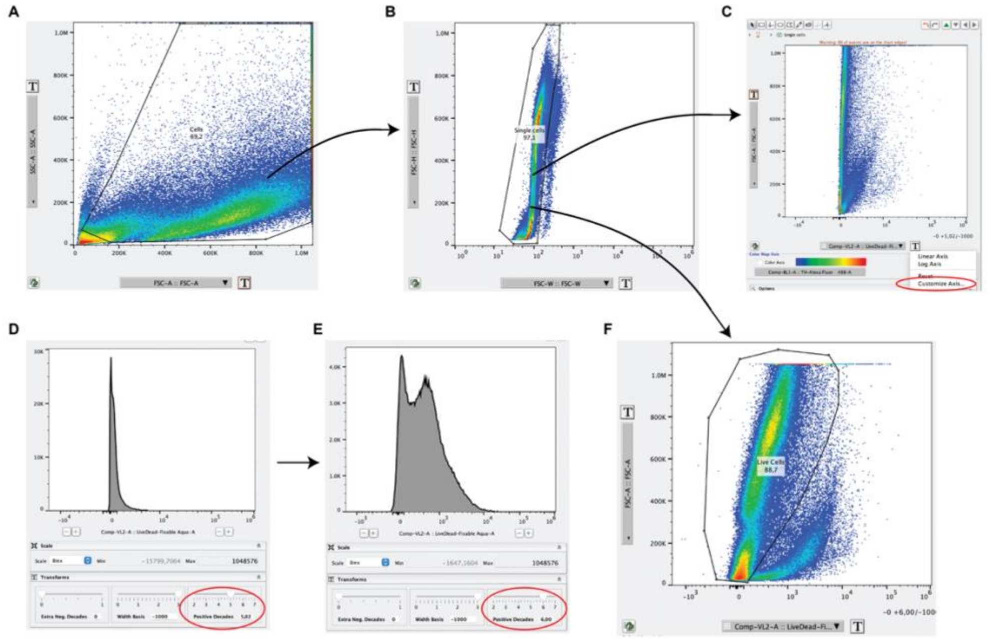

Figure S2: FlowJo FC data cleanup to obtain live single cells for analysis. **Related to Figure 1 and STAR methods** The data generated was cleaned up using FlowJo (version 10.6) (Becton-Dickinson Biosciences). **A)** A starting gate was used to select appropriate cell size (X: FSC-A, Y: SSC-A) to remove debris and select cells. Double clicking the gated cells will give the selected population. **B)** A second gate was used to discriminate doublets from the analysis (X: FSC-W, Y: FSC-H), adjust the x-axis (FSC-W) to log scale. (By clicking the 'T'). Single cells are selected. **C)** Open the selected single cells and change the x axis to the FSC-A-LiveDead Fixable Aqua channel. Click the 'T' and select 'Customize Axis' to adjust the scale. **D)** Custom axis window with the histogram of the LiveDead Fixable Aqua channel. Adjust the "positive decades" (circled in red) until two peaks are seen. **E)** Two peaks are seen on the LiveDead Fixable Aqua histogram. Click apply. **F)** Finally, the last gate was used to remove dead cells from the analysis (X: LiveDead Fixable Aqua, Y: FCS-A) by drawing a gate around the unstained live cell population. After data cleanup, a new .fcs file was generated within FlowJo and exported for analysis for each FC sample.

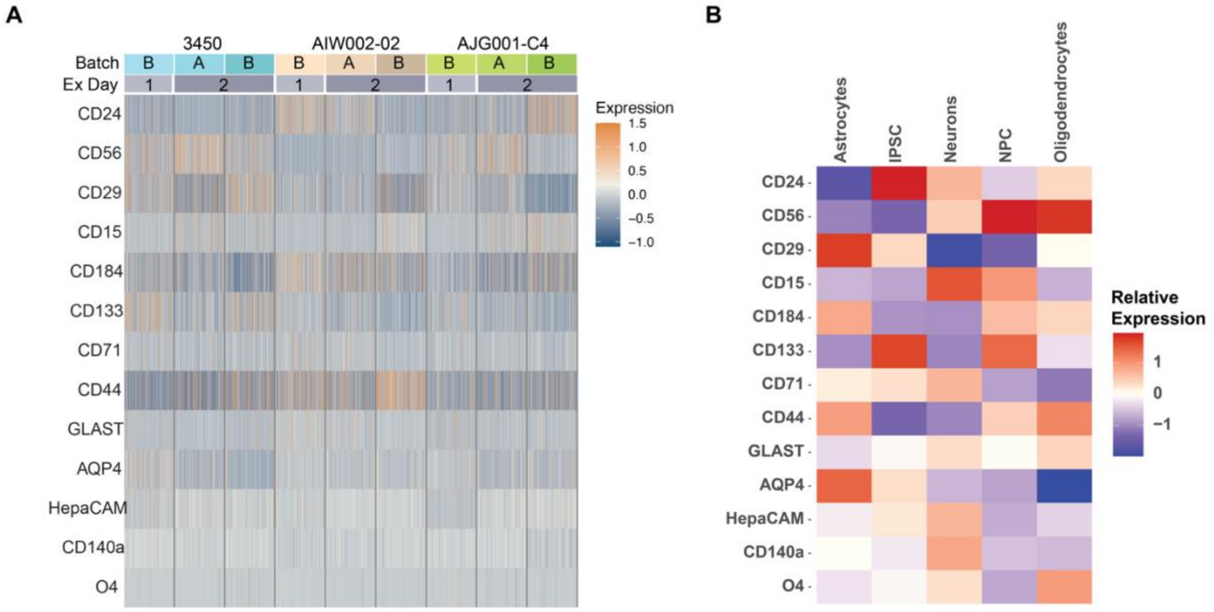

Figure S3: Expression of the 13 protein markers varies across cells in hMOs. Related to Figure 1. Protein expression levels measured by FC in a subset of cells from 9 different hMO samples. The three iPSC lines (3450, AIW002-02-02, AJG001-C4, two batches (A and B) and two different experiment days (1 = 06/03/2020, 2 = 17/03/2020) are indicated at the top. Samples were processed identically on each experiment day and the same tubes and dilutions of antibodies as well as the same Flow cytometer settings were applied, staining and acquisition was performed on two days (1 and 2). Batches A and B each contain the three iPSC lines and were initiated from the same iPSC cultures seeded 3 weeks apart. A random sampling of 200 cells are shown in the heatmap (each bar is a single cell).

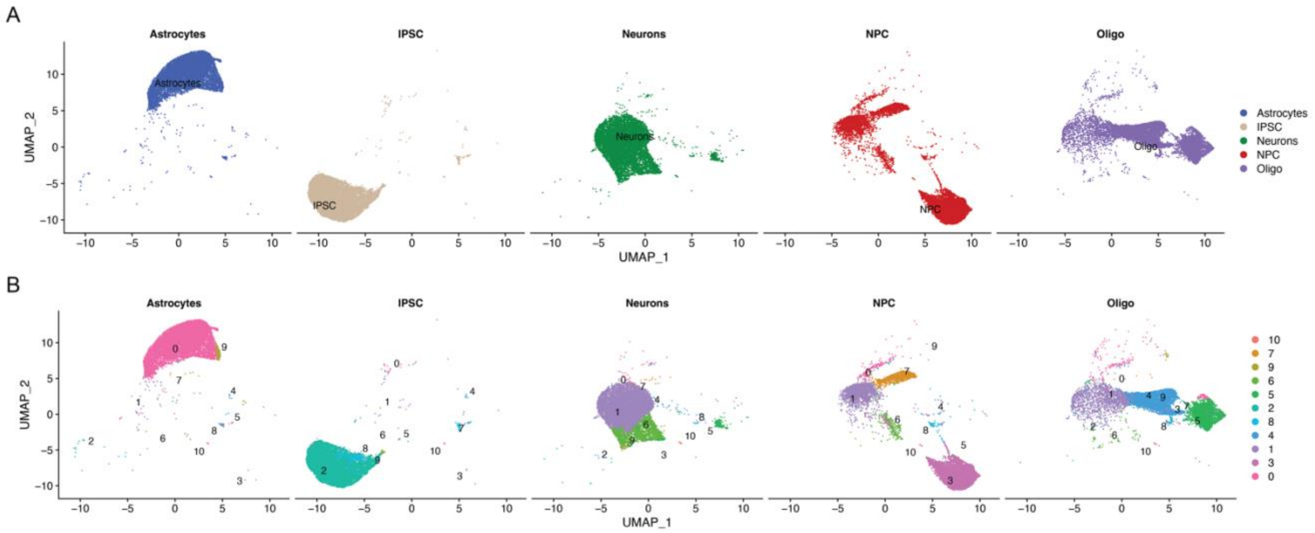

Figure S4: UMAPs of 2D cell cultures and clusters. Related to Figure 2. A) UMAP showing cells split by the original culture type and coloured by the original culture type. B) UMAP of cells split by the original culture type and colour by the clusters identified by unsupervised Louvain network detection. Cells from 2D cultures were harmonized using peak alignment. FC measurements were acquired on two experimental days, astrocytes, DA NPCs and oligodendrocyte cultures used on both experiment days (1 = 06/03/2020, 2 = 17/03/2020). DA neurons, were measured on experiment day 1 and iPSC were measured on day 2. The data from both days were pooled and then cells were randomly down sampled to 10000 cells per culture type, n=50000 cells. All cell cultures are from the AIW002-02 iPSC line.

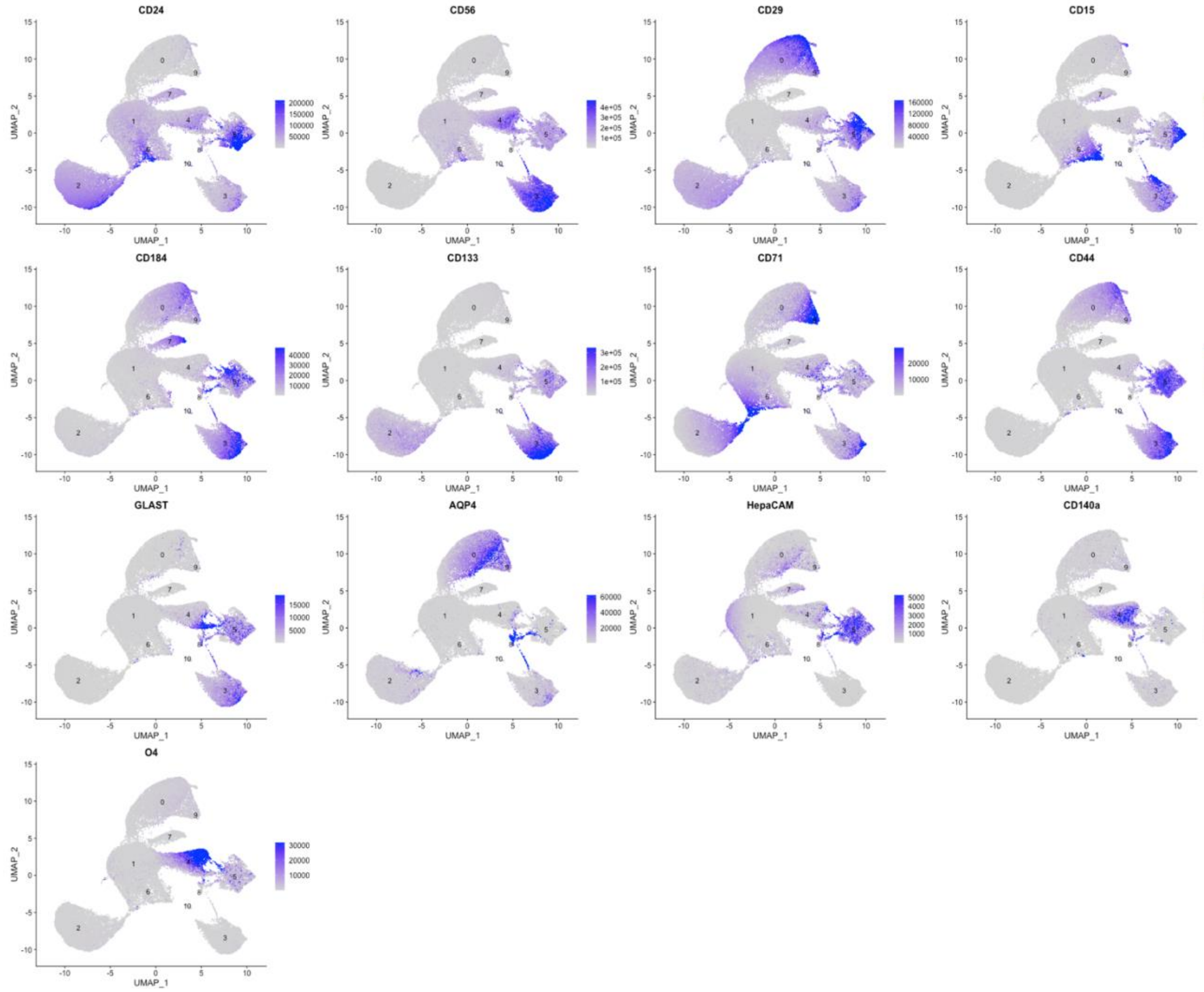

Figure S5: UMAPs of 2D cell cultures with clusters labelled. Related to Figure 2. FC marker expression from the area under the curve of mean intensity per cell followed by normalization is labelled above each plot. The UMAPs are coloured intensity relative to the normalized expression value for each marker, each scale is indicated on the right of the corresponding UMAP. Louvain network detection cluster numbers are indicated on the UMAPs. FC measurements were acquired on two experimental days, astrocytes, DA NPCs and oligodendrocyte cultures used on both experiment days (1 = 06/03/2020, 2 = 17/03/2020). DA neurons, were measured on experiment day 1 and iPSC were measured on day 2. The data from both days were pooled and then cells were randomly down sampled to 10000 cells per culture type, n= 50000 cells.

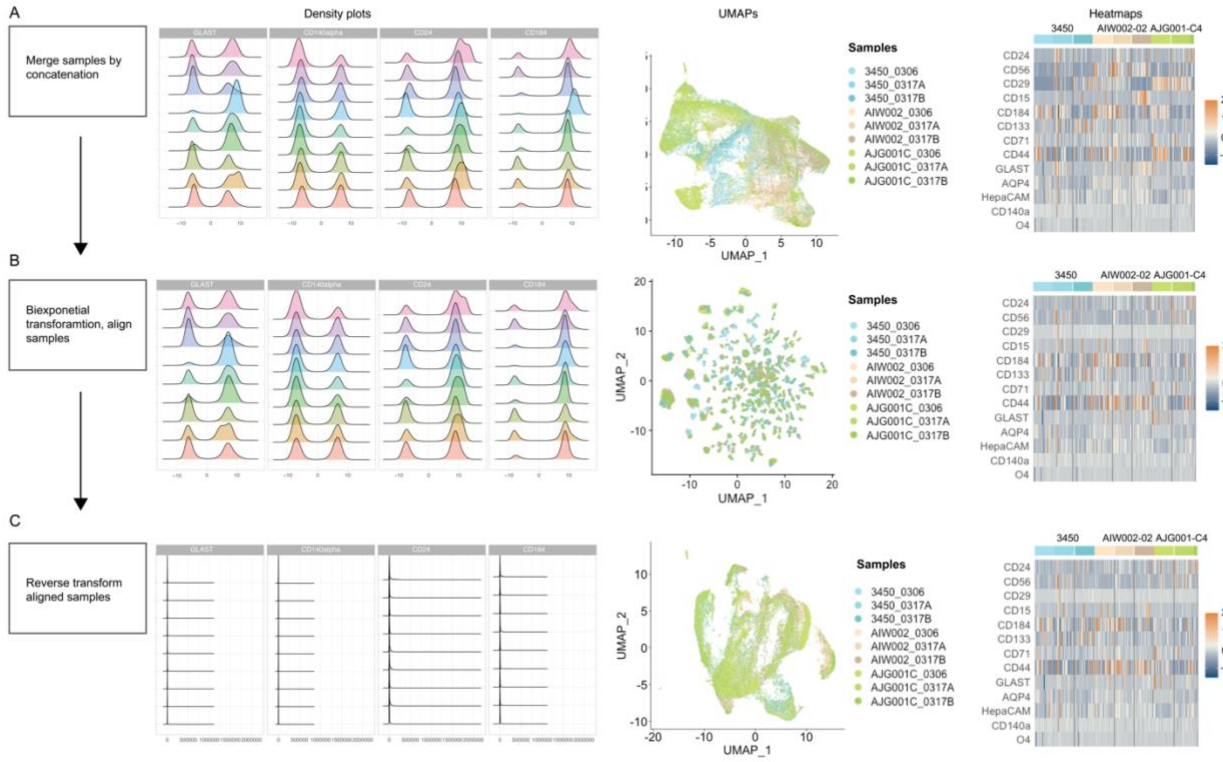

Figure S6: Preprocessing of fsc files exported from FlowJo, sample alignment and reading into R. Related to Figure 3 and STAR methods. A) Samples are merged by concatenation and not transformed or aligned in preprocessing. Files can be saved at this level of processing and one can proceed with the rest of the CelltypeR workflow if desired. For individual 2D iPSC derived cell lines, processing was stopped at this step. **B)** The merged expression data is biexponentially transformed and aligned by shifting means to match peaks between samples. **C)** The merged, biexponentially transformed, and aligned data is reverse transformed removing the biexponential transformation. The full processing was applied for hMO samples to remove experimental variability. Left panel indicates the data processing performed. Density plots visualize the distribution of the FC intensity values. Merged samples and biexponentially transformed samples (A) show that there in the expression distributions between channels. The rows in the plots correspond to the different samples. Four example markers are displayed. The alignment shown in B is the result of shifting the two peaks toward the mean across samples performed by the harmonize function. The density plot in C shows the distribution of expression after alignment and reverse transformation back to a log distribution. The UMAPs visualize cells from the 9 hMO samples indicated by colour in the sample legend. Seurat was used to scale before PCA and UMAP dimensional reductions. **A** shows the merged data before transformation, **B** shows the transformed and aligned data, **C** shows the aligned data, reverse transformed. The UMAP in A compared to C shows the difference between plotting the merged data compared to the aligned data. Right panel indicates heatmap of the marker expression levels across each sample. Scale bars indicate the relative expression and are matched in the heatmap and UMAP plots. Normalized expression is plotted. The data shown is 9000 cells from each of 9 hMOs (accept AJG001C batch A, experiment day 2, n=1578 cells), three iPSC lines (AIW002-02, 3450, and AJG001C), from two batches (A and B), acquired on two experiment days (1 = 06/03/2020, 2 = 17/03/2020).

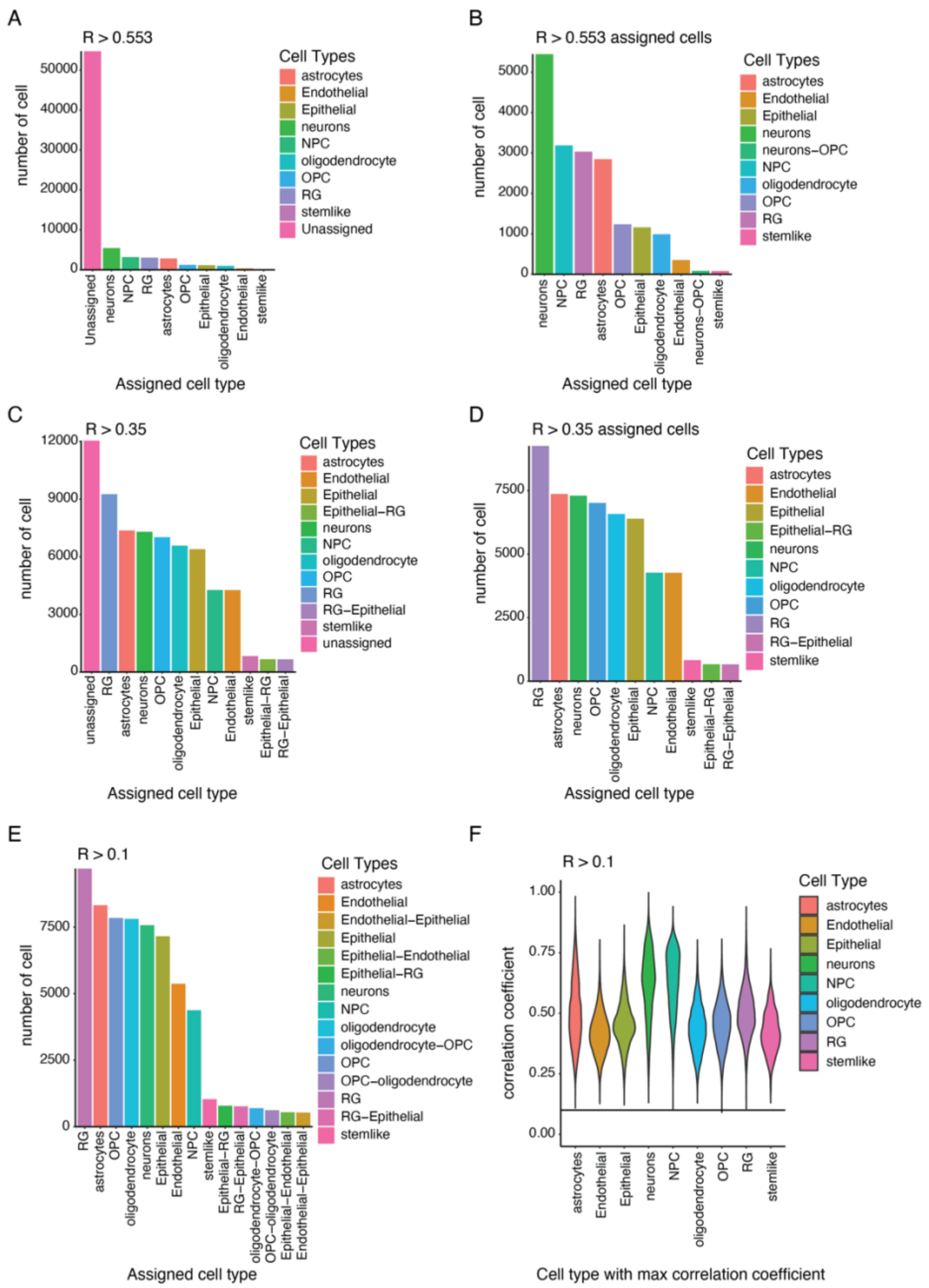

Figure S7: Comparison of different R thresholds for CAM to predict cell types. Related to Figure 3. A) Bar chart showing cell counts for assigned cell types and cells left unassigned with the significant R threshold of 0.553. **B)** Bar chart for cell counts excluding the unassigned cells for the R threshold of 0.553. **C)** Bar chart showing cell counts for assigned cell types and cells left unassigned with R threshold of 0.35. **D)** Bar chart for cell counts excluding the unassigned cells for the R threshold of 0.35. **E)** Bar chart showing cell counts for assigned cell types with R threshold of 0.1. All cells pass the threshold with this correlation threshold. **F)** Violin plot showing the max correlation coefficients grouped by the cell type with the max correlation coefficient. The R threshold of 0.1 is indicated by the horizonal line. The data shown is 9000 cells from each of 9 hMOs (accept AJG001C batch A, experiment day 2, n=1578 cells), three iPSC lines (AIW002-02, 3450, and AJG001C), from two batches (A and B), acquired on two experiment days (1 = 06/03/2020, 2 = 17/03/2020).

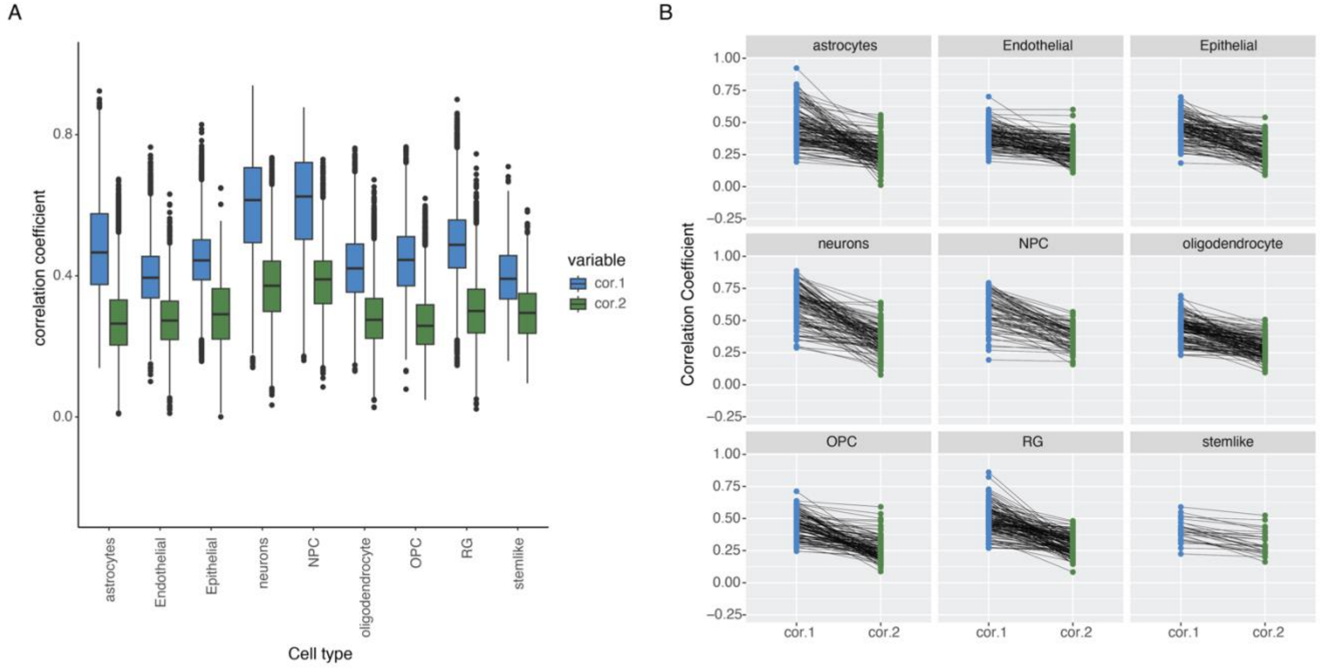

Figure S8: Some cells have high correlation with two cell types in the prediction reference matrix. Related to Figure 3. A) Box plot showing the max correlation coefficient for each cell type (cor1) and the second max correlation coefficient (cor2) for each cell. The cor2 value is not for a specific cell type, it is the second highest correlation value regardless of the cell type. **B)** Connected point plots from a subsampling of cells in each of the predicted cell types with cor1 matched to cor2. The cor1 values are for the indicated cell type and the cor2 values are corresponding to the second max value for each hMO cell. Horizontal lines indicate that the first and second highest R values are close to equivalent. The data shown is 9000 cells from each of 9 hMOs (accept AJG001C batch A, experiment day 2, n=1578 cells), three iPSC lines (AIW002-02, 3450, and AJG001C), from two batches (A and B), acquired on two experiment days (1 = 06/03/2020, 2 = 17/03/2020).

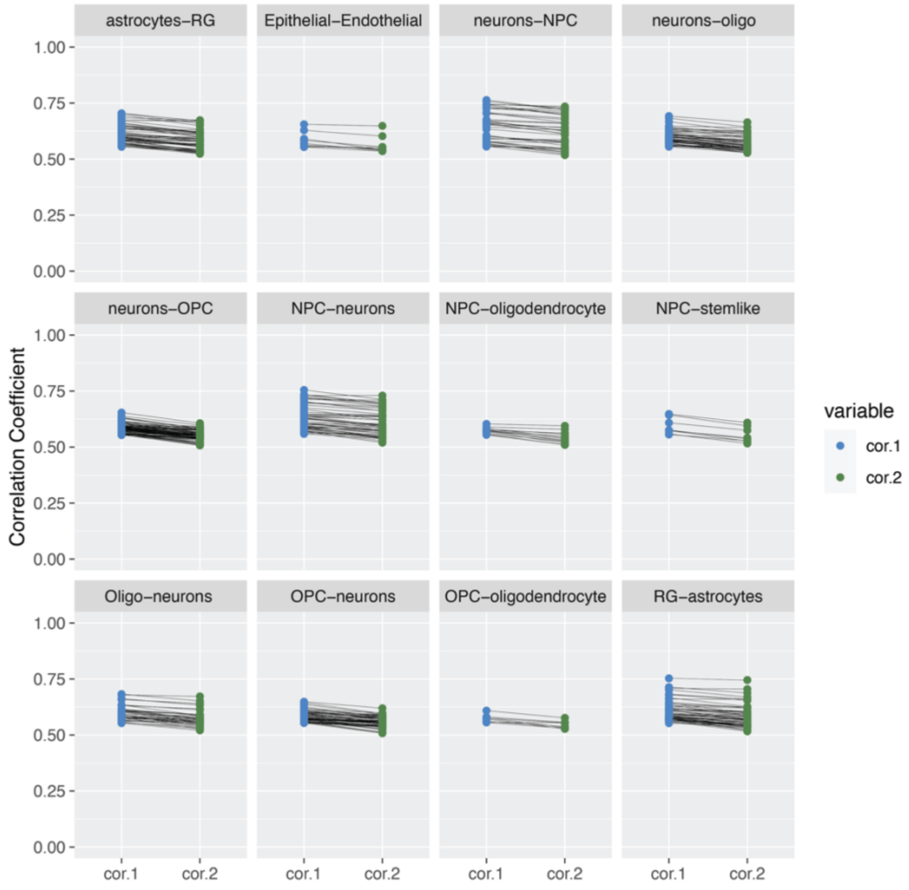

Figure S9: Pairs of cell types with close correlation coefficients to two cell types in the prediction reference matrix could indicate an intermediate cell type. Related to Figure 3. Connected point plots of pairs of predicted cell types with a difference between the first and second highest R values less than 0.05. Only correlation with R value greater than 0.553 were included. Only cell type pairs with more than 8 cells were included. The cell type with the max correlation coefficient is on the left (cor1, blue) and the cell type with the second max correlation coefficient (cor2, green) is on the right. The hMO pairs of correlations coefficients for a given hMO cell are joined by a black line. Cell type names are abbreviated as follows: oligodendrocytes (Oligo), OPC, NPC, RG, neural stem cells (stemlike). Cell types that are a continuum of differentiation, such as neural stem cells and NPCs, or NPCs and neurons have close R values, possibly indicating these cells are starting to express markers of differentiated cell types or retaining some earlier marker expression. The neuron-oligodendrocyte pairs are not a match of a cell type continuum; however, the expression profiles of these cell types overlap. The data shown is 9000 cells from each of 9 hMOs (accept AJG001C batch A, experiment day 2, n=1578 cells), three iPSC lines (AIW002-02, 3450, and AJG001C), from two batches (A and B), acquired on two experiment days (1 = 06/03/2020, 2 = 17/03/2020).

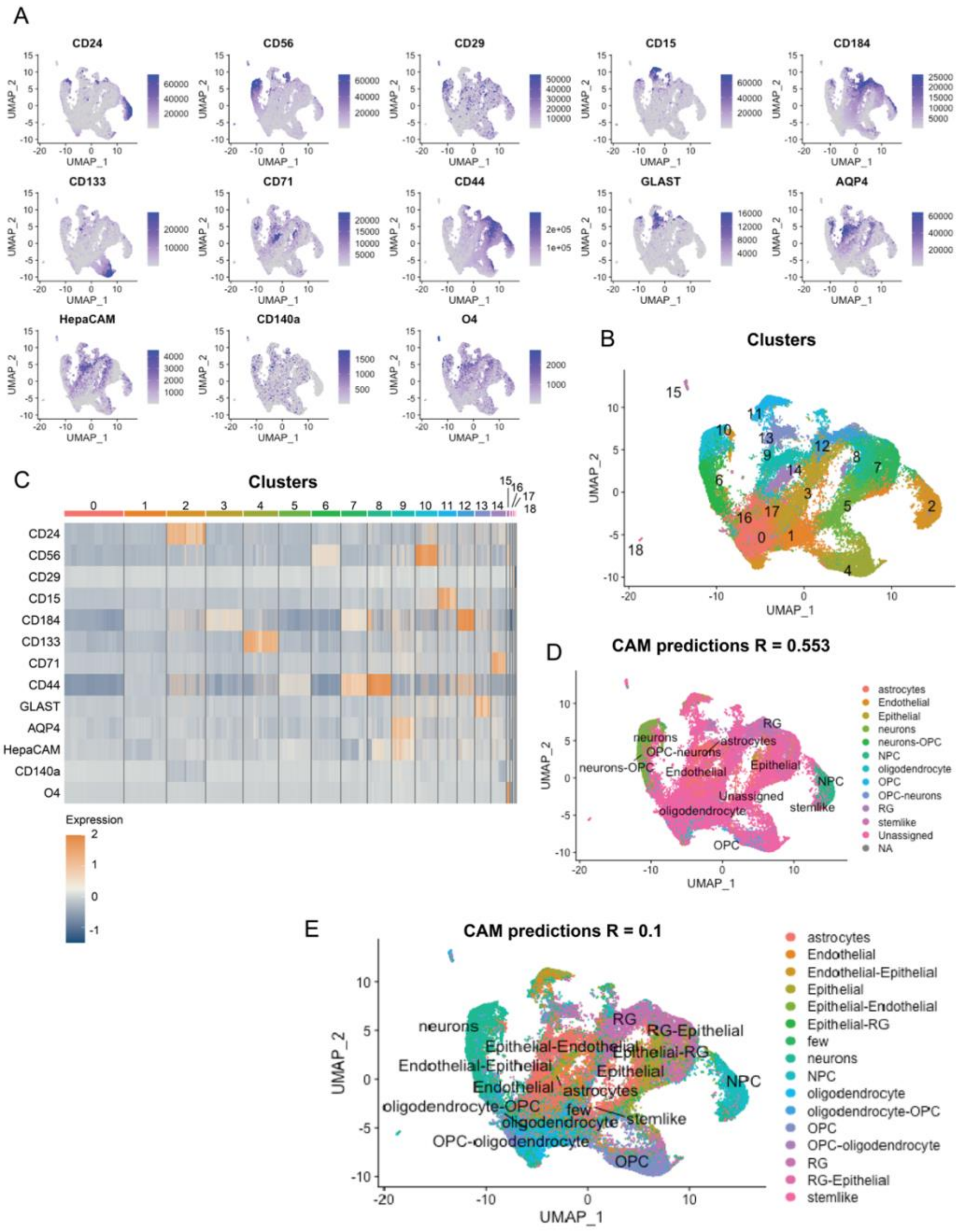

Figure S10: Visualization of antibody expression levels in the subset of hMO cells used for cell type annotation. Related to Figure 3. A) UMAPs of normalized expression for the indicated markers shown as intensity. **B)** UMAP pseudo coloured by cluster, generated by PCA followed by Louvain network detection. Cluster numbers are indicated. **C)** Heatmap of relative expression for each antibody in the panel grouped by cluster. The scale bar is indicated below. **D)** UMAP coloured by CAM predicted cell types with a R threshold of 0.553. Only cell types with > 60 cells are labelled, otherwise these cells are labelled as NA. **E)** UMAP coloured by CAM predicted cell types with a R threshold of 0.1. Only cell types with > 500 cells are labelled, otherwise these cells are labelled as NA. The data shown is 9000 cells from each of 9 hMOs (accept AJG001C batch A, experiment day 2, n=1578 cells), three iPSC lines (AIW002-02, 3450, and AJG001C), from two batches (A and B), acquired on two experiment days (1 = 06/03/2020, 2 = 17/03/2020).

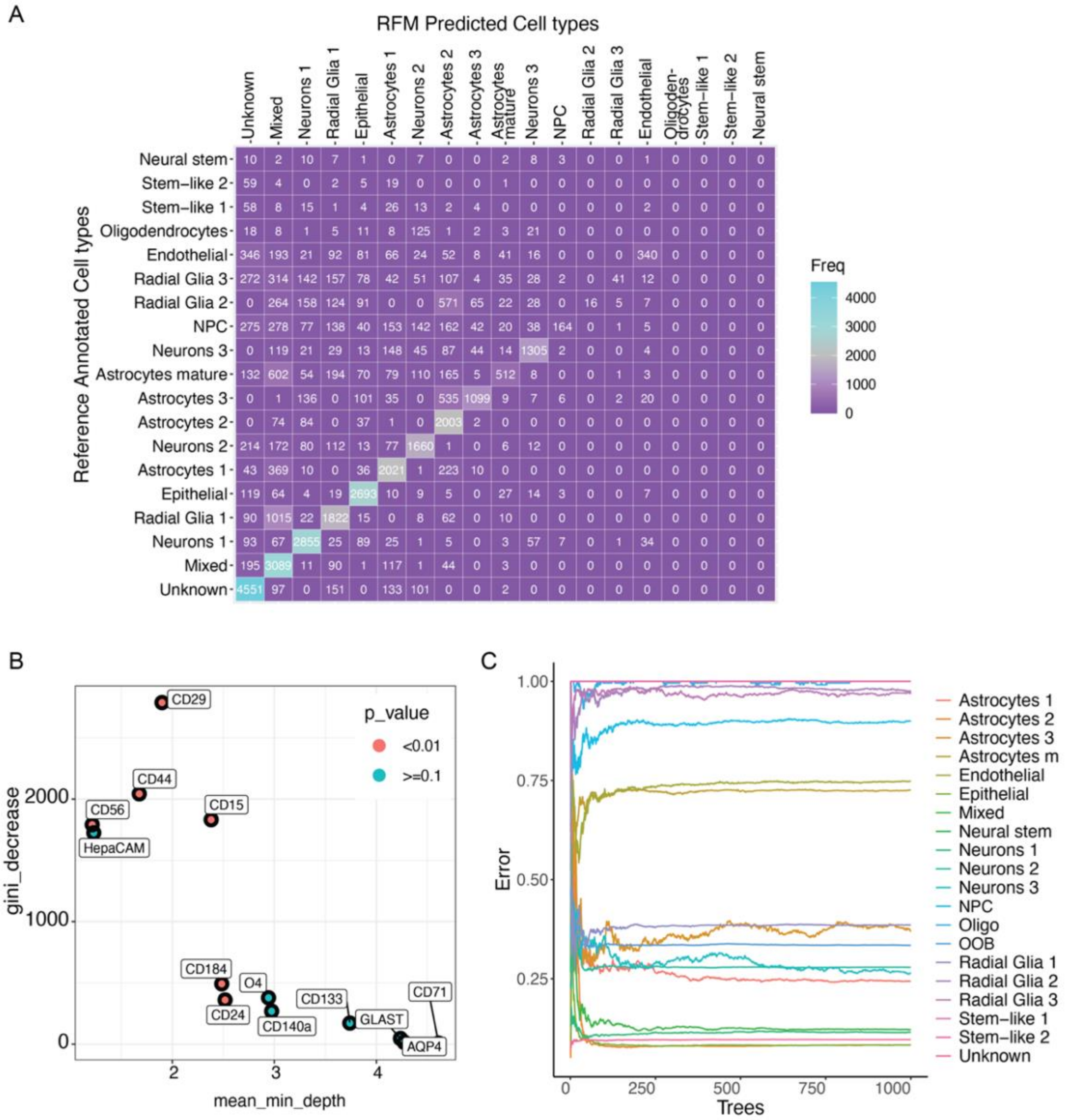

Figure S11: Random Forest Model (RFM) trained with the annotated subset of hMO data. Related to Figure 4 and STAR methods. A) Confusion matrix showing the number of cells predicted to be in each cell type for a hidden group of cells from the annotated data. The y-axis shows the true label annotation in the subset of cells and the x-axis shows the predicted cell labels. When the x and y axis labels match, the cells are correctly predicted. The number of cells predicted are indicated in the squares. The scale also indicates the number of cells in each true label to predicted label match. **B)** MDS plot showing the contribution of antibodies to the prediction in the RFM. High Gini decrease (y-axis) and lower mean minimum depth (x-axis) indicate a greater importance to classification. **C)** Line graph showing the prediction error (y-axis) for different numbers of trees used in training the RFM (x-axis). Coloured lines correspond to each cell type. OOB is the overall error rate. A low error rate indicates a better prediction. Most cell types are predicted accurately, but radial glia2, radial glia3, and oligodendrocytes are not predicted well in the RFM.

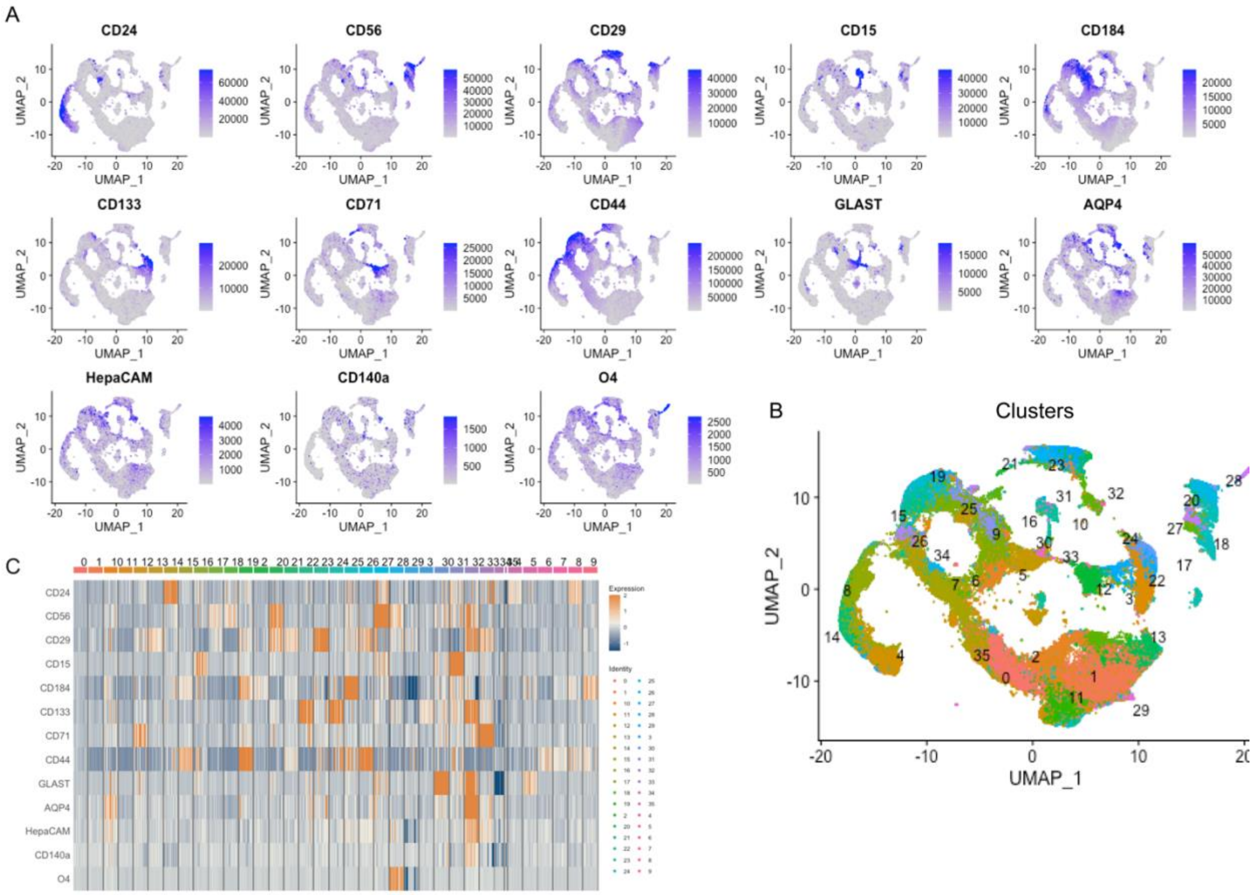

Figure S12: Visualization of protein expression levels in the full nine sample hMO dataset for cell type annotation. Related to Figure 4. **A)** UMAPs of normalized expression for the indicated markers shown as intensity. **B)** UMAP pseudo coloured by cluster, generated by PCA followed by Louvain network detection. Cluster numbers are indicated. **B)** UMAP visualization of Seurat clusters. **C)** Heatmap protein expression per cluster. All cells from each of 9 hMOs from three iPSC lines (AIW002-02, 3450, and AJG001C), two batches (A and B), and acquired on two experiment days (1 = 06/03/2020, 2 = 17/03/2020).

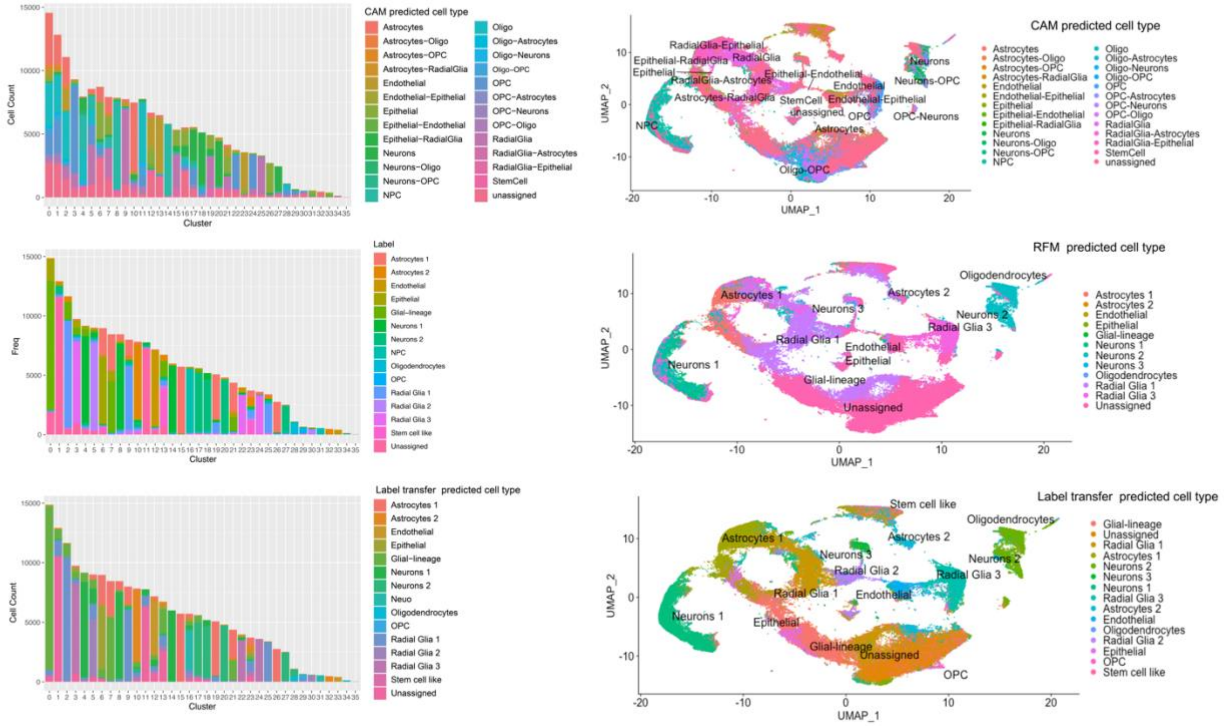

Figure S13: Cell type annotation predictions from CAM, RFM and Seurat label transfer. Related to Figure 4. Left, bar charts with the counts of cells predicated as each cell type in each cluster. Right, UMAPs colours by predicted cell types. Top; CAM predictions with an R threshold for assignment of 0.35 and a double cell type threshold of max R-second max1 of less than 0.01. The results were then filtered to included only predicted cell types with over 200 cells. Middle, RFM predictions from the model trained on the subset of 9000 cells from each of 9 hMOs. Bottom, Seurat label transfer method using the Seurat object from the subset of cells as the reference data. The data shown are all cells from each of 9 hMOs from three iPSC lines (AIW002-02, 3450, and AJG001C), two batches (A and B), and acquired on two experiment days (1 = 06/03/2020, 2 = 17/03/2020).

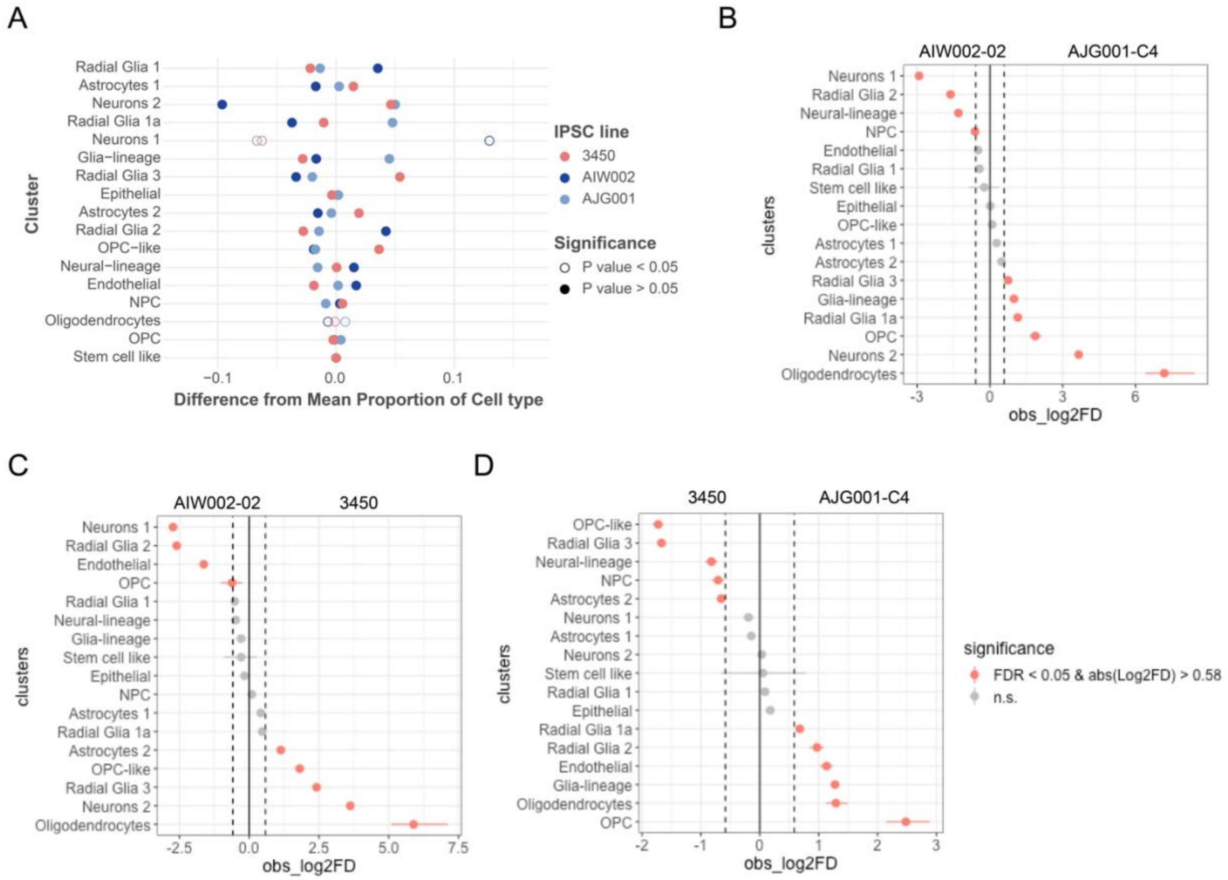

Figure S14: Proportionality test of cell types between iPSC lines using permutation test. Related to Figure 4. **A)** Dot plot showing the results of a permutation ANOVA comparing the proportion of each cell type across all three iPSC lines. The cell types are shown on the y-axis sorted by the mean proportion. The x-axis shows the difference of each observed cell type proportion from the mean proportion of all three lines. The dots for significantly different cell type proportions are shown by outlined circles (as indicated in the legend). **B-D)** Point range plots showing significant differences in proportions of cell types between iPSC lines using a two-condition permutation test. The iPSC lines compared are indicated above the plots. Pink dots indicate a significant difference in the proportion of the indicated cell type between the two iPSC lines with an adjusted p-value (FDR > 0.05) and log fold change in proportion > 0.58, indicated in the legend. The cell types are on the y-axis sorted by log fold change. **B)** AIW002-02 compared to AJG001-C4. **C)** AIW002-02 compared to 3450. **D)** AJG001-C4 compared to 3450. The data shown are all cells from each of 9 hMOs from three iPSC lines (AIW002-02, 3450, and AJG001C), two batches (A and B), and acquired on two experiment days (1 = 06/03/2020, 2 = 17/03/2020).

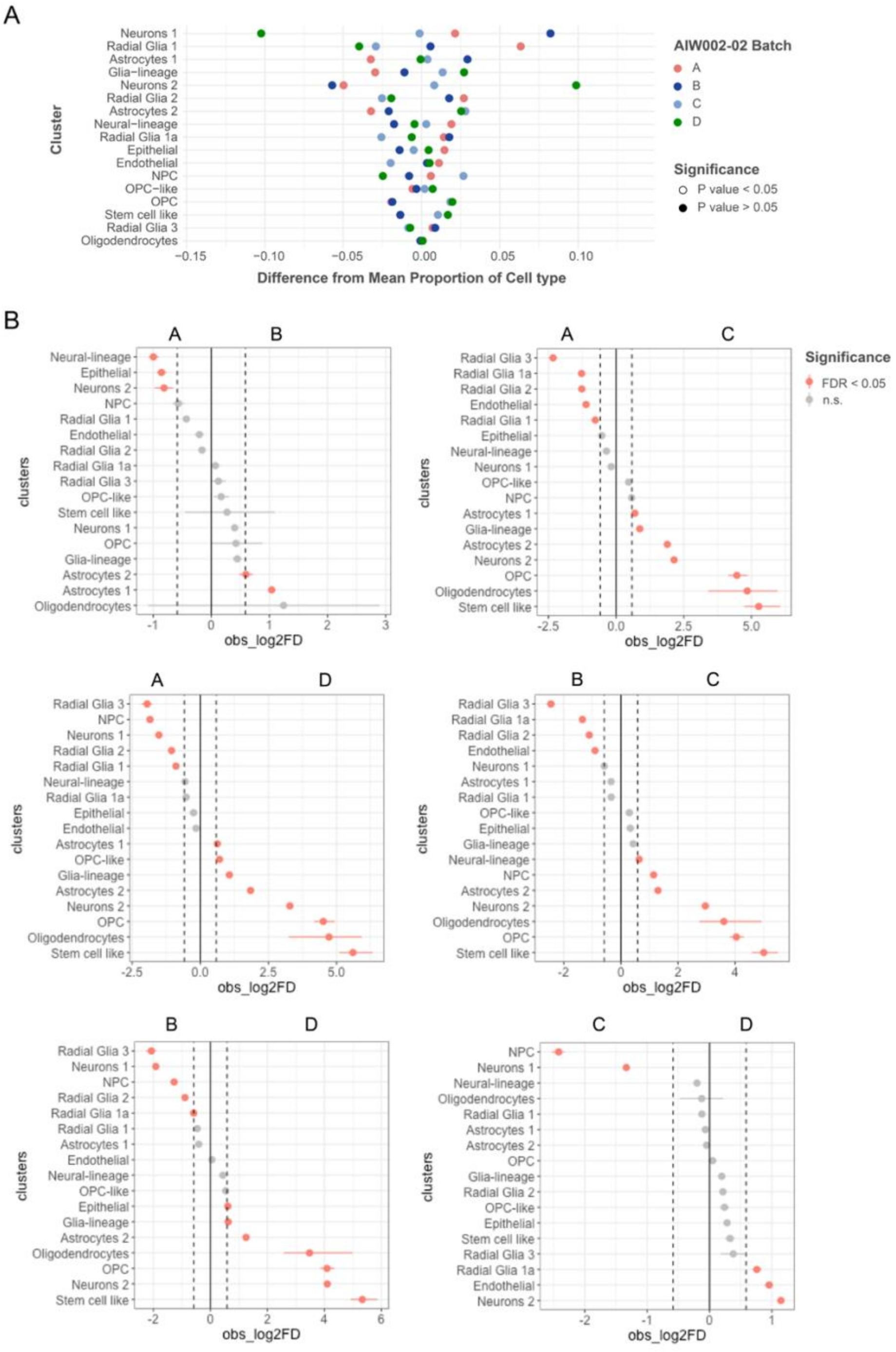

Figure S15: Proportionality test of cell types between AIW002-02 batches. related to Figure 5. A) Dot plot showing the results of a permutation ANOVA comparing the proportion of each cell type across four batches of AIW002-02 hMOs. The cell types are shown on the y-axis sorted by the mean proportion. The x-axis shows the difference of each observed cell type proportion from the mean proportion of all four batches. No significant differences are observed **B)** Point range plots showing significant differences in proportions of cell types between hMO batches using two condition permutation tests. The line contrasts are indicated above the plots. Pink dots indicate a significant difference in the proportion of the indicated cell type between the two batches. Samples are not treated as replicates by the permutation tests. Replicates are as follows Batch A has 2 replicates on separate experiment days (n cells = 35833), Batch B has one replicate (n cells = 53012), Batch C has 5 replicates, 2 replicates on one experiment day and 3 replicates on a second day (n cells = 141940), Batch D has 2 replicates on one experiment day (n cells = 60458).

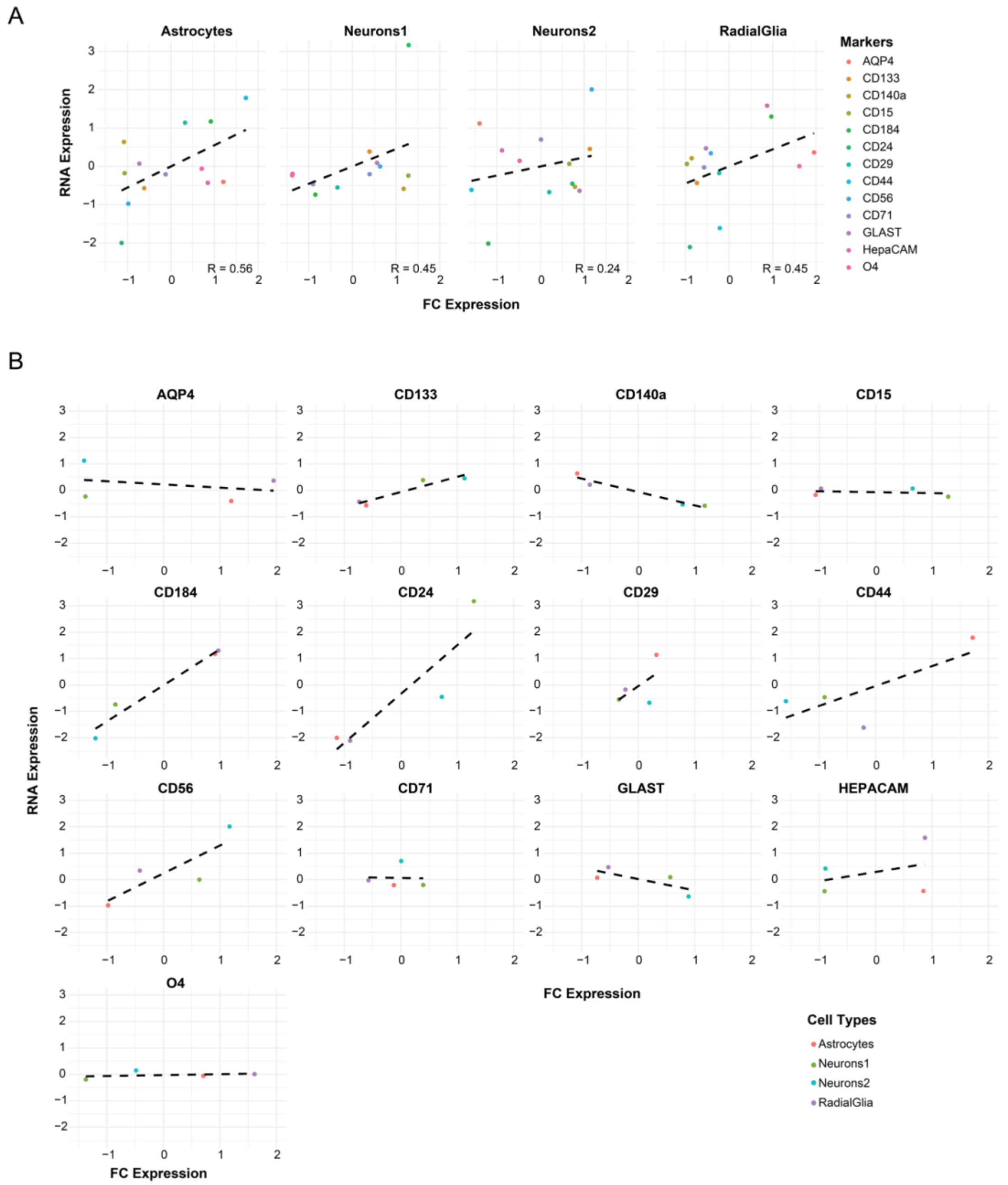

Figure S16: Correlation between scRNAseq and FC marker expression values in four hMO populations sorted populations. Related to Figure 6. A) Scatter plots with FC expression on the x-axis and RNA expression on the y-axis for each marker indicated by colour and split by sorted cell type population. The correlation values are indicated on the plots. The lines are the slot of the correlation coefficients. **B)** Scatter plots with FC expression on the x-axis and RNA expression on the y-axis for each sorted cell type and split by markers. Values are sorted cell population samples split for FC and scRNAseq analysis. The lines are the slot of the correlation coefficient. The sorted populations are from dissociations of AIW002-02 hMOs, 9 months old from one batch labelled with the antibody panel and FC sorted using gates with values determined using *hypergate*.

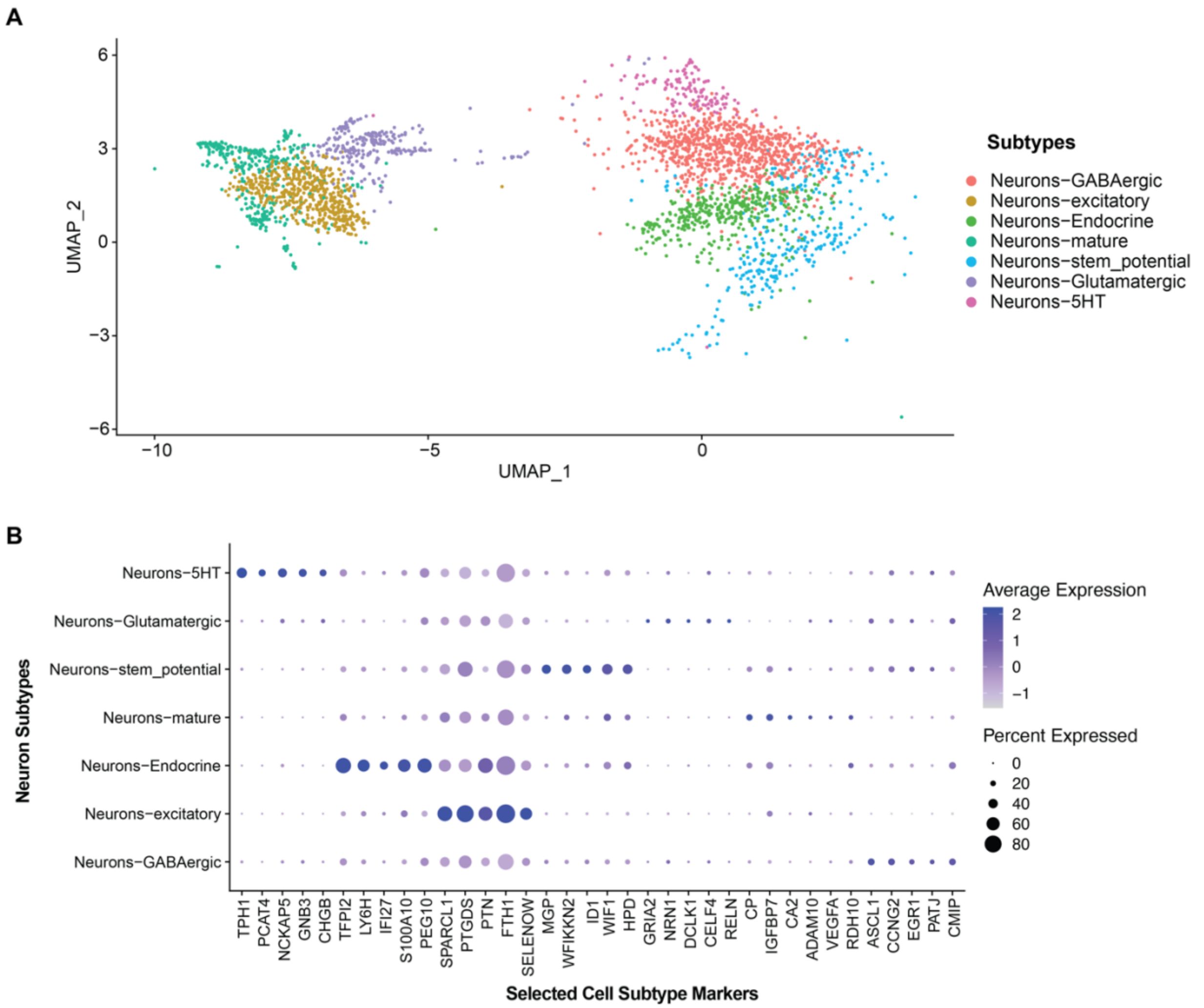

Figure S17: Neuronal subtypes and selected markers from scRNAseq of FACS hMO populations. Related to Figure 6. A) Neurons were subset from the total population and plotted on a UMAP. Subtypes based are differentially expressed genes are indicated in the legend. **B)** Dot plot of 5 selected differentially expressed genes. The proportion of cells in each group expressing a marker is indicated by dot size and the expression level is shown by intensity. Cells are from AIW002-02 hMO batch C. scRNAseq from the four sorted samples were integrated, clustered, and annotated for main cell types. The neuron populations identified by scRNAseq transcriptomes was subset and clustered to identify neuronal subtypes. DGE was calculated between neuron clusters to identify cluster subtype markers.

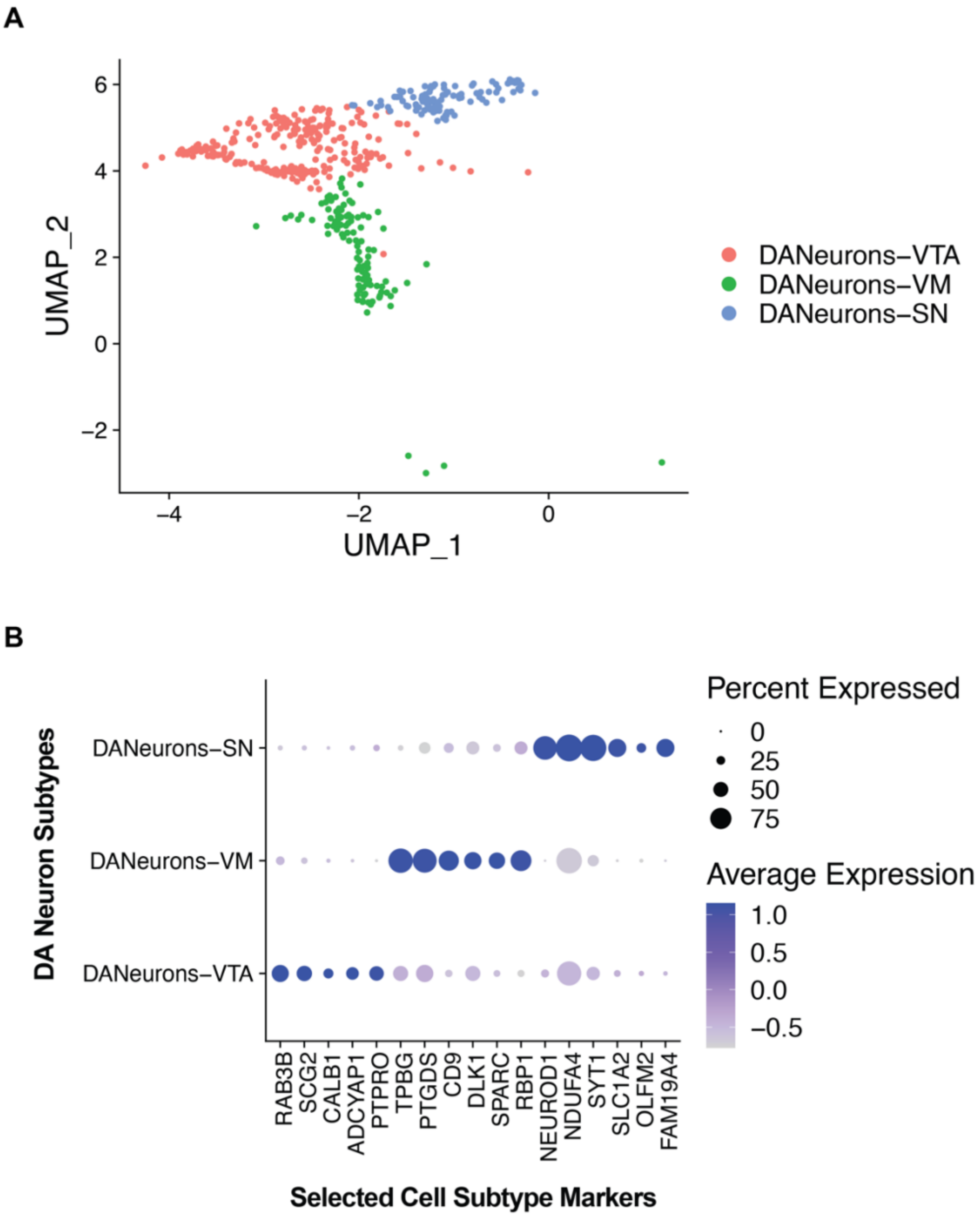

Figure S18: DA neuronal subtypes and selected markers from scRNAseq of FACS hMO populations. Related to Figure 6. A) DA neurons were subset from the total population and plotted on a UMAP. Subtypes were identified by clustering using Louvain network detection. Clusters of DA neuron subtypes were annotated using the markers identified by DGE and analysis of expression of known markers and subtypes DANeurons-VTA (ventral tegmental area), DANeurons-VM (ventral midbrain) and DANeurons-SN (substantia nigra). See Table S13 and S14. **B)** Dot plot of 5 selected differentially expressed genes. The proportion of cells in each group expressing a marker is indicated by dot size and the expression level is shown by intensity. Cells from AIW002-02 hMO batch C. ScRNAseq from the four sorted samples were previously integrated, clustered, and annotated for main cell types.

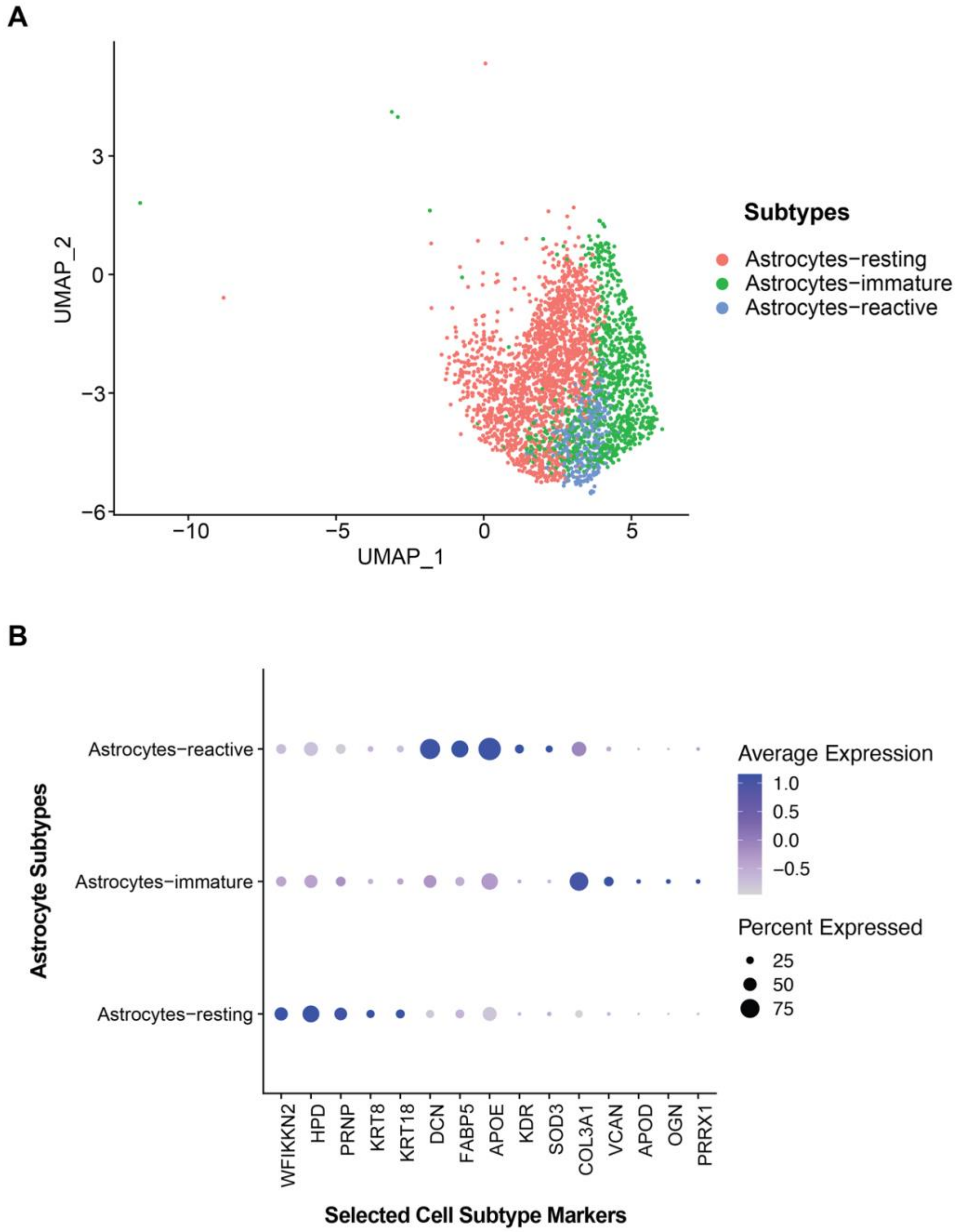

Figure S19: Astrocyte subtypes and selected markers from scRNAseq of FACS hMO populations. Related to Figure 6. A) Astrocytes were subset from the total population and plotted on a UMAP. Subtypes clusters were identified by Louvain network detection and annotated based are differentially expressed genes. **B)** Dot plot of 5 selected differentially expressed genes. The proportion of cells in each group expressing a marker is indicated by dot size and the expression level is shown by intensity. Cells are from AIW002-02 hMO batch C. scRNAseq from the four sorted samples were previously integrated, clustered, and annotated for main cell types.

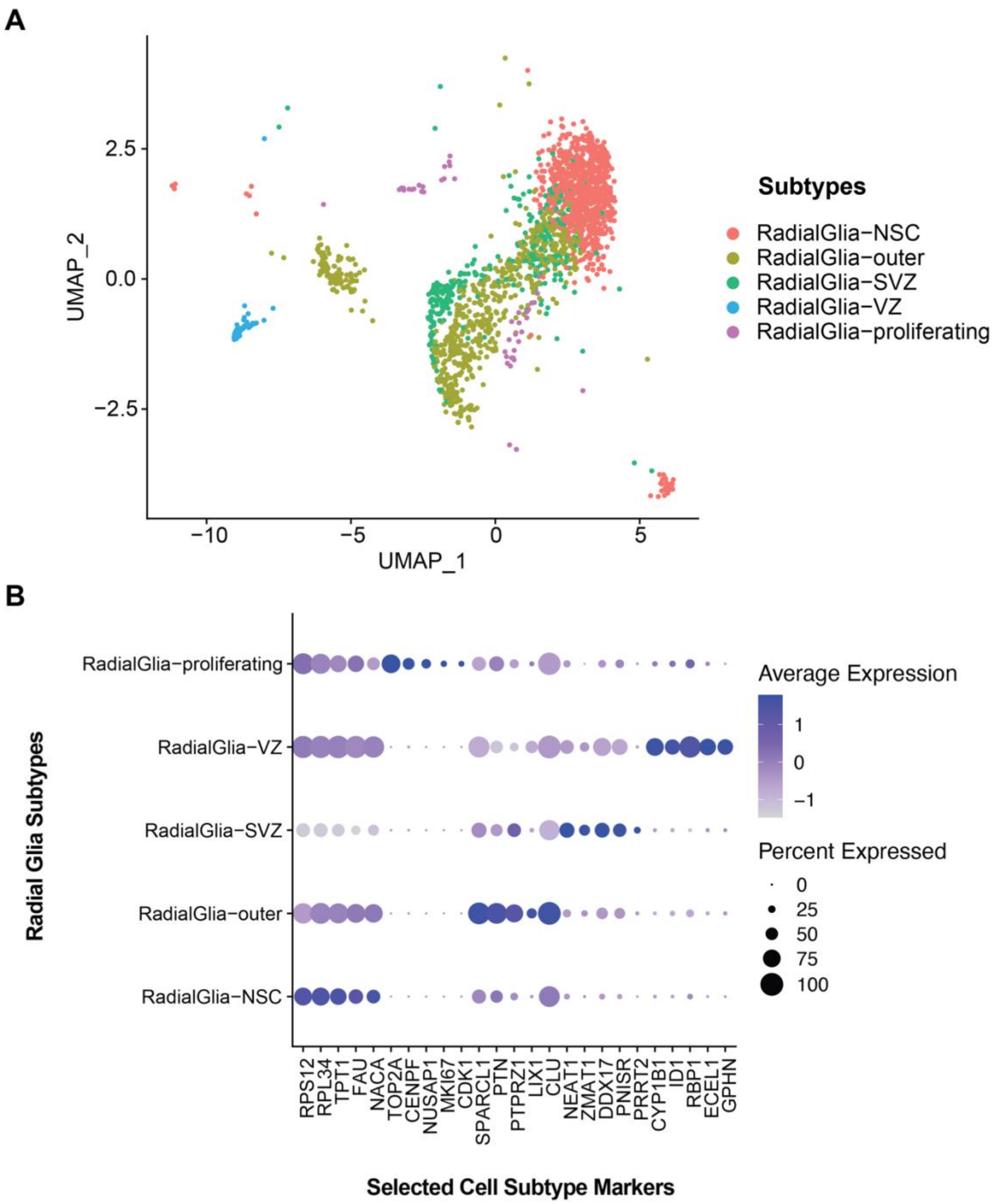

Figure S20: Radial glia subtypes and selected markers from scRNAseq of FACS hMO populations. Related to Figure 6. A) Radial glia cells were subset from the total population and plotted on a UMAP. Subtypes of cells were identified by Louvain network detection and annotated based on differentially expressed genes**. B)** Dot plot of 5 selected differentially expressed genes in each subtype. The proportion of cells in each group expressing a marker is indicated by dot size and the expression level is shown by intensity. Cells are from AIW002-02 hMO batch C. scRNAseq from the four sorted samples were previously integrated, clustered, and annotated for main cell types.

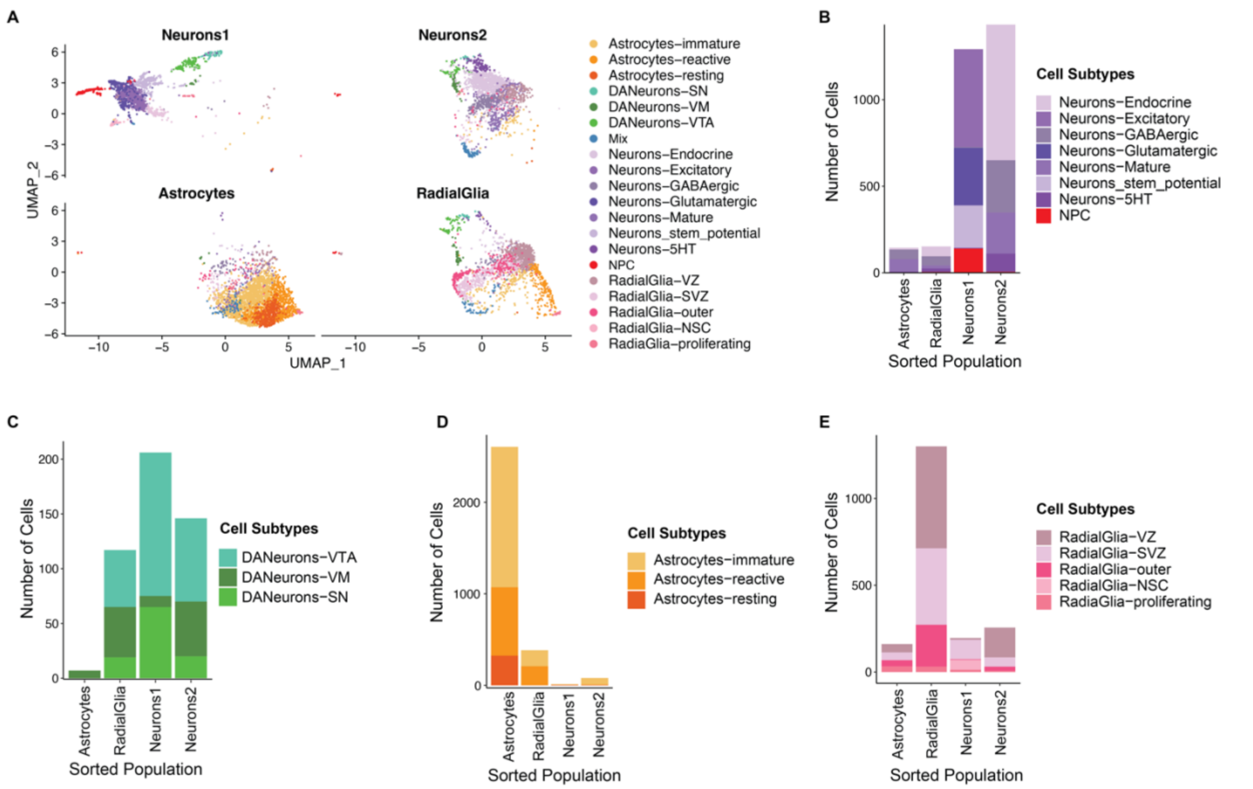

Figure S21: Visualization of the number of cellular subtypes identified by scRNAseq transcriptomes in each FC sorted population. Related to Figure 6. A) UMAP split by sorted population and coloured by cell subtypes. **B)** Stacked bar chart showing the number of each non-DA neuron cell subtype in each sorted population. **C)** Stacked bar chart showing the number of each DA neuron cell subtype in each sorted population. **D)** Stacked bar chart showing the number of each astrocyte cell subtype in each sorted population. **E)** Stacked bar chart showing the number of each radial glia cell subtype in each sorted population. Cells are from AIW002-02 hMO batch C. ScRNAseq from the four sorted samples was integrated, clustered with Louvain network detection, and annotated for main cell types and subtypes.

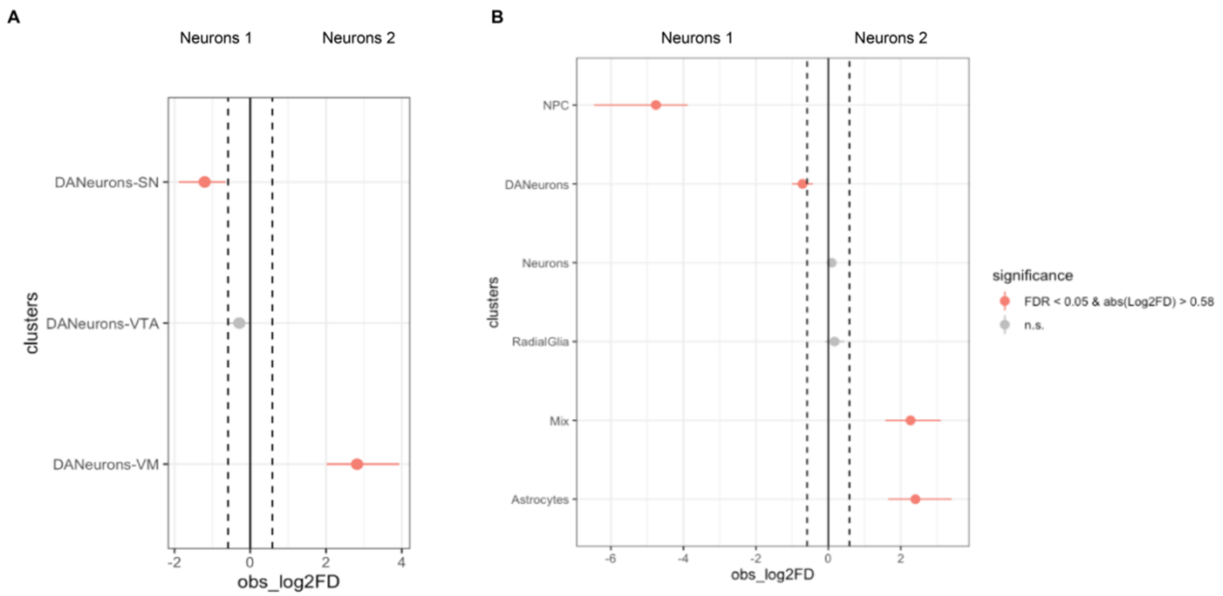

Figure S22: Proportion of cell types and DA neuron subtypes in FACS populations neurons 1 compared to neurons 2. Related to Figure 6. A) Point range plot showing the differences in proportions of DA neuron subtype cells neurons1 and neurons 2. **B)** Point range plot showing the differences in proportions of cell types in neurons1 compared to neurons2. Negative log2FD values indicate a greater proportion of cells in neurons1 and positive log2FD values indicate a greater proportion of cells in neurons2. Pink dots indicate a significant difference.

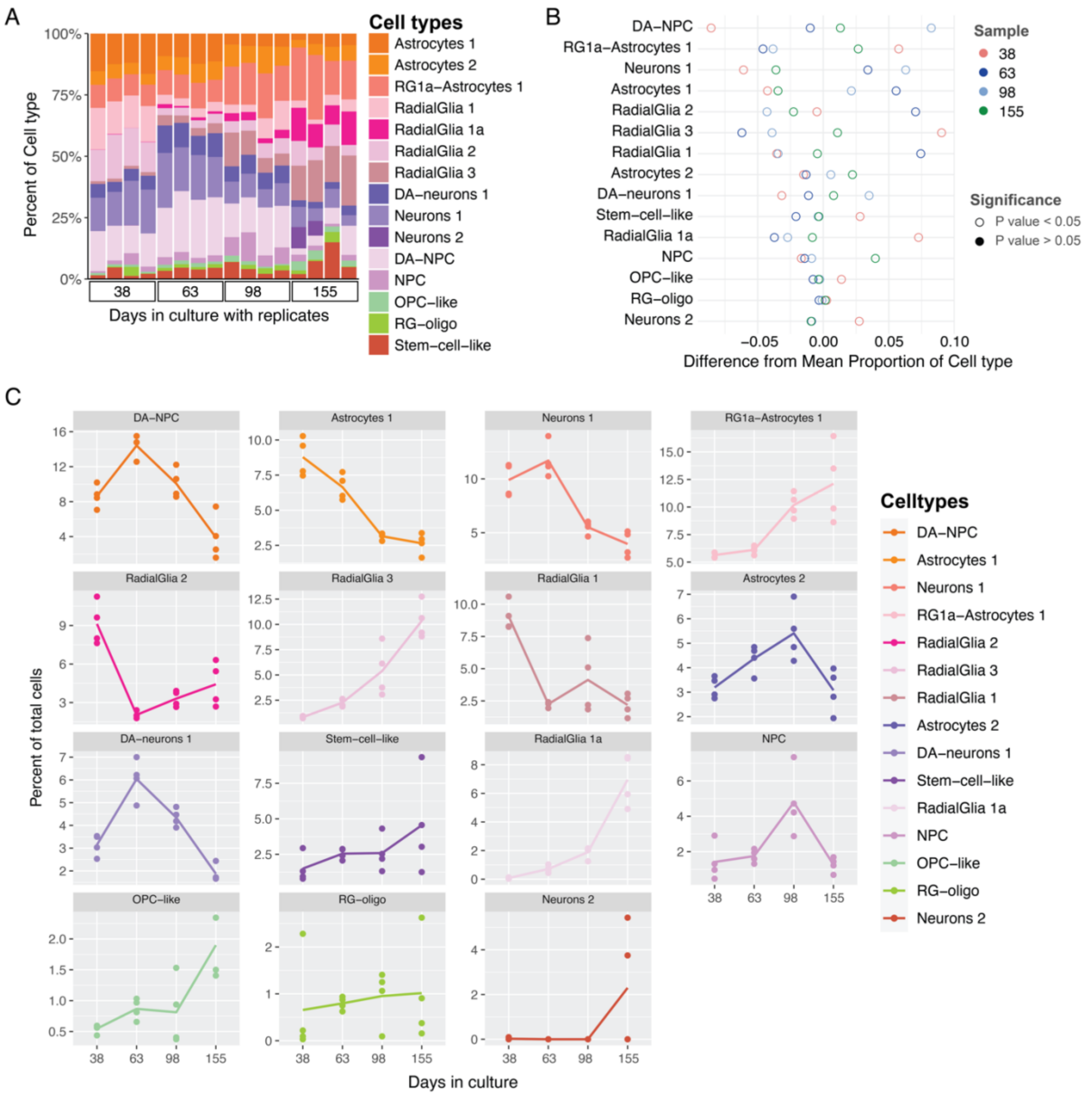

Figure S23: Proportions of cell types in AIW002 over time. Related to Figure 7. A) Dot plot showing the results of a permutation ANOVA comparing the proportion of each cell type across four time points of AIW002-02 hMOs. The cell types are shown on the y-axis sorted by the mean proportion. The x-axis shows the difference of each observed cell type proportion from the mean proportion at each time point. All cell types differ significantly across time. **B)** Bar chart showing proportions of cell types in each sample. Cell types are coloured and shown in the legend. Sample are replicates grouped by the indicated type points. **C)** Line plots showing the proportion of each cell type over time. Dots are shown for each replicate, time points are shown on the x-axis. Each cell type is shown separately. Cell types are indicated in the legend and facet labels above each plot. All samples are AIW002-02 (batch E) with four experimental replicates per time point annotated using the CelltypeR workflow. Cells were down sampled to 2000 cells per sample, n=32000.

Table S1: Sources of expression values used for reference matrices for CAM predictions and scRNAseq cell type annotations.

Expression type	Tissue type	Source/Reference	URL
Protein (FC)	Separate 2D cultures	This work	NA
Total RNAseq	Cell isolated from adult brain	Zhang et al, 2016[1]	https://www.brainrnaseq.org/
scRNAseq	Developing cortex	Nowakowski et al, 2017[2]	https://cortex-dev.cells.ucsc.edu
scRNAseq	Developing forebrain	Van Bruggen et al, 2022[3]	https://human-forebraindev.cells.ucsc.edu
scRNAseq	Fetal midbrain	La Manno et al, 2016[4]	https://www.ncbi.nlm.nih.gov/geo/query/acc.cgi?acc=GSE76381
scRNAseq	Developing midbrain and striatum	Bhaduri et al, 2021[5]	https://dev-brain-regions.cells.ucsc.edu
snRNAseq	Adult midbrain	Kamath et al, 2022[6]	https://singlecell.broadinstitute.org/ single_cell/ study/SCP1768/
scRNAseq	Midbrain organoids	Mohamed et al 2021[7]	https://www.ncbi.nlm.nih.gov/geo/query/acc.cgi?acc=GSE186780
scRNAseq	Cerebral organoids	Tanaka et al, 2020[8]	https://cells.ucsc.edu/?ds=organoidatlas&meta=Cluster

Table S2: Main effect of iPSC line from a 2-way ANOVA and Tukey's posthoc test of the significant differences. Significant differences with a p-value < 0.05 are highlighted in bold. P values are shown for the 2-way ANOVA main effects and the adjusted p Value from the Tukey's HSD post hoc tests.

**Cell type**	**Variable**	**Contrast**	**Test**	**P value**
Neurons 1	IPSC	Main effect	ANOVA 2-way	**0.00046**
Neurons 2	IPSC	Main effect	ANOVA 2-way	**0.00174**
NPC	IPSC	Main effect	ANOVA 2-way	**0.04065**
Oligodendrocytes	IPSC	Main effect	ANOVA 2-way	**0.00127**
OPC-like	IPSC	Main effect	ANOVA 2-way	**0.00177**
Neurons 1	IPSC	AJG001-AIW002	Tukey	**0.00409**
Neurons 1	IPSC	3450-AIW002	Tukey	0.87746
Neurons 1	IPSC	3450-AJG001	Tukey	**0.00085**
NPC	IPSC	AJG001-AIW002	Tukey	0.96515
NPC	IPSC	3450-AIW002	Tukey	**0.05442**
NPC	IPSC	3450-AJG001	Tukey	0.09643
Neurons 2	IPSC	AJG001-AIW002	Tukey	0.76817
Neurons 2	IPSC	3450-AIW002	Tukey	**0.00215**
Neurons 2	IPSC	3450-AJG001	Tukey	**0.01683**
Oligodendrocytes	IPSC	AJG001-AIW002	Tukey	**0.00081**
Oligodendrocytes	IPSC	3450-AIW002	Tukey	0.07856
Oligodendrocytes	IPSC	3450-AJG001	Tukey	0.10891
OPC-like	IPSC	AJG001-AIW002	Tukey	**0.01652**
OPC-like	IPSC	3450-AIW002	Tukey	**0.00222**
OPC-like	IPSC	3450-AJG001	Tukey	0.77824

Table S3: Tukey's HSD test of the interaction effect between iPSC line and protein expression. Showing all significant differences between iPSC pairs for a given protein marker. The significance threshold was considered p value < 0.05. Mean values across cells were taken for each sample in each cell type (n=3). Diff, indicates the difference between the mean values of the 3 replicates.

**Cell type**	**Marker**	**iPSC contrast**	**Diff**	**P value**
Astrocytes 2	AQP4	3450-AIW002	0.98259126	0.00055
NPC	CD15	3450-AIW002	1.32415615	0.00045
Neurons 1	CD24	AJG001-AIW002	1.48879002	0.00000
Neurons 1	CD24	3450-AJG001	-1.3728338	0.00000
Astrocytes 1	CD29	3450-AJG001	1.95363377	0.00000
Astrocytes 1	CD29	3450-AIW002	1.45368611	0.00149
Neurons 2	CD56	3450-AIW002	1.58062883	0.00000
Neurons 2	CD56	3450-AJG001	0.96197282	0.00120
Radial Glia 2	GLAST	3450-AIW002	1.50443557	0.00011

Table S4: *Hypergate* prediction of cell types. Astrocytes 1 and 2 are combined and radial glia 1 and 2 are combined. Neurons1, neurons2, NPCs, and oligodendrocytes are left as separate populations. The rest of the cell types were combined into one group labelled 'other'.

**Cell type**	**Accuracy**	**Gating strategy**
Astrocytes	95.30%	"CD44 >= 69134.93, CD184 >= 4790.36, CD24 <= 14941.93, CD15 <= 21652.39, CD133 <= 16111.91, CD71 <= 24201.84, CD56 <= 31661.17, CD184 <= 41979.12, GLAST >= -1829.09, CD29 >= -221.8, GLAST <= 19910.19, O4 <= 28771.54, CD15 >= -5153.37, O4 >= -13039.53, CD133 >= -5203.24"
Endothelial	97.78%	"CD71 >= 10690.89, CD133 <= 14612.87, CD56 <= 23612.89, CD44 <= 154039.7, CD15 <= 33664.2, CD184 >= -8415.42, O4 <= 6648.85, HepaCAM >= -2266.38, CD184 <= 26695.98, GLAST >= -3953.12, AQP4 <= 179930, GLAST <= 41721.09, CD24 <= 204827.5, CD29 <= 93582.52, CD133 >= -6085.38"
Epithelial	96.14%	"CD44 >= 57643.44, CD184 <= 3973.42, CD24 <= 13819.15, CD133 <= 10956.84, CD15 <= 18368.35, CD44 <= 217613.6, CD184 >= -7614.27, CD29 <= 41852.39, CD71 <= 16803.39, CD56 <= 27459.87, HepaCAM >= -2503.27, GLAST >= -21787.42, O4 <= 13506.06, GLAST <= 7895.25, O4 >= -15995.77, CD71 >= -7398.14, AQP4 >= -27593.11, AQP4 <= 58460.66, CD140a <= 2380.11"
Glial lineage	98.19%	"CD44 >= 18981.62, CD44 <= 67947.27, CD24 <= 8532.04, CD133 <= 6503.12, CD184 >= 671.64, CD184 <= 6027.05, CD56 <= 10803.37, CD15 <= 9272.85, CD71 <= 9899.44, CD29 <= 18512.49, GLAST <= 2972.65, GLAST >= -4303.57, O4 >= -8600.3, AQP4 <= 25934.81, O4 <= 3831.41, AQP4 >= -30283.57, HepaCAM <= 2853.07, CD15 >= -8752.85"
Neural lineage	99.12%	"CD29 <= 5164.46, CD44 <= 16345.29, CD56 <= 8228.51, CD15 <= 10097.88, CD24 <= 14430.59, CD133 <= 4690.34, AQP4 <= 10768.68, CD71 <= 9058.63, CD184 >= -4118.05, CD184 <= 4616.04, GLAST <= 3205.48, GLAST >= -5846.58, HepaCAM >= -2172.31, AQP4 >= -32414.35, O4 <= 7012.81, O4 >= -10615.22, CD15 >= -9709.93"
Neurons 1	97.84%	"CD24 >= 11411.88, CD71 <= 13046.05, CD15 <= 26938.4, CD133 <= 9407.79, CD56 <= 24563.79, HepaCAM >= -2301.21, GLAST <= 7464.28, GLAST >= -10157.93, CD29 >= 388.64, CD29 <= 32358.1, CD184 >= -8356.64, CD184 <= 34967.37, AQP4 <= 54412.99, CD44 <= 330276.01, CD44 >= 370.32, CD71 >= -6552.91, O4 <= 5850.52, AQP4 >= -36288.88, CD140a <= 1853.66"
Neurons 2	97.35%	"CD56 >= 11306.47, O4 <= 6512.56, CD184 <= 5993, CD15 <= 35454.67, CD133 <= 14378.52, CD44 <= 69578.63, CD184 >= -4544.7, HepaCAM <= 2516.97, AQP4 <= 56704.2, O4 >= -3686.94, CD71 <= 37546.04, GLAST >= -8086.99, CD24 <= 89382.14, CD133 >= -6581.01, AQP4 >= -35872.41, CD29 >= -1750.44, CD29 <= 127463.18, CD44 >= -1745.39, GLAST <= 46130.06"
NPC	98.19%	"CD15 >= 16883.48, CD56 <= 68152.13, CD29 <= 32492.33, O4 <= 6603.71, CD133 <= 34264.57, CD71 <= 33362.52, CD44 <= 579529, GLAST >= -8585.92, CD184 >= -8395.97, AQP4 <= 143544.3, CD24 <= 126973.72, CD184 <= 64418.66, HepaCAM <= 8196.44, CD24 >= -10750.26, CD56 >= -863.58, GLAST <= 100720.56, CD140a <= 4207.56, O4 >= -20194.79, CD133 >= -4657.86"
Oligodendrocytes	99.85%	"O4 >= 6954.58, CD71 <= 259264.61, AQP4 <= 251950.1, CD24 <= 79343.15, GLAST >= -12629.13"
OPC	99.14%	"CD184 <= -3881.47, O4 <= 5819.9, HepaCAM <= 2576.93, CD15 <= 20078.87, CD133 <= 24914.78, GLAST <= 25128.87, CD44 <= 169438.5, CD71 <= 47633.15, CD56 <= 70209.7"
OPC-like	98.69%	"CD133 >= 17973.84, HepaCAM <= 7386.68, CD15 <= 93841.76, AQP4 <= 93903.5, O4 <= 19514.81, CD71 <= 171467.93, CD24 <= 186318.34, CD29 <= 75236.89, CD184 >= -18571.9, CD56 <= 102289.4, CD184 <= 66643.74, GLAST <= 54848.83, HepaCAM >= -8102.46"
Radial Glia	91.93%	"CD184 >= 4670.84, CD24 <= 13151.85, CD15 <= 16116.35, CD44 <= 108487.72, AQP4 <= 27956.54, CD133 <= 19864.36, CD71 <= 10261.43, CD29 <= 39977.27, O4 <= 2775.15, GLAST >= -2949.46, CD56 <= 43016.34, O4 >= -6620.26, HepaCAM >= -1840.22, CD56 >= -1630.13, CD71 >= -8436.27, CD44 >= -164.1, AQP4 >= -27462.2, HepaCAM <= 5943.6"
Stem-like	99.95%	"GLAST <= -5784.06, CD133 <= 17757.4, CD184 >= -2183.33, O4 <= 43826.44, CD44 <= 132336.8"

Table S5: Cell counts of the cell types defined by hypergate after manual FlowJo gating and the expected counts from the reference object used to define the gates.

Cell Types	Cell Counts Gated in FlowJo	Cell counts in Reference Object
Astrocytes	23170	29961
Endothelial	6974	7588
Epithelial	9310	8971
Glial_lineage	5185	14865
Neural_lineage	4775	7769
Neurons1	13050	23612
Neurons2	10748	18115
NPC	12998	6265
Oligodendrocytes	1597	1100
OPC	119666	669
OPC-like	19468	7590
RadialGlia	23959	70526
StemCellLike	153114	129

Table S6: Correlation coefficient between protein intensity levels measured by FC and RNA expression measured by scRNAseq. The four FACS sorted populations are indicated. The 13 proteins targeted in the FC antibody panel and the corresponding genes are used as the input.

		scRNAseq			
		Astrocytes	RadialGlia	Neurons1	Neurons2
FC	Astrocytes	0.55533284	0.15228784	-0.4517431	-0.1806433
	RadialGlia	0.13058154	0.44510732	-0.3314321	0.05559489
	Neurons1	-0.4098105	-0.3016957	0.45349104	0.05175022
	Neurons2	-0.4858683	-0.1624758	0.37703856	0.23752196

Table S7: Cell type name with selected cell type markers and which FACS sorted population contains each cell subtype.

Cell subtype	Selected markers	Prevalence in FACS population
Neurons-GABAergic	ASCL1,CCNG2, EGR1, PATJ,CMIP	Neurons2
Neurons-mature	CP,IGFBP7,CA2,ADAM10,VEGFA,RDH10	Neurons1
Neurons-Glutamatergic	GRIA2,NRN1,DCLK1,CELF4,RELN	Neurons1
Neurons-stem-potential	MGP,WFIKKN2,ID1,WIF1,HPD	Neurons2
Neurons-excitatory	SPARCL1,PTGDS,PTN,FTH1,SELENOW	Neurons1
Neurons-endocrine	TFPI2,LY6H,IFI27,S100A10,PEG10	Neurons2
Neurons-5HT	TPH1,PCAT4,NCKAP5,GNB3,CHGB	Neurons2
DANeurons-VTA	RASGRP1,SCG2,RAB3B,CALB1,ADCYA	Neurons1 and Neurons 2
DANeurons-VM	TPGB, HES1, IFITM2, PTGDS	Neurons2
DANeurons-SN	TTR, SYT1, PCDH9, SLC1A2, OLFM3	Neurons1
Astrocytes-immature	COL3A1,VCAN, APOD, OGN, PRRX1	Astrocytes and RG
Astroctyes-reactive	DNC, FABP5, APOE, S100A6, KDR	Least abundant
Astrocytes-resting	HPD,WFIKKN2,PRNP,KRT8,KRT18,	Most abundant
RadialGlia-VZ	CYP1B1,ID1, RBP1, ECEL1, GPHN	Neurons 1
RadialGlia-SVZ	NEAT, ZMAT1, DDX17, PNISR, PRRT2	RadialGlia
RadialGlia-outer	PTN, PTPRZ, SPARCL1, LIX1	RadialGlia
RadialGlial-NSC	TPT1,FAU,NACA,RPL34,RPS12	RadialGlia
RadialGlia-proliferating	TOP2A,MKI67,CDK1,NUSAP1,CENPF	Neurons1 and Astrocytes

Table S8: References of DA neuron subtypes for selected cluster markers.

Subgroup Cluster	Marker gene	Region or subtype indicated	Reference
DA1<br><br>DANeurons-VTA	RAB3B	VM DA maturation	Monzon-Sandoval 2020[9]
	SCG2	VTA	Wen 2021[10], Greene 2015[11]
	CALB1	VTA	Chung 2005[12], Greene 2005[11]
	RASGRP1	VM	Eshraghi 2020[13]
	STMN2	VM DA development	Yin 2009[14], Fernandes 2020[15]
	ADCYAP1	VTA	Chung 2005[12], Greene 2005[11]
	PTPRO	VM DA maturation	Xu 2022[16]
DA2<br><br>DANeurons-VM	TPBG	VM DA maturation	Yoo 2021[17]
	PTGDS	VM	Zeisel 2018[18]
	CD9	VTA	Li 2014[19]
	DLK1	DA neurons	Birtele 2022[20]
	SPARC	SN	Monzon-Sandoval 2020[9]
	RBP1	VM	Veenvliet 2013[21]
	HES1	VM	Kameda 2011[22], Hegarty 2013[23]
DA3<br><br>DANeurons-SN	TTR	DA neurons	Kim 2021[24]
	NEUROD1	DA neurons	Earley 2021[25], Termine 2022[26]
	NDUFA4	DA neurons	Fernandes 2020[15]
	SYT1	SN	Poulin 2014[27]
	SLC1A2	SN	Zhang 2020[28]
	OLFM2	SN	Bosser 2009[29], Yang 2022[30]
	FAM19A4	SN	Li 2016[31] , Kamath 2022[6]

Table S9: Annotation of DA subtypes in scRNAseq data from different references.

Source	DA1 - VTA	DA2 - VM	DA3 - SN
Kamath 2022[6] DA subtypes of SN (Human)	SOX6_DDT	CALB1_RBP4	CALB1_CALCR (not a good fit)<br>Should be FAM19A4
Poulin 2014[27] DA subtypes (Mouse)	DA2B (VTA)	DA1A (SN)	Maybe DA2C<br>OTX2 and SLC17A6 (VGLUT2).
Poulin 2014[27] VTA vs SN list	Both – more VTA	Both	Both – RAB3C SN and OTX2 VTA
Tiklova 2019[32]<br>Gene expression in shiny app<br>(Mouse)	VT-Dat-high (doral VTA/PAG)	VT-Dat-high (doral VTA/PAG)	AT-Dat-high (SN), N-Dat-low (not matched to a region)
Tiklova 2019[32]<br>From marker list<br>(Mouse)	AT-Dat (SN), VT-Dat (dorsal VTA/PAG), T-Dat (VTA some SN)	AT-Dat (SN) (possible strongest match), VT-Dat (dorsal VTA/PAG), T-Dat (VTA some SN)	AT-Dat, VT-Dat, T-Dat also G-dat-low, GT-Dat-low (VTA)
Aguila 2022[33]<br>SN vs VTA<br>(Human)	VTA	VM	SN
Cluster markers (Table 13)	Ventral Midbrain possible VTA	Ventral midbrain both SN and VTA	Possible SN
Overall	VTA	VM	SN

Table S10: Proportions of cell types and cell subtypes in four FACS sorted populations from scRNAseq transcriptomics.

Cell types	Neurons 1	Neurons 2	Astrocytes	Radial Glia
Astrocytes	1%	4%	87%	19%
DANeurons	12%	7%	0%	6%
Neurons	67%	71%	5%	7%
NPC	8%	0%	0%	0%
All Neuronal Cells	87%	79%	5%	13%
RadialGlia	11%	13%	5%	65%
Mix	1%	4%	3%	2%
**Cell subtype**	**Neurons 1**	**Neurons 2**	**Astrocytes**	**Radial Glia**
Astrocytes-immature	0%	4%	51%	9%
Astrocytes-reactive	0%	0%	25%	10%
Astrocytes-resting	0%	0%	11%	0%
DANeurons-SN	4%	1%	0%	1%
DANeurons-VM	1%	3%	0%	2%
DANeurons-VTA	8%	4%	0%	3%
Mix	1%	4%	3%	2%
Neurons_stem_potential	14%	0%	0%	0%
Neurons-5HT	0%	5%	0%	1%
Neurons-Endocrine	0%	39%	0%	3%
Neurons-Excitatory	33%	0%	0%	0%
Neurons-GABAergic	0%	15%	2%	3%
Neurons-Glutamatergic	19%	0%	0%	0%
Neurons-Mature	0%	12%	2%	1%
NPC	8%	0%	0%	0%
RadiaGlia-proliferating	1%	0%	1%	2%
RadialGlia-NSC	3%	0%	0%	0%
RadialGlia-outer	0%	1%	1%	12%
RadialGlia-SVZ	7%	3%	2%	22%
RadialGlia-VZ	1%	9%	2%	29%

Table S11: Antibody panel used for time course analysis with cell types previously reported to be identified by each marker.

Antibody/Marker	Protein/Gene	Reported Cell type marker	References
CD24	CD24	Neurons and neural stem cells<br>Cancer stem cells	Uchida 2000,[34] Pruszak 2007,[35] Pruszak 2009,[36] Sundberg 2009,[37] Yuan 2011,[38] Wang 2013[39]
CD56	NCAM1	Neurons and neural stem cells<br>Cancer cells	Pruszak 2007,[35] Pruszak 2009,[36] Sundberg 2009[37]
CD29	ITGB1	Stem cell	Pruszak[35], Yuan 2011[38],
CD15	FUT4	Neural precursor	Pruszak 2007,[35] Pruszak 2009,[36] Yuan 2011,[38] Sandor 2017[40]
CD184	CXCR4	Neural stem cell	Yuan 2011,[38] Sandor 2017[40]
CD133	PROM1	Stem cell	Uchida 2000,[34] Pruszak 2007,[35] Barraud 2007[41], Pruszak 2009,[36]
CD44	CD44	Glia	Liu 2004,[42] Yuan 2011,[38]
CD140a	PDGFRA	OPC	Liu 2004,[42] Wang 2013[39]
TH	TH	Dopaminergic neurons and lineage	Wolf 1989,[43] Kan 2007[44]
SSEA4	Carbohydrate epitope	Neural stem cell, stem cell	Henderson, 2002,[45] Barraud 2007,[41] Pruszak 2007,[35] Abujarour 2013[46]
CD49f	ITA6/ITGA6	Activated astrocytes	Barbar 2020[47]

Table S12: ThermoFisher's Attune NxT optical path configuration.

Laser	Wavelength (nm)	Detector	Dichroic mirror	Filter (Band pass)
Violet	405	VL6	740 LP	780/60 BP
		VL5	680-740	710/50 BP
		VL4	635-680	660/20 BP
		VL3	555-635	610/20 BP
		VL2	495-555	525/50 BP
		VL1	417-495	450/40 BP
Blue	488	BL2	555 LP	695/40 BP
		BL1	503-555	530/30 BP
		SSC		488/10 BP
Yellow-Green	561	YL3	650 LP	780/60 BP
		YL2	600-650	620/15 BP
		YL1	577-600	585/16 BP
Red	640	RL3	740 LP	780/60 BP
		RL2	690-740	720/30 BP
		RL1	654-690	670/14 BP

Table S13: BD's FACSAria Fusion optical path configuration.

Laser	Wavelength (nm)	Detector	Dichroic mirror (Long pass)	Filter (Band pass)
Violet	405	A	750 LP	780/60 BP
		B	690 LP	710/50 BP
		C	630 LP	660/20 BP
		D	595 LP	610/20 BP
		E	505 LP	525/50 BP
		F		450/50 BP
Blue	488	A	655 LP	695/40 BP
		B	502 LP	530/30 BP
		C		488/10 BP
Yellow-Green	561	A	735 LP	780/60 BP
		B	685 LP	710/50 BP
		C	630 LP	670/14 BP
		D	600 LP	610/20 BP
		E		582/15 BP
Red	640	A	755 LP	780/60 BP
		B	690 LP	730/45 BP
		C		670/30 BP

References

1. Zhang, Y., Sloan, S.A., Clarke, L.E., Caneda, C., Plaza, C.A., Blumenthal, P.D., Vogel, H., Steinberg, G.K., Edwards, M.S., and Li, G. (2016). Purification and characterization of progenitor and mature human astrocytes reveals transcriptional and functional differences with mouse. Neuron *89*, 37–53.

2. Nowakowski, T.J., Bhaduri, A., Pollen, A.A., Alvarado, B., Mostajo-Radji, M.A., Di Lullo, E., Haeussler, M., Sandoval-Espinosa, C., Liu, S.J., and Velmeshev, D. (2017). Spatiotemporal gene expression trajectories reveal developmental hierarchies of the human cortex. Science *358*, 1318–1323.

3. van Bruggen, D., Pohl, F., Langseth, C.M., Kukanja, P., Lee, H., Albiach, A.M., Kabbe, M., Meijer, M., Linnarsson, S., and Hilscher, M.M. (2022). Developmental landscape of human forebrain at a single-cell level identifies early waves of oligodendrogenesis. Dev. Cell *57*, 1421–1436.

4. La Manno, G., Gyllborg, D., Codeluppi, S., Nishimura, K., Salto, C., Zeisel, A., Borm, L.E., Stott, S.R., Toledo, E.M., and Villaescusa, J.C. (2016). Molecular diversity of midbrain development in mouse, human, and stem cells. Cell *167*, 566–580.

5. Bhaduri, A., Sandoval-Espinosa, C., Otero-Garcia, M., Oh, I., Yin, R., Eze, U.C., Nowakowski, T.J., and Kriegstein, A.R. (2021). An atlas of cortical arealization identifies dynamic molecular signatures. Nature *598*, 200–204.

6. Kamath, T., Abdulraouf, A., Burris, S.J., Langlieb, J., Gazestani, V., Nadaf, N.M., Balderrama, K., Vanderburg, C., and Macosko, E.Z. (2022). Single-cell genomic profiling of human dopamine neurons identifies a population that selectively degenerates in Parkinson's disease. Nat. Neurosci. *25*, 588–595.

7. Mohamed, N.-V., Sirois, J., Ramamurthy, J., Mathur, M., Lépine, P., Deneault, E., Maussion, G., Nicouleau, M., Chen, C.X.-Q., Abdian, N., et al. (2021). Midbrain organoids with an *SNCA* gene triplication model key features of synucleinopathy. Brain Commun. *3*, fcab223. https://doi.org/10.1093/braincomms/fcab223.

8. Tanaka, Y., Cakir, B., Xiang, Y., Sullivan, G.J., and Park, I.-H. (2020). Synthetic analyses of single-cell transcriptomes from multiple brain organoids and fetal brain. Cell Rep. *30*, 1682–1689.

9. Monzón-Sandoval, J., Poggiolini, I., Ilmer, T., Wade-Martins, R., Webber, C., and Parkkinen, L. (2020). Human-Specific Transcriptome of Ventral and Dorsal Midbrain Dopamine Neurons. Ann. Neurol. *87*, 853–868. https://doi.org/10.1002/ana.25719.

10. Wen, G., Pang, H., Wu, X., Jiang, E., Zhang, X., and Zhan, X. (2021). Proteomic characterization of secretory granules in dopaminergic neurons indicates chromogranin/secretogranin-mediated protein processing impairment in Parkinson's disease. Aging *13*, 20335.

11. Greene, J.G., Dingledine, R., and Greenamyre, J.T. (2005). Gene expression profiling of rat midbrain dopamine neurons: implications for selective vulnerability in parkinsonism. Neurobiol. Dis. *18*, 19–31.

12. Chung, C.Y., Seo, H., Sonntag, K.C., Brooks, A., Lin, L., and Isacson, O. (2005). Cell type-specific gene expression of midbrain dopaminergic neurons reveals molecules involved in their vulnerability and protection. Hum. Mol. Genet. *14*, 1709–1725.

13. Eshraghi, M., Ramírez-Jarquín, U.N., Shahani, N., Nuzzo, T., De Rosa, A., Swarnkar, S., Galli, N., Rivera, O., Tsaprailis, G., and Scharager-Tapia, C. (2020). RasGRP1 is a causal factor in the development of l-DOPA–induced dyskinesia in Parkinson's disease. Sci. Adv. *6*, eaaz7001.

14. Yin, M., Liu, S., Yin, Y., Li, S., Li, Z., Wu, X., Zhang, B., Ang, S.-L., Ding, Y., and Zhou, J. (2009). Ventral mesencephalon-enriched genes that regulate the development of dopaminergic neurons in vivo. J. Neurosci. *29*, 5170–5182.

15. Fernandes, H.J.R., Patikas, N., Foskolou, S., Field, S.F., Park, J.-E., Byrne, M.L., Bassett, A.R., and Metzakopian, E. (2020). Single-Cell Transcriptomics of Parkinson's Disease Human In Vitro Models Reveals Dopamine Neuron-Specific Stress Responses. Cell Rep. *33*, 108263. https://doi.org/10.1016/j.celrep.2020.108263.

16. Xu, P., He, H., Gao, Q., Zhou, Y., Wu, Z., Zhang, X., Sun, L., Hu, G., Guan, Q., and You, Z. (2022). Human midbrain dopaminergic neuronal differentiation markers predict cell therapy outcomes in a Parkinson's disease model. J. Clin. Invest. *132*.

17. Yoo, J.-E., Lee, D.R., Park, S., Shin, H.-R., Lee, K.G., Kim, D.-S., Jo, M.-Y., Eom, J.-H., Cho, M.S., and Hwang, D.-Y. (2021). Trophoblast glycoprotein is a marker for efficient sorting of ventral mesencephalic dopaminergic precursors derived from human pluripotent stem cells. Npj Park. Dis. *7*, 1–11.

18. Zeisel, A., Hochgerner, H., Lönnerberg, P., Johnsson, A., Memic, F., Van Der Zwan, J., Häring, M., Braun, E., Borm, L.E., and La Manno, G. (2018). Molecular architecture of the mouse nervous system. Cell *174*, 999–1014.

19. Li, M.D., Burns, T.C., Morgan, A.A., and Khatri, P. (2014). Integrated multi-cohort transcriptional meta-analysis of neurodegenerative diseases. Acta Neuropathol. Commun. *2*, 1–23.

20. Birtele, M., Storm, P., Sharma, Y., Kajtez, J., Wahlestedt, J.N., Sozzi, E., Nilsson, F., Stott, S., He, X.L., and Mattsson, B. (2022). Single-cell transcriptional and functional analysis of dopaminergic neurons in organoid-like cultures derived from human fetal midbrain. Development *149*, dev200504.

21. Veenvliet, J.V., Dos Santos, M.T.A., Kouwenhoven, W.M., Von Oerthel, L., Lim, J.L., Van Der Linden, A.J., Koerkamp, M.J.G., Holstege, F.C., and Smidt, M.P. (2013). Specification of dopaminergic subsets involves interplay of En1 and Pitx3. Development *140*, 3373–3384.

22. Kameda, Y., Saitoh, T., and Fujimura, T. (2011). Hes1 regulates the number and anterior–posterior patterning of mesencephalic dopaminergic neurons at the mid/hindbrain boundary (isthmus). Dev. Biol. *358*, 91–101.

23. Hegarty, S.V., Sullivan, A.M., and O'keeffe, G.W. (2013). Midbrain dopaminergic neurons: a review of the molecular circuitry that regulates their development. Dev. Biol. *379*, 123–138.

24. Kim, T.W., Piao, J., Koo, S.Y., Kriks, S., Chung, S.Y., Betel, D., Socci, N.D., Choi, S.J., Zabierowski, S., and Dubose, B.N. (2021). Biphasic activation of WNT signaling facilitates the derivation of midbrain dopamine neurons from hESCs for translational use. Cell Stem Cell *28*, 343–355.

25. Earley, A.M., Burbulla, L.F., Krainc, D., and Awatramani, R. (2021). Identification of ASCL1 as a determinant for human iPSC-derived dopaminergic neurons. Sci. Rep. *11*, 1–13.

26. Termine, A., Fabrizio, C., Strafella, C., Caputo, V., Petrosini, L., Caltagirone, C., Cascella, R., and Giardina, E. (2022). A Hybrid Machine Learning and Network Analysis Approach Reveals Two Parkinson's Disease Subtypes from 115 RNA-Seq Post-Mortem Brain Samples. Int. J. Mol. Sci. *23*, 2557.

27. Poulin, J.-F., Zou, J., Drouin-Ouellet, J., Kim, K.-Y.A., Cicchetti, F., and Awatramani, R.B. (2014). Defining midbrain dopaminergic neuron diversity by single-cell gene expression profiling. Cell Rep. *9*, 930–943.

28. Zhang, Y., Meng, X., Jiao, Z., Liu, Y., Zhang, X., and Qu, S. (2020). Generation of a novel mouse model of Parkinson's disease via targeted knockdown of glutamate transporter GLT-1 in the substantia nigra. ACS Chem. Neurosci. *11*, 406–417.

29. Bossers, K., Meerhoff, G., Balesar, R., Van Dongen, J.W., Kruse, C.G., Swaab, D.F., and Verhaagen, J. (2009). Analysis of gene expression in Parkinson's disease: possible involvement of neurotrophic support and axon guidance in dopaminergic cell death. Brain Pathol. *19*, 91–107.

30. Yang, Y., Huang, X., Wang, C., and Wang, Y. (2022). Identification of hub genes of Parkinson's disease through bioinformatics analysis. Front. Neurosci., 1709.

31. Li, C.-L., Li, K.-C., Wu, D., Chen, Y., Luo, H., Zhao, J.-R., Wang, S.-S., Sun, M.-M., Lu, Y.-J., and Zhong, Y.-Q. (2016). Somatosensory neuron types identified by high-coverage single-cell RNA-sequencing and functional heterogeneity. Cell Res. *26*, 83–102.

32. Tiklová, K., Björklund, Å.K., Lahti, L., Fiorenzano, A., Nolbrant, S., Gillberg, L., Volakakis, N., Yokota, C., Hilscher, M.M., and Hauling, T. (2019). Single-cell RNA sequencing reveals midbrain dopamine neuron diversity emerging during mouse brain development. Nat. Commun. *10*, 1–12.

33. Aguila, J., Cheng, S., Kee, N., Cao, M., Wang, M., Deng, Q., and Hedlund, E. (2021). Spatial RNA sequencing identifies robust markers of vulnerable and resistant human

midbrain dopamine neurons and their expression in Parkinson’s disease. Front. Mol. Neurosci. *14*, 699562.

34. Uchida, N., Buck, D.W., He, D., Reitsma, M.J., Masek, M., Phan, T.V., Tsukamoto, A.S., Gage, F.H., and Weissman, I.L. (2000). Direct isolation of human central nervous system stem cells. Proc. Natl. Acad. Sci. *97*, 14720–14725.

35. Pruszak, J., Sonntag, K.-C., Aung, M.H., Sanchez-Pernaute, R., and Isacson, O. (2007). Markers and methods for cell sorting of human embryonic stem cell-derived neural cell populations. Stem Cells *25*, 2257–2268.

36. Pruszak, J., Ludwig, W., Blak, A., Alavian, K., and Isacson, O. (2009). CD15, CD24, and CD29 define a surface biomarker code for neural lineage differentiation of stem cells. Stem Cells *27*, 2928–2940.

37. Sundberg, M., Jansson, L., Ketolainen, J., Pihlajamäki, H., Suuronen, R., Skottman, H., Inzunza, J., Hovatta, O., and Narkilahti, S. (2009). CD marker expression profiles of human embryonic stem cells and their neural derivatives, determined using flow-cytometric analysis, reveal a novel CD marker for exclusion of pluripotent stem cells. Stem Cell Res. *2*, 113–124.

38. Yuan, S.H., Martin, J., Elia, J., Flippin, J., Paramban, R.I., Hefferan, M.P., Vidal, J.G., Mu, Y., Killian, R.L., and Israel, M.A. (2011). Cell-surface marker signatures for the isolation of neural stem cells, glia and neurons derived from human pluripotent stem cells. PloS One *6*, e17540.

39. Wang, J., O’Bara, M.A., Pol, S.U., and Sim, F.J. (2013). CD133/CD140a-based isolation of distinct human multipotent neural progenitor cells and oligodendrocyte progenitor cells. Stem Cells Dev. *22*, 2121–2131.

40. Sandor, C., Robertson, P., Lang, C., Heger, A., Booth, H., Vowles, J., Witty, L., Bowden, R., Hu, M., and Cowley, S.A. (2017). Transcriptomic profiling of purified patient-derived dopamine neurons identifies convergent perturbations and therapeutics for Parkinson’s disease. Hum. Mol. Genet. *26*, 552–566.

41. Barraud, P., Stott, S., Møllgård, K., Parmar, M., and Björklund, A. (2007). In vitro characterization of a human neural progenitor cell coexpressing SSEA4 and CD133. J. Neurosci. Res. *85*, 250–259.

42. Liu, Y., Han, S.S., Wu, Y., Tuohy, T.M., Xue, H., Cai, J., Back, S.A., Sherman, L.S., Fischer, I., and Rao, M.S. (2004). CD44 expression identifies astrocyte-restricted precursor cells. Dev. Biol. *276*, 31–46.

43. Wolf, M.E., Zigmond, M.J., and Kapatos, G. (1989). Tyrosine hydroxylase content of residual striatal dopamine nerve terminals following 6-hydroxydopamine administration: a flow cytometric study. J. Neurochem. *53*, 879–885.

44. Kan, I., Ben-Zur, T., Barhum, Y., Levy, Y.S., Burstein, A., Charlow, T., Bulvik, S., Melamed, E., and Offen, D. (2007). Dopaminergic differentiation of human mesenchymal stem cells—utilization of bioassay for tyrosine hydroxylase expression. Neurosci. Lett. *419*, 28–33.

45. Henderson, J.K., Draper, J.S., Baillie, H.S., Fishel, S., Thomson, J.A., Moore, H., and Andrews, P.W. (2002). Preimplantation human embryos and embryonic stem cells show comparable expression of stage-specific embryonic antigens. Stem Cells *20*, 329–337.

46. Abujarour, R., Valamehr, B., Robinson, M., Rezner, B., Vranceanu, F., and Flynn, P. (2013). Optimized surface markers for the prospective isolation of high-quality hiPSCs using flow cytometry selection. Sci. Rep. *3*, 1179.

47. Barbar, L., Jain, T., Zimmer, M., Kruglikov, I., Sadick, J.S., Wang, M., Kalpana, K., Rose, I.V., Burstein, S.R., and Rusielewicz, T. (2020). CD49f is a novel marker of functional and reactive human iPSC-derived astrocytes. Neuron *107*, 436–453.
